# Cell type differences in human cytomegalovirus transcription and epigenetic regulation with insights into major immediate-early enhancer-promoter control

**DOI:** 10.1371/journal.ppat.1013374

**Published:** 2025-08-04

**Authors:** Qiaolin Hu, Ming Li, Mrutyunjaya Parida, Benjamin M. Spector, Juan F. Santana, Arya Zandvakili, David H. Price, Jeffery L. Meier

**Affiliations:** 1 Iowa City Veterans Affairs Healthcare System and University of Iowa Carver College of Medicine, Iowa City, Iowa, United States of America; 2 Department of Internal Medicine, University of Iowa Carver College of Medicine, Iowa City, Iowa, United States of America; 3 Department of Pathology, University of Iowa Carver College of Medicine, Iowa City, Iowa, United States of America; 4 Department of Biochemistry and Molecular Biology^4^ University of Iowa Carver College of Medicine, Iowa City, Iowa, United States of America; Leibniz Institute of Virology (LIV), GERMANY

## Abstract

Cell type differences in the human cytomegalovirus (HCMV) transcriptome may arise from variations in transcription or post-transcription regulation. Here we report unexpected differences in transcription and epigenetic control in late-stage HCMV infection of human differentiated NTera2 neural lineage cells (D-NT2) compared to fibroblasts, using integrated functional genomic approaches (PRO-Seq, RNA-Seq, DNA fragmentation factor-ChIP Seq, rapid viral protein degradation, and promoter mutation and function assays). In D-NT2, but not fibroblasts, RNA polymerase II initiation and elongation at several viral promoters requires viral DNA synthesis and are independent of host P-TEFb, viral immediate-early protein 2 (IE2), or viral late transcription factor (LTF). This includes transcription from the enhancer for the major immediate early (MIE) promoter where GC-box sequence mutations increase enhancer transcription, while mutations in CREB and NF-kB response elements reduce it. The GC-box mutations also alter infected D-NT2 cell morphology and gene expression program without affecting viral MIE gene expression levels, whereas mutations in CREB and NF-kB response elements do not induce these changes. In D-NT2, LTF-driven promoters constitute a smaller proportion of the viral late promoter population and are generally less active. Additionally, viral genomes have more nucleosomes, potentially restricting LTF access. A TATA-binding protein (TBP)-IE2-nucleosome complex, with more nucleosome than in fibroblasts, occupies the MIE promoter transcription start site, potentially contributing to its epigenetic silencing.

## Author summary

Human cytomegalovirus persists silently in most human tissues. Upon reactivation, it infects various cell types, leading to outcomes ranging from asymptomatic shedding to invasive disease, depending on the affected organ and host response. We identify cell type-specific differences in human cytomegalovirus transcription during late-stage infection, revealing novel promoter regulatory mechanisms independent of viral IE2 or late transcription factors. These mechanisms govern multiple viral promoters, including a promoter within the enhancer for the major immediate-early promoter (MIEP). This enhancer-associated promoter is selectively activated in a cell type-dependent manner under cis-regulatory element control, producing a stable enhancer RNA. While histone H3.3, H3 lysine 4 trimethylation, and H3 lysine 27 acetylation are absent from the enhancer, their proportion within the TBP-IE2-nucleosome complex at the MIEP varies by cell type. These structural differences suggest an epigenetic mechanism of MIEP regulation influenced by cell type-specific chromatin architecture.

## Introduction

Human cytomegalovirus (HCMV), a beta-herpesvirus, replicates *in vivo* in a wide variety of differentiated cell types originating from endoderm, mesoderm, or ectoderm, including astrocytes and neuronal cells [1,2]. Variations in HCMV’s transcriptome during viral replication have been observed across cell types [3–5]. This variation may result from cell type-specific differences in viral transcription regulation, RNA processing-stability, or other factors. To directly assess transcriptional changes, precision run-on sequencing (PRO-Seq) [6] and metabolic RNA labelling with chemical nucleotide conversion sequencing (SLAM-Seq) have been developed [7,8]. These technologies have provided new insights into the mechanisms underlying transcriptional control in alpha-, beta-, and gamma-herpesvirus infections [9–13]. PRO-Seq allows for single-nucleotide resolution profiling of RNA polymerase II (Pol II) transcription, enabling direct assessment of transcriptional responses to system changes. PRO-Seq can identify promoters and enhancers producing unstable nascent RNA by capturing actively engaged Pol II, offering a unique perspective on transcriptional dynamics.

PRO-Seq analyses of HCMV in prototypical human fibroblast infections have revealed a distinct pattern of HCMV transcription start site (TSS) usage that shifts significantly after the onset of HCMV DNA replication, ultimately leading to an extensive network of active viral TSSs in late-stage infection [9,14,15]. Additionally, as Pol II productive elongation levels rise, Pol II transiently pausing in the promoter-proximal pause region also increases [9]. The number of nascent RNA 5’-ends precisely mapping to each HCMV TSS serves as a validated proxy for viral promoter TSS strength [9,14,15], with these viral TSS’s exhibiting strengths spanning more than three orders of magnitude, excluding the RNA4.9 promoter [9,14,15].

Coupling PRO-Seq with the targeted protein degradation (a method that uses a small molecule to selectively and rapidly eliminate a viral protein) has opened new avenues for investigating viral transcription regulation. Applying this approach in late-stage HCMV infection of fibroblasts uncovered previously unknown mechanisms of viral transcription control involving the HCMV immediate-early 2 (IE2) proteins [14,16] and members of the HCMV late transcription factor (LTF) family [15]. The HCMV IE2 protein increases transcription at a small subset of viral early-late and late promoters while also down-regulating transcription from the HCMV major immediate-early promoter (MIEP) [14,16]. The MIEP directs the production of IE1–72 and IE2–86, viral proteins that are essential for viral early gene expression and DNA replication. Their expression is subsequently downregulated through negative autoregulation, as IE2–86 and late IE2–60 and IE2–40 isoforms bind to the *cis* repression sequence (*crs*) located immediately upstream of the MIEP TSS [17].

HCMV encodes a six-member set of LTFs that form a complex binding to a TATT sequence in viral promoters, initiating transcription following the onset of viral DNA replication [15,18]. The application of PRO-Seq and targeted LTF degradation during late-stage HCMV infection of fibroblasts revealed that LTFs drive transcription from ~95% of HCMV early-late and late promoters [15]. This promoter activation requires the onset, but not the continuation, of viral DNA replication [15]. The HCMV LTF binding sites exhibit sequence variation, which predicts promoter TSS strength [15].

Cutting edge insights into transcriptional mechanism also emerge from high-resolution analyses of transcription factor and nucleosome occupancies of HCMV genomes [19,20]. The use of human DNA Fragmentation Factor (DFF) endonuclease to digest double-stranded DNA in chromatin, combined with chromatin immunoprecipitation and next-generation sequencing (DFF-ChIP Seq) [19–22], offers unparalleled resolution. This approach surpasses the results obtained from traditional ChIP-Seq, which relies on chromatin fragmentation by sonication or micrococcal nuclease digestion, as well as the CUT&RUN method [19]. At 48 h post-infection (pi) of fibroblasts, shortly after onset of HCMV replication, DFF-ChIP Seq analysis revealed structural distinctions between the viral LTF complex and host TATA-binding protein pre-initiation complex (TBP PIC) on viral genomes [19]. It also uncovered structural interactions between nucleosomes and transcription factors [19], as well as a prominent TBP-viral IE2-nucleosome complex occupying the TATA box, *crs*, and TSS of the MIEP [16], the master switch for HCMV replication [17].

After refining and validating these advanced tools in a prototypical HCMV infection in human foreskin fibroblasts (HFF), we now investigate differences in HCMV transcription regulation and chromatin architecture in a neural lineage cell model. Human NTera2 cells provide a well-established and tractable model for studying how neural lineage cell differentiation influences HCMV gene expression [23]. While undifferentiated NTera2 cells restrict HCMV gene expression, their differentiated neural lineage cell counterparts (D-NT2) support both HCMV gene expression and replication [24,25]. This study was prompted by the initial observation, described herein, of striking differences in HCMV transcription between HFF and D-NT2 in late-stage infection, including the presence of viral enhancer RNA that is absent in fibroblasts. This discovery provided rationale for investigating the mechanisms underlying these differences. In this study, we determine how HCMV transcriptional regulation differ between late-stage infections of fibroblasts and D-NT2 cells; define the roles of viral (IE2, LTFs, and cis-regulatory elements) and host (P-TEFb) factors in governing RNA Pol II activity and viral promoter function in a cell type-dependent manner; and examine how the extent of viral genome chromatization relates to viral late promoter activity and the structure of the MIEP TBP-nucleosome complex.

## Results

### Differences in HCMV transcription start site usage between HFF and D-NT2 in late-stage infection

**Fig 1:**
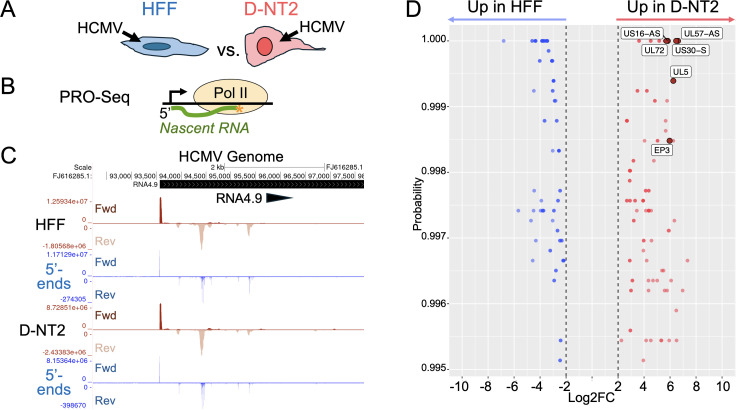
Differences in HCMV transcription start site usage between HFF and D-NT2 at 96 h pi. HFF and D-NT2 were infected in parallel for 96 h, with flavopiridol (Flavo) added during the final 1 h of infection to assess viral transcription start site (TSS) usage (Exp 5, S1 Table). To achieve complete infection of the D-NT2 population, 2.5 times more infectious virus was added to D-NT2 (MOI 7.5, determined in HFF) compared to HFF (MOI 3). (**B**) PRO-Seq was performed for nascent RNA quantification and sequence analysis. The unique molecular identifiers (UMIs)—comprising 16 random nucleotides across the two RNA adapters ligated to each nascent RNA—enables accurate quantification of up to 4.29 billion molecules of RNA with otherwise identical sequences. (**C**) Auto-scaled UCSC Genome Browser views of spike-in normalized nascent RNA reads mapped to the HCMV RNA4.9 promoter region of the annotated HCMV Towne genome (FJ616285.1). The HFF and D-NT2 infections yielded 41.97 million and 28.58 million paired-end, de-duplicated viral reads mapping to the HCMV genome, respectively. Corresponding tracks display the genome base positions (X-axis) and read density of 5’-ends of nascent RNAs (Y-axis), which mark viral TSS sites. (**D**) Differential display of viral TSS strength in HFF vs D-NT2 infections identifies 124 differentially expressed viral TSSs (≥4-fold difference in TSS strength) and their estimated probability of differential expression (probability = 1 minus the p-value ≤0.005). These are represented as blue or red dots. Darker shades indicated overlapping data points. The spike-in normalized HFF and D-NT2 PRO-Seq datasets were adjusted for equivalence in total viral reads across the genome, excluding RNA4.9-derived reads, before performing the differential analysis using the NOISeq program. Six viral TSSs (UL5, UL72, EP3, UL57-AS, US16-AS, and US30-S promoters) that are ≥ 50-fold more active in D-NT2 than HFF are marked by large dark red dots and selected for further study; each TSS is named according to its position relative to the viral gene annotation.

Inspired by PRO-Seq pilot results in D-NT2 neural lineage cells revealing an HCMV late transcription pattern that differed from that in HFF at 96 h post-infection (pi), we directly compared D-NT2 and HFF infected with HCMV (Towne) for 96 h – more than 48 h beyond the onset of HCMV DNA replication (Fig 1A). We employed a single-step viral replication format in which all cells were initially infected. To achieve infection of all cells and comparable HCMV DNA levels in nuclei of both cell types at 96 h pi, we applied 2.5- to 3-fold more infectious units of HCMV to D-NT2 than to HFF (S1A Fig). While acknowledging the caveat that the proportion of HCMV genomes engaged in transcription at 96 h pi (when HCMV DNA is typically packaged into capsid) is unknown and may vary by cell type, we proceeded with PRO-Seq to assess whether the pattern of viral TSS usage differed between D-NT2 and HFF. D-NT2 of neural lineage were derived from undifferentiated NT2 cells by exposure to retinoic acid in fetal bovine serum for 10 days, followed by a one-day washout of the retinoic acid. D-NT2 and HFF were infected in parallel and harvested at 96 h pi for rapid nuclei isolation. During the final hour of infection, cells were treated with flavopiridol (Flavo), an inhibitor of host P-TEFb. Flavo prevents promoter proximal paused Pol II from transitioning into productive elongation. By blocking Pol II progression through downstream promoters, this inhibition enhances the precision of identifying and measuring viral transcription initiation [9,26]. PRO-Seq was performed on infected cell nuclei along with spike-in control *Spodoptera frugiperda* cell nuclei (Fig 1B). De-duplicated nascent RNA reads were aligned to the HCMV, host, and *Spodoptera frugiperda* genomes.

D-NT2 infections yielded 32% fewer spike-in normalized reads mapping to HCMV genome compared to HFF infection, with a 37% lower ratio of HCMV-to-human genome mapped reads in D-NT2 (Exp 5, S1 Table). This reduction is evident in auto-scaled UCSC Genome Browser views of Pol II transcription at the HCMV RNA4.9 promoter region, where the read density of 5’-ends of nascent RNAs at the RNA4.9 promoter TSS is 30% lower in D-NT2 compared to HFF (Fig 1C). Based on normalized read counts, these 5’-ends constitute approximately 47% and 44% of all 5’-ends mapping to the HCMV genome in D-NT2 and HFF, respectively. This indicates that approximately 7% fewer 5’-ends map outside the RNA4.9 promoter region of the HCMV genome in D-NT2 compared to HFF.

A comparison of the 96-h HFF and D-NT2 infection datasets for viral TSSs having at least 200 5’-end reads in either condition identified 124 differentially expressed viral TSSs, showing ≥4-fold differences with a p-value ≤0.005. Among these, 73 were more active in D-NT2, while 51 were more active in HFF (Fig 1D). This pattern was also observed in the differential display analysis of independent PRO-Seq datasets generated from separate 96-h HFF vs D-NT2 infections (S1B Fig). Six promoters—UL5, UL72, EP3, UL57-AS, US16-AS, and US30-S promoters—were selected from the viral TSSs ≥ 50-fold more active in D-NT2 than in HFF for an in-depth investigation of their regulatory mechanisms. These promoters were named based on their positions relative to viral gene annotations. EP3 is now the third MIE enhancer promoter identified, with its TSS located at position -276 relative to the + 1 MIEP TSS. The activation of enhancer EP1 and EP2 promoters during late-stage infection of HFF by viral IE2 and LTFs, respectively, has been described previously [14,15].

### D-NT2 support a unique type of HCMV transcription control that includes the viral MIE enhancer

**Fig 2:**
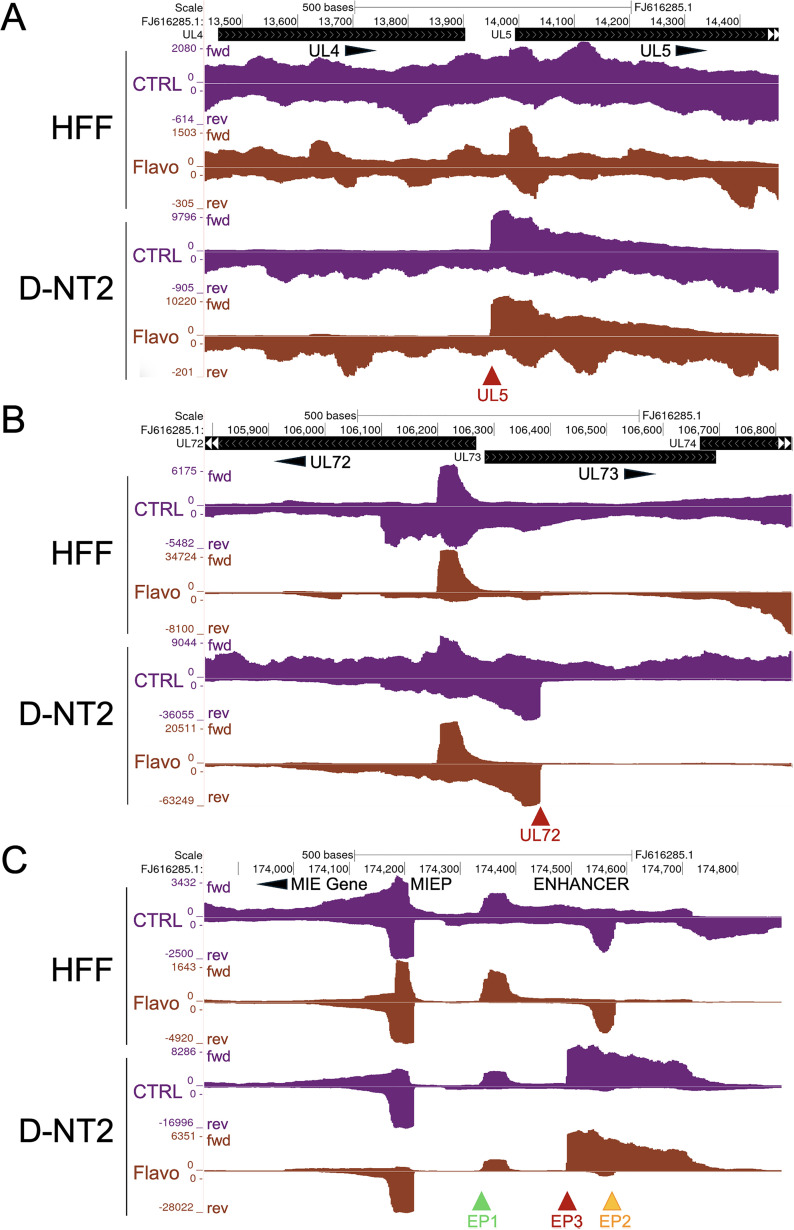
Transcription from a set of viral promoters active in D-NT2 and not HFF is independent on P-TEFb. PRO-Seq was performed at 96 h pi with and without Flavo treatment during the final hour of HFF (GSE139114) and D-NT2 (Exp 2, S1 Table) infections. Auto-scaled UCSC Genome Browser views of spike-in normalized nascent RNA reads, aligned to the annotated HCMV Towne genome (FJ616285.1), display transcriptional activity at the HCMV UL5 (**A**), UL72 (**B**), and MIE enhancer (EP3) (**C**) promoters. These promoters are active in D-NT2 but silent in HFF and are classified as viral long promoters. Dark red arrowheads indicate viral long promoters in D-NT2, while green and gold arrowheads mark EP1 and EP2, respectively. Abbreviations: enhancer promoter 3 (EP3); post-infection (pi); vehicle control (CTRL); forward strand (fwd); reverse strand (rev).

To investigate the mechanisms driving transcription from viral UL5, UL72, EP3, UL57-AS, US16-AS, and US30-S promoters, we applied PRO-Seq with and without Flavo in D-NT2 infected with HCMV for 96 h and compared the results to those from HFF at 96 h pi. UCSC Genome Browser views of UL5 (Fig 2A), UL72 (Fig 2B), and MIE enhancer EP3 (Fig 2C) promoters show that these promoters are active in D-NT2 and negligibly active in HFF. Unlike other viral promoters, Pol II nascent RNA at these sites extends beyond the normal zone of promoter-proximal Pol II pause termination, and this extension is unaffected by Flavo, a P-TEFb inhibitor. We refer to these as viral long promoters because the average length of nascent RNA reads initiating at their TSS (≥69 nucleotides) is greater than that of the other top 500 strongest viral promoters. In contrast to the MIE enhancer EP3 long promoter, the Pol II initiating at MIE enhancer EP1 and EP2 promoters undergoes P-TEFb-driven productive elongation in both HFF and D-NT2 infections. The UL57-AS, US16-AS, and US30-S promoters exhibited similar long promoter characteristics in D-NT2 (S2 Fig). Viral long promoters were not detected in infected HFF.

**Fig 3:**
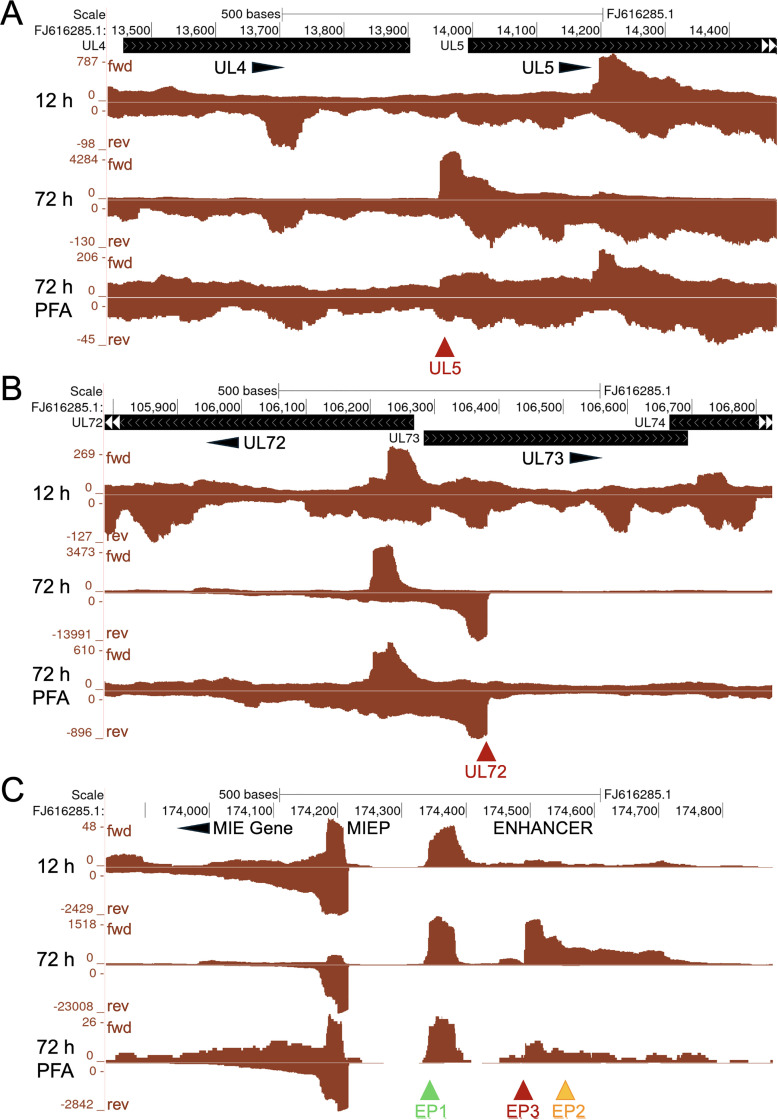
Viral long promoter transcription requires viral DNA replication. (**A-C**) D-NT2 were HCMV-infected for 12 h, 72 h, and 72 h in the presence of PFA, an HCMV DNA replication inhibitor. Flavo was added for the final hour of infection prior to PRO-Seq to identify viral promoters producing nascent RNAs before (12 h) and after (72 h) the onset of viral DNA replication (Exp 1, S1 Table). Auto-scaled spike-in normalized UCSC genome Browser views of nascent RNA reads aligned to annotated HCMV Towne genome (FJ616285.1) viral long promoters UL5 (**A**), UL72 (**B**), and enhancer EP3 (**C**). Dark red arrowheads indicate TSSs of viral long promoters. Abbreviation: phosphonoformic acid, PFA.

To determine whether viral long promoters in D-NT2 depend on HCMV DNA replication, we applied PRO-Seq with Flavo at 12 and 72 h pi, representing time points before and after the onset of HCMV DNA replication, respectively. In a parallel analysis, phosphonoformic acid (PFA) was included throughout the 72-h infection to inhibit HCMV DNA replication. UCSC Genome Browser views indicate that transcription at UL5, UL72, and EP3 long promoters does not begin until after viral DNA replication initiates and is prevented by PFA (Fig 3). The complete dependence on viral DNA replication is a defining feature of viral late promoters. In contrast, the UL57-AS long promoter exhibits characteristics of early-late promoters, as it is active before viral DNA replication but shows substantial increase in activity following DNA replication onset (S3 Fig).

**Fig 4:**
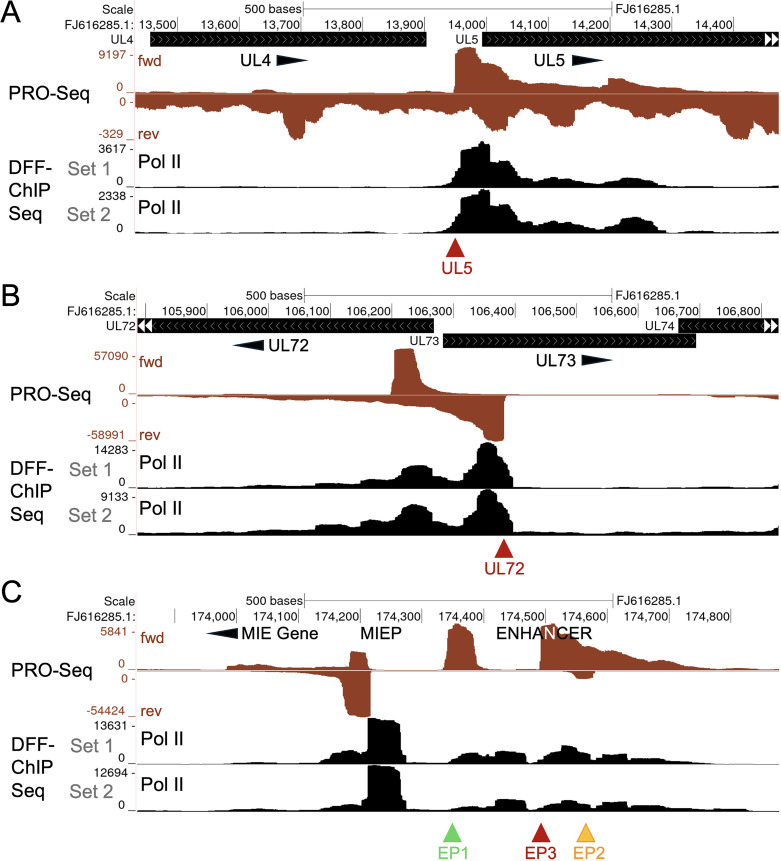
Host RNA Pol II occupies viral long promoters. (**A-C**) D-NT2 were infected for 96 h, and Flavo added for the final hour of infection prior to PRO-Seq to display viral promoters producing nascent RNAs (Exp 5, S1 Table). To determine host RNA Pol II occupancy across the HCMV Towne genome (FJ616285.1), DFF digestion of dsDNA in isolated nuclei was performed, followed by native DFF-ChIP Seq, without prior Flavo (Exp 1, S2 Table). Auto-scaled UCSC genome Browser views show Pol II DFF-ChIP Seq results for 40–65 bp DNA fragments mapping to viral UL5 (**A**), UL72 (**B**), and enhancer EP3 (**C**) regions. Results from 2 independent experiments (Exp 1 and Exp 2, S2 Table), conducted months apart (Sets 1 and 2), are shown. Dark red arrowheads indicate TSSs of viral long promoters.

We next investigated whether host Pol II carries out the unusual type of transcription at viral long promoters. Pol II DFF-ChIP was performed on HCMV-infected D-NT2 at 96 h pi in two independent experiments (sets 1 and 2). UCSC Genome Browser views show pileup distribution of Pol II ChIP fragments over UL5, UL72, and EP3 long promoter regions (Fig 4). The positions of Pol II occupancy corresponded to PRO-Seq signal locations, strongly suggesting that host Pol II is involved in long promoter transcription.

### Viral IE2 protein and LTFs do not drive viral long promoter transcription

**Fig 5:**
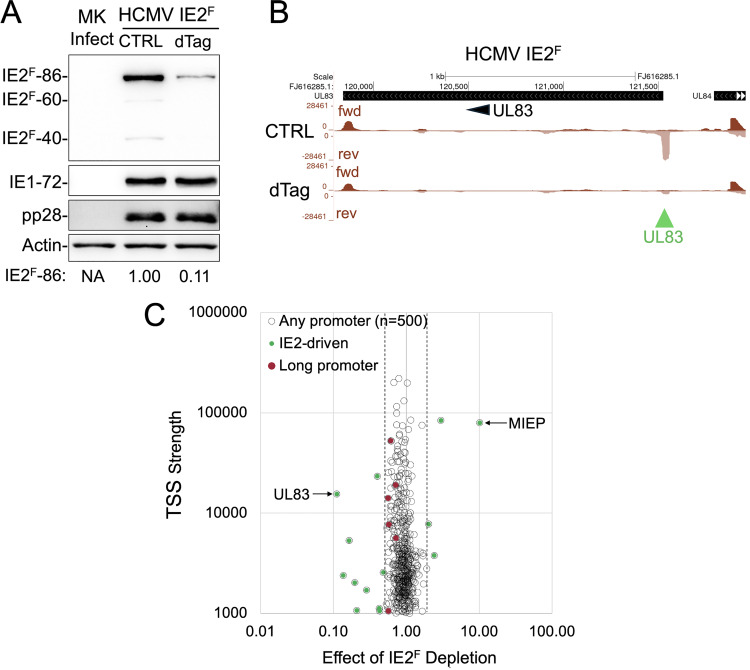
Depletion of viral IE2 in late infection does not affect viral long promoter transcription. D-NT2 were infected for 96 h with HCMV expressing FKBP12^F36V^-tagged IE2–86, IE2–60, and IE2–40 (IE2^F^) and treated with dTag degrader or vehicle control (CTRL) from 90–96 h pi. (**A**) Western blot analysis of whole-cell extracts shows that dTag degrader reduced IE2^F^-86 levels by 89% and virtually eliminated IE2^F^-60 and IE2^F^-40, compared to CTRL when normalized to host actin control. In contrast, viral IE1 and late protein pp28 levels remained unchanged. Mock infection (MK Inf) control included. (**B, C**) PRO-Seq was performed in parallel to quantify transcriptional changes across the HCMV genome. Flavo was added during the final hour of infection. Spike-in normalized nascent RNA reads were aligned to the annotated HCMV Towne genome (FJ616285.1) (Exp 3, S1 Table). (**B**) UCSC Genome Browser views display the effect of dTag vs CTRL on the IE2-dependent UL83 promoter. Vertical scales were adjusted to visualize changes in nascent RNA reads produced by the UL83 promoter. The green arrowhead marks the IE2-driven UL83 promoter position. (**C**) The effect of IE2^F^ depletion on viral TSS strength was analyzed for the top 500 most active viral TSSs (excluding RNA4.9). The change in transcription level at each TSS following dTag treatment (X-axis) was plotted against the TSS strength in the absence of dTag (Y-axis). Viral TSSs that exhibited a ≥ 2-fold increase or ≥50% decrease in strength due to IE2 depletion (demarcated by hatched lines) were classified as IE2-responsive and are indicated by green dots. These match the viral promoters affected by IE2 depletion in HFF. Arrows indicate the MIEP and UL83 TSSs, which increased 10.1-fold and decreased 98%, respectively, in response to IE2 depletion. Viral long promoters, marked by red dots, showed <50% transcriptional change upon IE2 depletion.

HCMV IE2 and LTFs are the only viral proteins known to activate HCMV promoters during late-stage infection. During this phase, viral late promoters generate the IE2–60 and IE2–40 proteins, while the MIEP produces IE2–86 [17]. All three IE2 proteins share identical carboxy terminal region that binds the *crs* within the MIEP [17] and can accommodate the FKBP12^F36V^ degron tag [14]. To determine whether IE2 is involved in viral long promoter transcription, we used the targeted degradation approach to rapidly deplete FKBP12^F36V^-tagged IE2 proteins (IE2^F^-86, IE2^F^-60, and IE2^F^-40) [14] in infected D-NT2 between 90–96 h pi. We then used PRO-Seq to assess the effect of IE2 depletion on the strength of the 500 most active viral promoters across the HCMV genome. The dTag degrader reduced IE2^F^-86 level by 89% and virtually eliminated IE2^F^-60 and IE2^F^-40, compared to vehicle control, while levels of IE1–72 and late protein pp28 remained unchanged (Fig 5A). The decrease in IE2^F^ proteins resulted in an 89% reduction in viral early-late UL83 promoter transcription (Fig 5B and 5C), consistent with its known activation by IE2 [14]. As expected, IE2^F^ depletion caused 10-fold increase in MIEP transcription (Fig 5C). However, transcription from the viral long promoters had not appreciably changed. These findings were independently reproduced in another experiment using the same methodology (S4 Fig).

**Fig 6:**
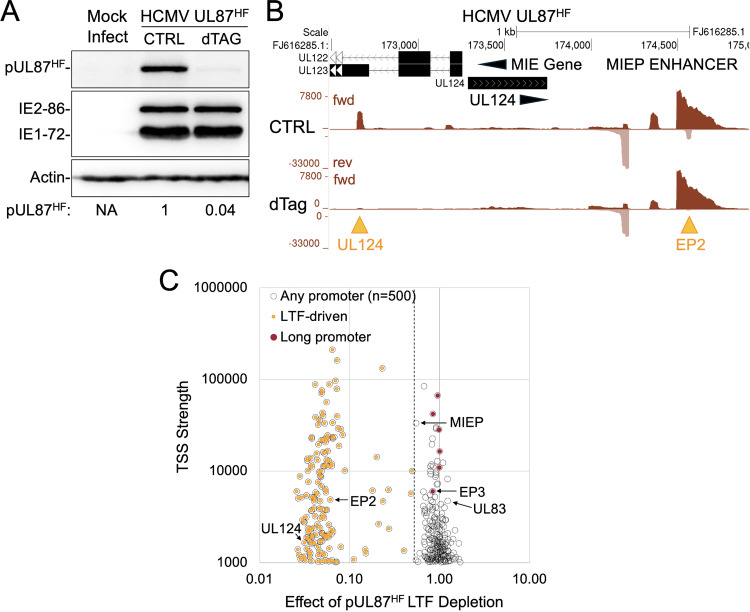
Depletion of viral UL87 LTF in late infection does not affect viral long promoter transcription. D-NT2 were infected for 96 h with HCMV expressing HA-FKBP12^F36V^-tagged pUL87 (pUL87^HF^) and treated with dTag degrader or vehicle control (CTRL) from 90-96 h pi. (**A**) Western blot analysis of whole-cell extracts shows that dTag degrader reduced pUL87^HF^ levels by 96%, compared to CTRL, when normalized to host actin control. In contrast, IE1–72 and IE2–86 levels remained unchanged. (**B, C**) PRO-Seq coupled with Flavo was performed to quantify transcriptional changes across the viral genome. Spike-in normalized nascent RNA reads were aligned to the annotated HCMV genome (FJ616285.1) (Exp 6, S1 Table). **(B)** UCSC Genome Browser of the MIE promoter/enhancer and downstream region show that dTag degrader treatment significantly decreased transcription from the LTF-dependent UL124 promoter and enhancer promoter EP2 (gold arrowheads). In contrast, EP3 long promoter activity remained unaffected. Vertical scales were adjusted to compare nascent RNA levels in transcription start regions. (**C**) The effect of pUL87^HF^ depletion on viral TSS strength was analyzed for the top 500 viral TSSs (excluding RNA4.9). The change in transcription level at each TSS following dTag treatment (X-axis) was plotted against the TSS strength in absence of dTag (Y-axis). Viral TSSs that decreased by ≥50% (demarcated by hatched lines) were classified as pUL87^HF^-dependent and are marked with gold dots.

We applied the same approach to assess whether HCMV UL87 LTF, the viral TBP equivalent, is involved in viral long promoter transcription. The dTag degrader reduced FKBP12^F36V^-tagged UL87 LTF (pUL87^HF^) [14,15] by 96% (Fig 6A), resulting in a 94% and 97% reduction in transcription from the UL124 early-late promoter and late EP2 promoter, respectively (Fig 6B and 6C). In contrast, depletion of pUL87^HF^ had no appreciable effect on transcription from viral long promoters (Fig 6C). These results were confirmed in an independent experiment (S5 Fig). We conclude that viral long promoter transcription is regulated by a mechanism that does not involve viral IE2 or LTFs.

### HCMV MIE enhancer cis-regulatory elements govern EP3 long promoter transcription with differential effects on cell morphology and gene expression

We hypothesized that cis-regulatory elements govern viral long promoter activity and that identifying these elements would provide mechanistic insight into long promoter regulation. To test this, we focused on the EP3 long promoter and three classes of candidate cis-regulatory elements within the MIE enhancer, based on exploratory data suggesting their involvement. The candidate elements include GC-boxes, as well as the combination of CREB (cyclic AMP response elements, CRE) and NF-kB binding sites (kB), which are known to cooperate in activating the MIEP in undifferentiated NTera2 cells in response to protein kinase C signaling [27]. The proximal MIE enhancer contains two GC-boxes (N1 and N2) located 206 and 223 bp upstream of the EP3 TSS. The CRE and kB elements are located ≥10 bp upstream and ≥25 bp downstream of the EP3 TSS.

**Fig 7:**
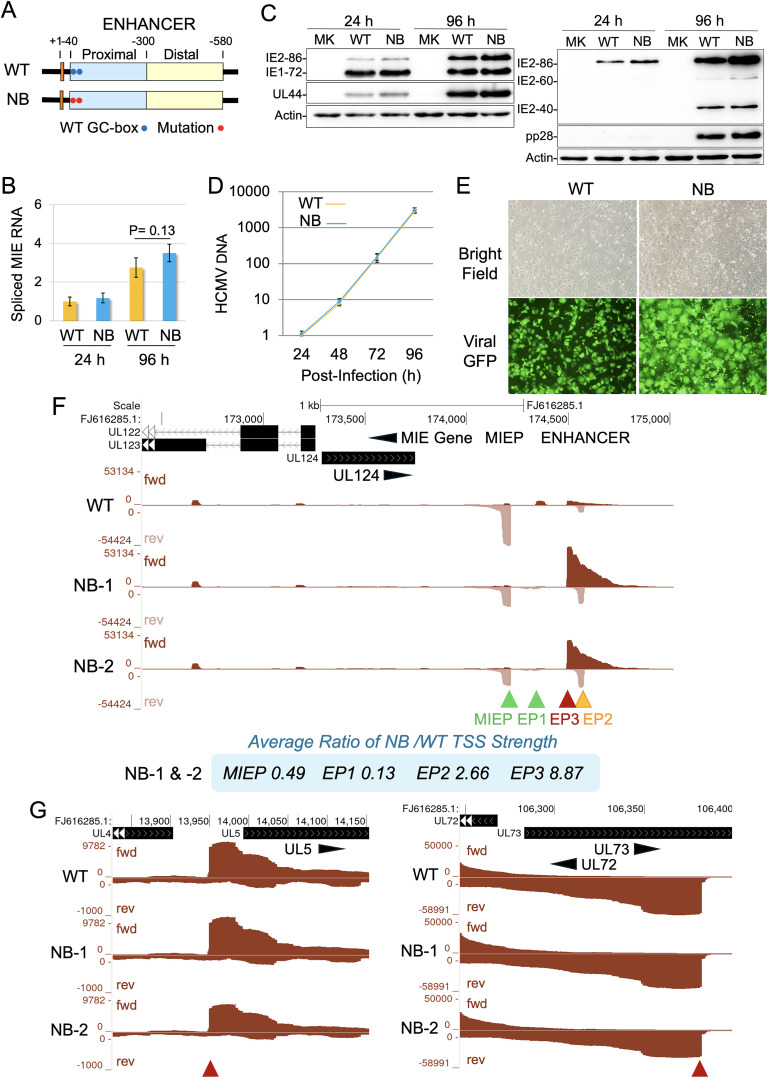
Base-substitutions mutations in MIE enhancer GC-boxes increase EP3 long promoter activity. (**A**) The HCMV NB virus differs from the HCMV WT parent virus (Towne) by carrying base-substitution mutations in two GC-boxes at positions -52 and -69 in the proximal MIE enhancer (see S7 Fig for details). (**B-G**) D-NT2 were infected in parallel with WT and NB viruses. (**B, D**) Levels of the indicated RNA and DNA from triplicate infections (MOI 0.5) were measured by RT-PCR and PCR using the standard curve method and normalized to host GAPDH RNA or DNA, respectively. Spliced MIE RNA levels were measured at 24 and 96 h pi (**B**) and HCMV DNA levels were assessed at 24, 48, 72, and 96 h pi (**D**). (**C**) Levels of IE1–72 and IE2–86 proteins, viral early UL44 protein, and late pp28, IE2–60, and IE2–40 proteins were measured at 24 and 96 h pi by western blot (MOI 5). (**E**) Cell morphology and viral GFP fluorescence intensity were assessed at 96 h pi by fluorescence microscopy (MOI 5). (**F, G**) In a separate set of 96-h D-NT2 infections, WT virus was compared with two independently constructed NB viruses (NB-1 and NB-2) carrying identical GC-box mutations. Spike-in normalized nascent RNA reads were aligned to the HCMV Towne genome (FJ616285.1), except that nascent RNA reads from NB-1 and NB-2 infections were aligned to an HCMV Towne genome incorporating NB mutations (Exp 5, S1 Table). UCSC Genome Browser views show the effect of GC-box mutations on transcription at MIEP, EP1, EP2, and EP3 (**F**), and impact of the mutations on viral UL5 and UL72 long promoter transcription (**G**). Inset (**F**) displays the average ratio of NB/WT TSS strength for MIEP, EP1, EP2, and EP3 promoters.

To determine whether the GC-boxes affect EP3 transcription, we constructed HCMVs with base-substitution mutations in one (N1 or N2) or both (NB) GC-boxes (S6A-S6C Fig). In HFF at MOI 1.0, these mutant viruses produced normal levels of spliced MIE RNA and IE1–72 and IE2–86 proteins at 24 h pi, comparable to the WT parent virus (S6D and S6E Fig). The rate of DNA replication of these viruses over 96 h was similar between WT and mutant viruses (S6F Fig). Comparisons of NB and WT viruses in D-NT2 at 24 and 96 h pi revealed that both viruses produced equivalent levels of spliced MIE RNA and IE1–72, IE2–40, IE2–60, IE2–86, pUL44, and pp28 proteins (Fig 7A-C). WT, NB, N1, and N2 viruses all replicated viral DNA at similar rates and levels over 96 h (Figs 7D and S7A). However, NB-infected D-NT2 exhibited marked changes in morphology and viral GFP fluorescence intensity at 96 h pi (Figs 7E and S7B), whereas N1 and N2 showed milder differences from WT-infected D-NT2 (S7B Fig). These morphological changes were not visible in infected HFF (S7B Fig). To compare transcription between WT and GC-box mutant viruses, we performed PRO-Seq on two independently generated NB viruses (NB-1 and NB-2). The GC-box mutations reduced MIEP and EP1 TSS strengths by 51% and 87%, respectively, while increasing EP2 TSS strength 2.7-fold (Fig 7F). The most significant change in transcription was an 8.9-fold increase in EP3 TSS strength, whereas other viral long promoters exhibited <20% change (Fig 7G). HCMVs with single GC-box mutations (N1 or N2) showed less pronounced increases in EP3 TSS strength compared to NB virus (S7C Fig).

**Fig 8:**
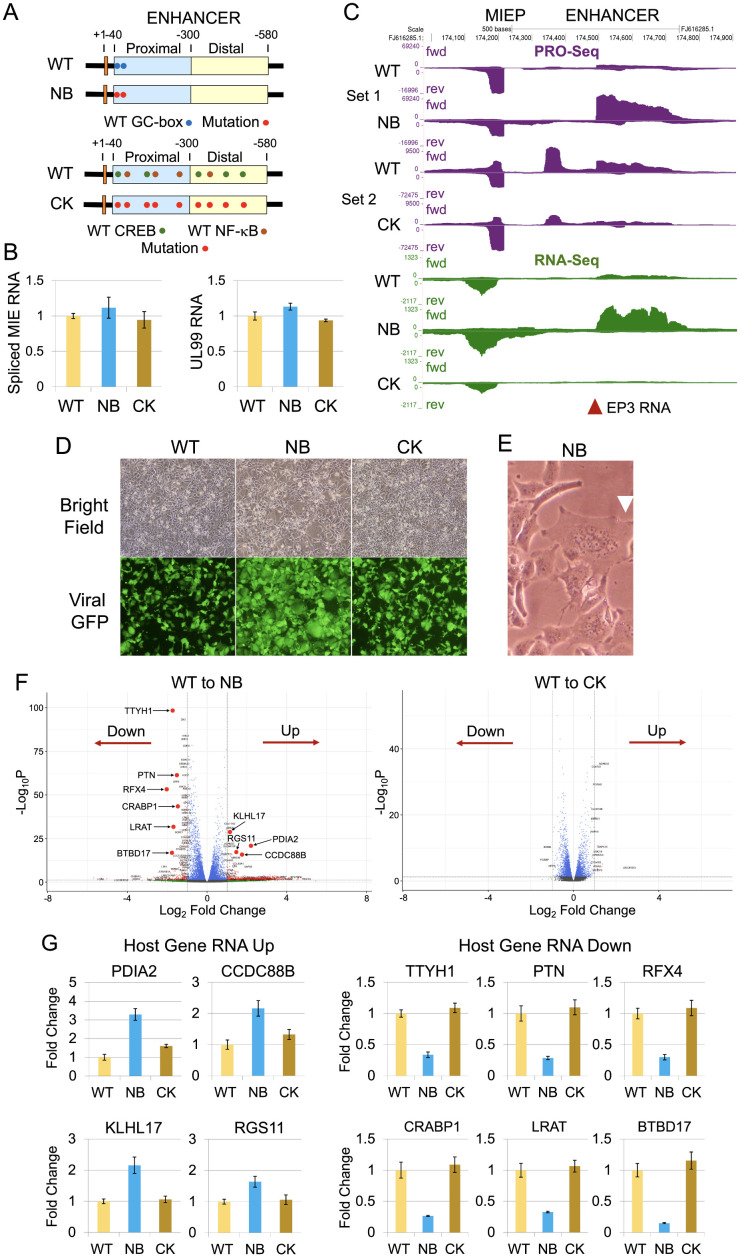
Mutations in CREB and NF-kB response elements decrease EP3 RNA production without altering cell morphology and gene expression, opposite to GC-box mutations. (**A**) HCMV NB and CK viruses differ from HCMV WT parent virus (Towne) only by base-substitutions in the MIE enhancer. NB virus carries mutations in two GC-boxes (base positions -52 and -69) in the proximal MIE enhancer, whereas the CK virus has mutations in five CREB (base positions -60, -135, -320, -403, and -457) and four NF-kB (base positions -95, -158, -265, and -415) binding sites distributed across proximal and distal MIE enhancer segments. (**B-G**) D-NT2 were infected for 96 h with WT, NB, and CK viruses. Infections and analyses of the three viruses was conducted in parallel, except for PRO-Seq analysis (**C**) (Set 1 Exp 2 and Set 2 Exp 4, S1 Table). RNAs from duplicate infections were quantified by RT-PCR using standard curve method and normalized to host GAPDH RNA level (**B, G**) and analyzed by RNA-Seq, with cDNA generated using random primers during reverse transcription (**C, F**) (S3 Table). (**B**) Levels of spliced MIE gene RNA and unspliced late UL99 gene RNA were compared among WT, NB, and CK viruses. (**C**) UCSC Genome Browser views of PRO-Seq (without Flavo, spike-in normalized) and RNA-Seq (normalized total reads mapping to HCMV genome) reads aligned to WT genome or genomes with incorporated enhancer mutations. Scales were set to compare MIEP and EP3 RNA levels. These differences correlate with variations in nascent RNA production at the EP3 TSS (-275) as determined by PRO-Seq with Flavo (S8 Fig). EP1 RNA was undetectable by RNA-Seq. (**D**) Live cell imaging by fluorescence microscopy was performed to assess D-NT2 morphology and viral GFP fluorescence intensity (original magnification, 10x). (**E**) Cell nuclei clustering in syncytia was observed in NB virus-infected D-NT2. (**F, G**) Host gene expression changes were analyzed by RNA-Seq differential expression analysis (**F**) and quantitative RT-PCR (**G**).

To determine whether CRE and kB cis-regulatory elements influence EP3 transcription, we constructed an HCMV with CRE and kB site mutations (CK virus), using the same viral DNA backbone as the NB virus (S8A-S8C Fig). Comparison of CK and WT viruses revealed no differences in MIE RNA expression and viral DNA replication in HFF or D-NT2 (Figs 8A and 8B, and S8D-S8F). PRO-Seq and RNA-Seq analyses of D-NT2 infections with CK, NB, and WT viruses at 96 h consistently showed that the NB virus increased EP3 RNA expression, while the CK virus decreased it (Fig 8C). PRO-Seq plus Flavo analyses at 96 h pi showed that EP3 TSS strength decreased 89% in CK-infected cells, whereas MIEP and EP2 TSS strengths increased slightly (S8G Fig). The NB virus increased D-NT2 enlargement, viral GFP fluorescence intensity (Fig 8D), and formation of multi-nucleated syncytia (Fig 8E) that was not observed in CK-infected cells. Additionally, RNA-Seq and RT-PCR analyses, revealed that NB-infected D-NT2 exhibited a distinct host gene expression pattern compared to CK- and WT-infected cells (Fig 8F and 8G). Thus, in HCMV-infected D-NT2, GC-box mutations increase EP3 RNA production and alter both cell morphology and host gene expression, whereas CK mutations decrease EP3 transcription without affecting these phenotypes.

### HCMV LTF-driven promoters comprise a smaller proportion of the viral late promoter population in D-NT2

Having observed reduced transcription initiation at several LTF-dependent viral promoters in D-NT2 compared to HFF in UCSC Genome Browser tracks, we next quantitatively assessed whether the proportion of viral late promoters driven by viral LTFs differs between the two cell types. Prior PRO-Seq analyses of HCMV-infected HFF revealed that HCMV LTFs drive transcription at most HCMV promoters that rely on viral DNA replication [15]. Notably, a substantial fraction of these LTF-activated promoters lack the classic TATT sequence [15]. To quantify the degree to which viral TSS strength depends on viral DNA replication, we used the PFA sensitivity index (PSI), calculated as the ratio of viral TSS strength gained during viral DNA replication to the TSS strength remaining after inhibition with PFA [14,15]. Studies in HFF, established that the PSI score of the viral early-late UL83 promoter TSS serves as a threshold for distinguishing early (immediate-early, early, and early-late) from late viral promoters [14].

**Fig 9:**
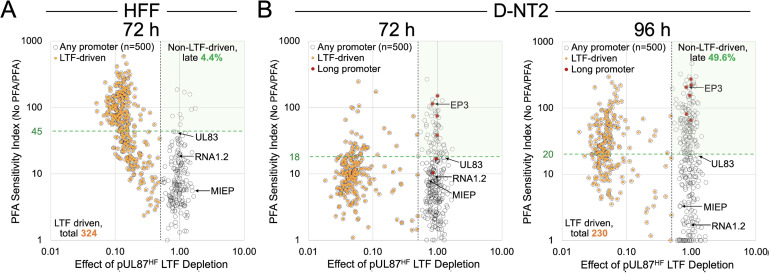
HCMV LTF-driven promoters constitute a smaller proportion of viral late promoters in D-NT2. PRO-Seq coupled with Flavo was used to quantify 5′-ends of viral nascent RNA at viral TSSs. The PFA Sensitivity Index (PSI), calculated as TSS strength without PFA/ TSS strength with PFA, measures dependence on viral DNA replication. In HFF, the PSI for the early-late UL83 promoter TSS serves as the threshold distinguishing early-late from late viral promoters (GSE168165). The UL83 TSS PSI value in HFF at 72 h pi is 45, whereas in D-NT2 at 72 and 96 h pi, it is 18 and 20, respectively (hatched green lines) (Exp 1 and 4, S1 Table). The RNA4.9 region was excluded from analysis. (**A, B**) Scatterplots show PSI values for the top 500 most active viral TSSs against changes in TSS strength following pUL87 LTF depletion in HFF at 72 h pi (**A**) (GSE168165) and D-NT2 at 72 and 96 h pi (**B**) (Exp 1, 4 and 6, S1 Table). LTF-driven promoters (orange dots) were defined as those exhibiting a ≥ 50% decrease in TSS strength upon LTF depletion. LTF-driven promoters account for 324 of 500 viral TSSs in HFF at 72 h pi but only 230 of 500 viral TSSs in D-NT2 at 96 h pi. Non-LTF-driven late promoters (in light green area) account for 4.4% and 49.6% of the viral late promoter populations (late pop., above the green hatched line) in HFF at 72 h and D-NT2 at 96 h pi, respectively.

We applied this classification scheme to D-NT2 infections by calculating PSI scores and plotting them against the effect of pUL87 LTF depletion across the 500 most active viral TSSs (Fig 9). In HFF at 72 h pi, the viral UL83 promoter TSS exhibited a PSI of 45. In contrast PSI scores for the same UL83 promoter TSS were substantially lower in D-NT2, with scores 18 and 20 at 72 and 96 h pi, respectively. This suggests that viral DNA replication (which occurs in comparable levels in D-NT2 and HFF) plays a comparatively smaller role in enhancing UL83 TSS strength in D-NT2 than in HFF, a pattern that extends to many other early-late and late viral promoters. In D-NT2, most LTF-driven TSSs did not surpass the PSI threshold distinguishing late from early viral promoters until 96 h pi. Compared to HFF at 72 h pi, the 96-h D-NT2 infection exhibited a 29% reduction in active LTF-driven TSSs (324 vs 230 LTF-driven viral TSSs) and an 11-fold increase in the proportion of viral late TSSs that are LTF-independent (4.4 vs 49.9% of non-LTF driven late viral TSSs). The UL83 TSS was one of the only three non-LTF-driven late viral TSSs whose transcription was reduced following a 6-h depletion of viral IE2. The viral long promoters also fall in the non-LTF-driven late promoter group. Notably, while a subset of the LTF-driven TSSs retained comparable strengths between HFF and D-NT2, the majority showed reduced strength in D-NT2 (S9 Fig), highlighting the widespread attenuation of LTF-mediated transcription in D-NT2.

### Greater nucleosome occupancy of HCMV genomes in D-NT2

**Fig 10:**
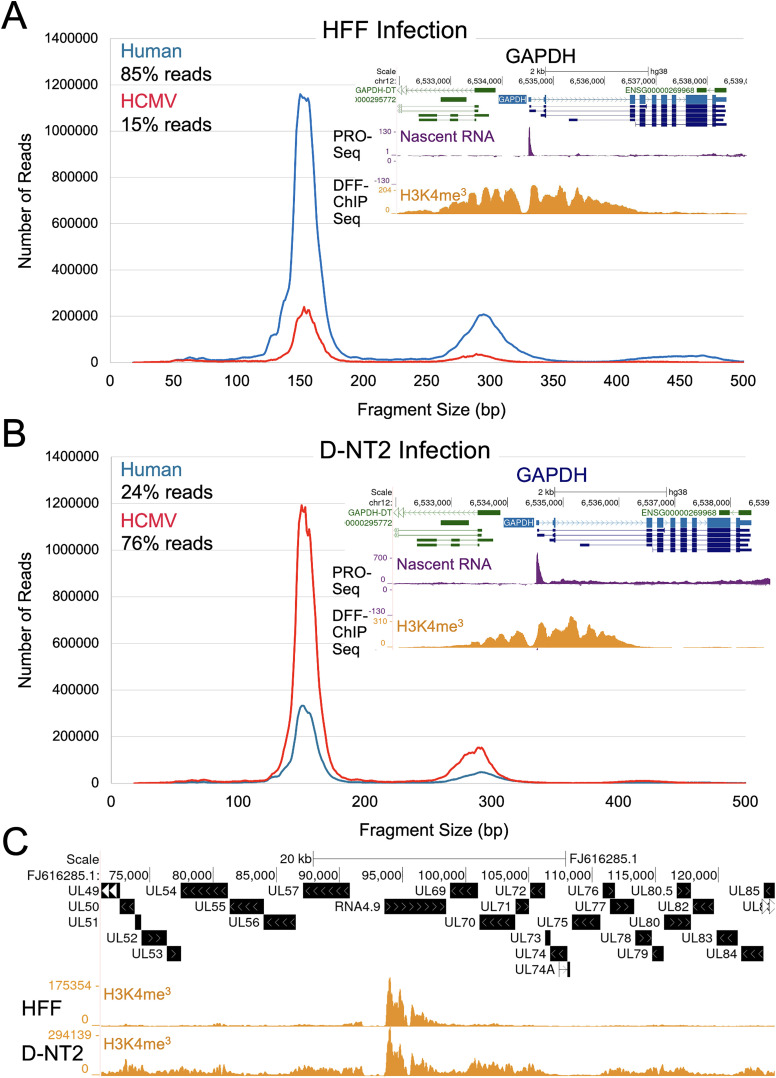
Increased H3K4me3 histone occupancy of nucleosome-sized DNA segments of HCMV genomes in D-NT2 compared to HFF. (**A-C**) Native DFF-ChIP for H3K4me3 was performed on HFF (**A, C**) and D-NT2 (**B, C**) infected in parallel for 96 h (Exp 4, S2 Table). DNA fragment reads were aligned to the host (human hg38) and HCMV Towne (FJ616285.1) genomes. Graphs display the number of fragment reads and fragment sizes (18–500 bp) for host (blue) and HCMV (red) genome fragments. Total viral reads were normalized to account for a 65% difference in GAPDH-normalized viral DNA quantities in HFF vs D-NT2 nuclei at 96 h pi. Percentages of host and HCMV genome reads are indicated. Insets show UCSC Genome Browser snapshots of H3K4me3 DFF-ChIP Seq results for the host GAPDH gene, aligned with PRO-Seq nascent RNA reads. (**C**) UCSC Genome Browser displays H3K4me3 occupancy across a 50-kbp HCMV genome region containing RNA4.9 in HFF vs D-NT2 infections.

Nucleosomes may impede HCMV LTF binding and function [9,15]. To assess H3K4me3 histone occupancy on HCMV genomes in HFF vs D-NT2, we performed native DFF-ChIP Seq for H3K4me3 histone in parallel infections at 96 h. Of the H3K4me3 DFF-ChIP fragments mapped to host and HCMV genomes, 15% mapped to HCMV genomes in HFF, whereas 76% mapped to HCMV genomes in D-NT2 (Fig 10 and S2 Table). UCSC Genome Browser views of H3K4me3 histone occupancy on the host GAPDH gene confirmed comparability between datasets (Fig 10A and 10B). Line graphs plotting fragment count vs DNA fragment size revealed higher levels of the H3K4me3 histone mark on nucleosome-sized DNA segments of HCMV genomes (~155-bp-sized DNA fragments) in D-NT2 compared to HFF (Fig 10A and 10B). A UCSC Genome Browser view of a 55-kb segment of HCMV genome showed that histone H3K4me3 occupancy outside the RNA4.9 gene region was substantially higher in D-NT2 compared to HFF (Fig 10C). In a separate experiment comparing cross-linked vs native DFF-ChIP Seq, paraformaldehyde-crosslinking of infected D-NT2 before DFF-ChIP did not prevent higher H3K4me3 histone occupancy on nucleosome-sized HCMV DNA fragments in D-NT2 (S10 Fig).

**Fig 11:**
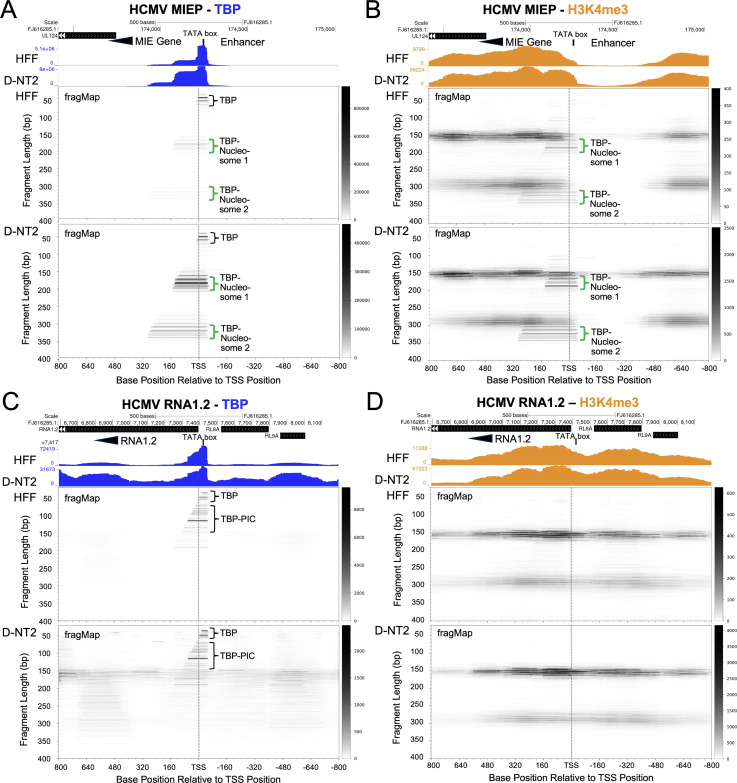
Increased nucleosome proportion in the HCMV MIEP TBP-nucleosome complex in D-NT2. Native DFF-ChIP Seq was performed on HFF and D-NT2 infected for 96 h. (**A, C**) UCSC Genome Browser views (top) and fragMaps (bottom) display TBP occupancy at the MIEP (**A**) and RNA1.2 promoter (**C**). (**B, D**) Corresponding H3K4me3 occupancy patterns for the MIEP (**B**) and RNA1.2 promoter (**D**). Data span -800 bp upstream to +800 bp downstream of the TSSs for each promoter. FragMaps illustrate DNA fragment distributions by length (left vertical axis) and relative abundance (right vertical axis) mapped to base coordinates of the MIEP and RNA1.2 promoter regions (horizontal axis). Datasets were generated from parallel HFF and D-NT2 infections, cell preparation, DFF-ChIP, library construction, and Illumina sequencing. Green bracket marks TBP-nucleosome fragments, and black bracket marks TBP or TBP-PIC fragments.

A previous DFF-ChIP study conducted in HFF was the first to demonstrate that a TBP-nucleosome complex occupies the HCMV MIEP at 48 h pi [16]. To investigate whether this structure changes later in HFF infection or differs in D-NT2 infection, we applied the DFF-ChIP method to assess TBP and H3K4me3 histone occupancy of HCMV genomes in HFF vs D-NT2 at 96 h pi. Regardless of cell type, nearly 30% of the TBP DFF-ChIP fragments mapping to the HCMV genome were positioned at the MIEP (Fig 11A). However, the pileup of H3K4me3 DFF-ChIP fragments at the MIEP revealed distinct profiles between HFF and D-NT2 (Fig 11B). In both cell types, H3K4me3 signal was sparse over the MIE enhancer. FragMaps displaying fragment lengths, amounts, and positions in the MIEP region show distinct groups of fragments corresponding to free TBP and a TBP-nucleosome complex (TBP-nucleosome 1), as well as a TBP-nucleosome complex linked to the adjacent downstream nucleosome (TBP-nucleosome 2). The ratio of free TBP to TBP-nucleosome 1 complex differed ninefold between HFF and D-NT2 (Fig 11A), suggesting that nucleosomes constitute a greater proportion of the MIEP TBP-nucleosome complex in D-NT2. In contrast, fragMaps for the HCMV RNA1.2 promoter demonstrate that this viral promoter lacks a TBP-nucleosome complex (Fig 11C and 11D). The RNA1.2 promoter has free TBP and TBP fragments corresponding to a TBP-PIC, with comparable amounts in HFF and D-NT2.

### A TBP-IE2-nucleosome complex with H3K4me3 and H3K27Ac modifications occupies the MIEP TSS in D-NT2

**Fig 12:**
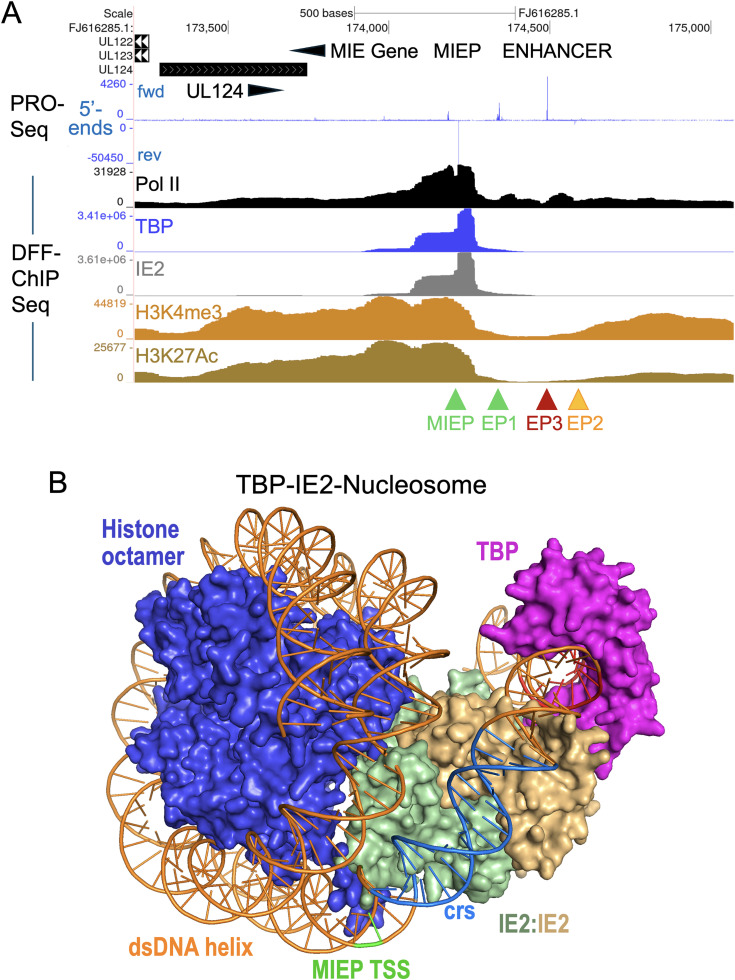
TBP-IE2-nucleosome complex with H3K4me3 and H3K27Ac occupies the MIEP TSS. (**A**) D-NT2 infected for 96 h by HCMV were subjected to native DFF digestion, then divided for Pol II, TBP, IE2, H3K4me3, and H3K27Ac ChIP Seq (Exp 3, S2 Table). Auto-scaled UCSC Genome Browser views display native DFF-ChIP Seq results for the HCMV MIE regulatory region, aligned along with PRO-Seq (with Flavo) results displaying locations of 5’-ends of nascent RNAs. Arrowheads point to TSS positions for MIEP, EP1, EP2, and EP3. (**B**) Structural model of the TBP-IE2-nucleosome complex incorporating HCMV MIEP DNA, generated using AlphaFold 3 and PyMOL software. The nucleosome assembly was simulated using the histone octamer and the Widom-601 dsDNA sequence. Shown are MIEP TSS (green) on dsDNA (orange) in relation to TBP (magenta), IE2 dimer (yellow and darker green), histone octamer (dark blue), and nucleotide positions of TATA box (red) and the *crs* (light blue).

To further define structural components involved in TBP-nucleosome formation in D-NT2, we conducted native DFF-ChIP Seq for Pol II, TBP, IE2, H3K4me3, and H3K27Ac in D-NT2 infected for 96 h. ChIPs were performed in parallel using the same pool of DFF-digested chromatin and were conducted in duplicate, except for H3K27Ac. Auto-scaled UCSC Genome Browser views of DFF-ChIP Seq results were compared to PRO-Seq results, which mapped 5’-ends of nascent RNAs for MIEP, EP1, and EP3 (Fig 12A). Visualization of EP2 was obscured by the high number of MIEP TSS reads. The DFF-ChIP results revealed substantial Pol II occupancy in the promoter-proximal region of MIEP, with small Pol II peaks evident in the MIE enhancer. TBP and viral IE2 exhibited strong, nearly identical occupancy profiles at the MIEP, with a prominent peak spanning +4 to -49 bp relative to the MIEP TSS. This region encompasses the MIEP TATA box, IE2-binding site, and initiator element. The fragMaps for TBP and IE2 displayed strikingly similar features, producing indistinguishable fragment groups corresponding to TBP-IE2 and TBP-IE2-nucleosome complexes (S11 Fig). The TBP-IE2 ChIP signals closely abutted with or overlapped a nucleosome containing H3K4me3 and H3K27Ac histone marks (Fig 12A). FragMaps for H3K4me3 and H3K27Ac showed highly similar DNA fragment profiles, consistent with a stable MIEP TBP-nucleosome complex (S12A Fig) that is not present at the RNA1.2 promoter (S12B Fig). In a separate DFF ChIP-Seq experiment comparing HFF and D-NT2 infections at 96 h pi, the histone H3.3 variant and H3K4me3 were found to co-occupy the MIEP but their levels relative to TBP at the MIEP were lower in HFF compared to D-NT2 (S12C Fig). The proportion of H3.3 reads mapping to the HCMV genome relative to the human genome were approximately 3-fold higher in D-NT2 compared to HFF (Exp 5, S2 Table). A UCSC Genome Browser view of a 70-kb segment of HCMV genome showed that histone H3.3 occupancy relative to the RNA4.9 gene region was substantially higher in D-NT2 compared to HFF (S12D Fig). Notably, H3.3, H3K4me3, and H3K27Ac were nearly absent from the MIEP enhancer region (Figs 12A, and S12A and S12B). These findings indicate that viral IE2 is an integral component of the TBP-nucleosome complex, which carries H3K4me3 and H3K27Ac modifications. We propose that the TBP-IE2-nucleosome complex, modeled in Fig 12B, attenuates transcription at the MIEP, possibly by restricting access of the host general transcription factor TFIIB to DNA encompassing the MIEP TSS, while retaining the capacity to permit transcription depending on specific combinations of histone marks. Host TFIIB interacts with TBP and downstream DNA, recruits Pol II, and is required for transcription at most Pol II-driven promoters in human cells [28].

## Discussion

Our study comparing D-NT2 infection with the prototypical HFF infection focuses on HCMV promoters that become active or remain active more than 48 h beyond the onset of HCMV DNA replication, corresponding to the later stage of late infection. A prior dSLAM-Seq study of murine cytomegalovirus found that additional viral late promoters become active during the later phase of infection [12]. During this stage, we identified a subset of viral promoters that are ≥ 4-fold more active in D-NT2 than in HFF. Promoter activity was determined by quantifying the number of nascent RNAs originating from the strongest TSS of each promoter. Most promoters in this subset are inactive or minimally active in HFF. Six notable promoters within this group exhibit a distinct transcriptional mechanism, characterized by noncanonical transcription initiation and elongation control. These promoters share the feature of Pol II elongation beyond the promoter-proximal pause zone without requiring P-TEFb. They were termed long promoters because the average length of nascent RNAs originating from their TSS exceeds that of viral promoters subject to P-TEFb-dependent elongation control. Viral long promoters align with the operational definition of early-late or late viral promoters, based on nascent RNA detection before and/or after onset of viral DNA replication and the extent to which viral DNA replication amplifies TSS strength. Unlike other viral late promoters, transcription initiation at viral long promoters is not driven by viral IE2 or LTFs, and these promoters lack DNA sequences for LTF binding. This noncanonical mode of viral transcription regulation is exemplified by the EP3 long promoter in the MIE enhancer. Cis-regulatory elements within the enhancer activate EP3 transcription, whereas mutations in CREB and NF-kB binding sites nearly eliminate EP3 transcription initiation. The CREB and NF-kB binding sites are positioned ≥10 bp upstream and ≥25 bp downstream of the EP3 TSS, but the specific site(s) responsible for EP3 activation remains unknown. Cell type-specific combinations of transcription factors that bind the enhancer cis-regulatory elements may govern EP3 transcription.

The discovery that base-substitution mutations in GC-boxes located 206 and 223 bp upstream of the EP3 TSS do not reduce IE1 and IE2 gene expression or viral DNA replication in HFF conflicts with the findings of Isomura et al [29]. Differences in HCMV construct design may account for these discrepancies. Isomura et al. [29] introduced a small insertion in the proximal enhancer while modifying GC-box sequence. Our validation studies in HFF support the findings in D-NT2, showing that disruption of two GC-boxes increases EP3 RNA production without reducing viral IE1–72 and IE2–86 or viral DNA replication. RNA-Seq data indicate that EP3 RNA is stable, whereas EP1 nascent RNA is unstable. The GC-box mutations increase the formation of multinucleated syncytia in D-NT2, a phenomenon not observed in infected HFF. Additionally, GC-box mutations alter the D-NT2 gene expression program, whereas mutations in CREB and NF-kB binding sites decrease EP3 RNA level and do not appreciably affect infected D-NT2 morphology or gene expression pattern. It remains unclear whether the increase in EP3 RNA expression is responsible for the morphological and gene expression changes in infected D-NT2. The EP3 RNA, approximately 220 nucleotides in length, is unlikely to encode a protein [30]. It was identified using an RNA-Seq method that employed random primers in reverse transcription, and preliminary studies suggest that EP3 RNA is not polyadenylated. Its function remains to be determined.

At 96 h post-infection of D-NT2, approximately 47% of engaged Pol II on the HCMV genome is positioned at the HCMV RNA 4.9 promoter within a 40-bp promoter-proximal pause zone. The number of 5’-ends of nascent RNAs at the RNA4.9 promoter TSS (originating from this engaged Pol II) provides an estimate of the number of HCMV genomes actively transcribing in late-stage infection. Under the conditions tested, the number of HCMV genomes engaged in transcription at the RNA4.9 promoter is only 30% less than in HFF infection. Across these genomes in both cell types, the frequency of activity among other HCMV promoters spans nearly three orders of magnitude. In D-NT2, fewer LTF-driven promoters are active, and most exhibit lower activity than in HFF. However, some LTF-driven promoters remain highly active in D-NT2, at levels comparable to those in HFF.

While HCMV genomes in both cell types exhibit similar H3K4me3 histone signal levels over the RNA4.9 gene, the HCMV genome-wide nucleosome occupancy with H3K4me3 marked histones is substantially higher in D-NT2 than in HFF. This increase is at least partly attributable to elevated levels of histone H3 occupancy, as indicated by stronger H3.3 ChIP signal across the HCMV genome in D-NT2 than in HFF. Notably, H3.3 is not the only histone H3 variant that is known to occupy the HCMV genome [31]. In HFF, the DNA sequence composition upstream of LTF-activated promoters predicts promoter strength, with weaker promoters exhibiting lower LTF occupancy [15]. These weaker LTF-activated promoters may be particularly susceptible to transcriptional hinderance due to increased nucleosome occupancy in D-NT2. Notably, nearly half of the viral early-late and late promoters in D-NT2, are not driven by LTFs or IE2, unlike in HFF. The mechanisms driving transcription at these viral promoters remain unknown.

The HCMV enhancer drives MIEP transcription, leading to IE2–86 protein production, which initiates the HCMV replication cycle [32,33]. Our findings indicate that the enhancer lacks histone H3.3 and H3K4me3 and H3K27Ac signals in late-stage infections of HFF and D-NT2, suggesting that active promoters and cis-regulatory elements within the enhancer may create a nucleosome depleted region. The role of IE2 in negative autoregulation of MIEP activity via binding to the 14-bp *crs* (-3 to -17 relative to the MIEP TSS) is well established [32,33]. Our findings build on the recent discovery of a TBP-IE2-nucleosome complex at the MIEP in fibroblasts at 48 h pi [16]. Using a different HCMV strain, we now show at 96 h pi, D-NT2 contain more nucleosome within the TBP-IE2-nucleosome complex than in HFF. Despite this difference, TBP and IE2 co-occupy the MIEP core promoter in equally high amounts. The nucleosome adjacent to or overlapping the TBP-IE2 complex in D-NT2 is marked by H3K4me3 and H3K27Ac modifications. Previous studies have shown H3K27Ac signal is highly enriched at RNA4.9/Ori*Lyt*, and to a lesser extent at the MIEP, in HCMV-infected fibroblasts and Kasumi 3 cells [34,35]. Our results are consistent with prior evidence that chromatin structure and its modifiers regulate HCMV promoter activity, including the MIEP [36–40].

We propose that a TBP-IE2-nucleosome complex forms at the MIE core promoter, likely with involvement of additional factors. The MIEP TATA box is likely critical for TBP-IE2-nucleosome complex formation, with the *crs* and possibly other DNA sequences also playing essential roles. Given that viral pUL84 physically interacts with IE2 and is enriched at the MIEP based on prior CHIP-Seq data [41], we presume it is involved in this process. However, its precise role and binding location within the MIEP remain uncertain. We favor the hypothesis that TBP and IE2 cooperate to position a nucleosome as part of a larger TBP-IE2-nucleosome complex. The relative abundance of nucleosome in this complex reflects cell type differences in viral chromatin, including overall levels of nucleosome occupancy on the viral genome*.* The TBP-IE2-nucleosome complex possibly interferes with host TFIIB binding to DNA downstream of the TATA box, thereby blocking Pol II positioning and transcription initiation. Epigenetic modifications that alter the configuration or composition of the nucleosome within the TBP-IE2-nucleosome complex may determine whether the MIEP transcription block is temporary or sustained, depending on cellular condition and external stimuli.

## Materials and methods

### Cells and viruses

Cells: Human foreskin fibroblasts (HFF) were isolated from discarded human foreskins obtained per IRB approval (IRB ID#: 201702734). HFF were cultured in Minimum Essential Medium (Gibco, 11095080) supplemented with 8% heat-inactivated fetal bovine serum (FBS, Gibco, 26140079) and 1000 U/mL penicillin-streptomycin (Gibco, 15140122). NTera2/D1 clone cells (NT2), originated and provided by E. Gonczol et al. [23], were cultured in Dulbecco’s Modified Eagle Medium with glutamine (Gibco, 11965092) supplemented with 1 mM sodium pyruvate (Gibco, 11360070), 1000 U/mL penicillin-streptomycin, 4% charcoal-treated FBS and 3% Knock-Out Serum Replacement (Gibco, 10828028) and split without the use of trypsin or EDTA. Charcoal-treated FBS was prepared, by incubating 7.5 g of activated charcoal powder (Sigma Aldrich, C3345) in 500 mL FBS overnight at 4˚C on a rocker platform; after the charcoal was allowed to sediment, the charcoal-treated FBS was passed through a 0.4 µm-pore low-protein binding filter and then a 0.2 µm-pore filter. Culturing NT2 in DMEM with 10% FBS yields a sizeable proportion of cells expressing HCMV MIE protein within 24 hours of infection [24,42]. Adding 10 µM retinoic acid (Sigma Aldrich, R2625) to 10% FBS for 10 d converts the NT2 population into differentiated NT2 (D-NT2) of neural lineage [24,25,42]. Retinoic acid was removed on the day before the infection, after which the cells were cultured in DMEM with 10% FBS. The Sf21 moth cell line was grown at 27°C in Sf-900 III SFM (Gibco 12658019).

Viruses: These studies used HCMV Towne (varS) [43] and HCMV Towne containing a bacterial artificial chromosome [44], a BACmid virus. To prepare HCMV for experiments, supernatant of infected HFF was filtered (0.45-μm filter), layered on a 20% sorbitol cushion in phosphate-buffered saline (PBS), and subjected to centrifugation. The pellet of cell-free virus was resuspended in DMEM without serum. Construction and characterization of HCMV Towne BACmid viruses IE2^F^ and UL87^HF^ were previously reported by M. Li et al. [14].

Viral genome mutagenesis: HCMV Towne BACmid N1, N2, NB, and CK viruses differ only from the parent WT BACmid virus in base substitution mutations that target different cis-acting elements in the MIE enhancer. To place site-directed mutations in the enhancer, a section of enhancer from -47 to -548 was replaced by the bacterial galK gene, using homologous recombination and the GalK selection method [14,45]. The GalK insert in the enhancer-GalK virus was replaced by homologous recombination in *E. coli* SW105 using two distinct double-stranded DNA gene blocks carrying the desired mutations. One end of each gene block contained a short stretch of matching DNA sequence, while the opposite ends differed according to the homologous viral sequences flanking the GalK insert. The oligonucleotides and double-stranded DNA gene blocks used to construct N1, N2, NB, and CK viruses are listed in S4 Table. Sanger sequencing verified the desired enhancer DNA sequence, and restriction fragment length polymorphism analysis and high-throughput DNA sequencing verified the sequence integrity of the entire genome. To recover the infectious virus, HCMV Bacmid DNA was nucleofected into HFF using the Amaxa Neonatal Human Dermal Fibroblasts kit (Lonza, VPD-001) and the Amaxa Nucleofector II with program setting U23. HCMV Bacmid DNA was transformed in *E. coli* DH10B for large-scale HCMV BACmid DNA preparation using the QIAGEN Plasmid Maxi Kit (12163). All gene blocks and primers used throughout this report were purchased from Integrated DNA Technologies.

### Infections and treatments

Subconfluent D-NT2 in 100 mm cell culture dishes and confluent HFF at passage number ≤6 in T150 flasks were used in experiments. Recombinant BACmid viruses used in experiments were passage number ≤3. Viral stocks used in comparison of different viruses were titrated in parallel on confluent HFF to determine the quantity of viral particles per mL of inoculum capable of actively infecting HFF. The multiplicity of infection (MOI), the ratio of infectious viral particles per HFF cell, was determined from serial dilutions of viral inoculum applied to HFF in a 24-well plate. Immunofluorescence assay (IFA) was then performed at 24 h pi to determine percentage of HFF expressing HCMV IE1/IE2 protein. IFA was performed using a murine monoclonal antibody MAB810 (EMD Millipore, 1:1000 dilution) followed by a secondary goat anti-mouse IgG (H + L) antibody conjugated to Alexa Fluor 555 (Thermo Fisher Scientific, A-21422) and counterstained with DAPI to identify cell nuclei. An inverted Olympus IX 51 Fluorescent Microscope equipped with an X-Cite 120 Fluorescence Illumination System was used to capture images. All infection experiments involving PRO-Seq, RNA-Seq, and DFF-ChIP Seq were conducted at an MOI 2.5- to 3-fold higher for D-NT2, than the MOI applied to HFF. The viral titer (infectious units per mL) in the viral stock was determined in HFF. After viral adsorption, the cells in these experiments were washed once with growth medium or 1x phosphate-buffered saline (PBS). Favopiridol (Flavo, NIH AIDS Reagent Program 9925) dissolved in DMSO was added to culture medium at final concentration 1 μM for the final 1 h of HCMV infection. Phosphonoformic acid (PFA) (Sigma-Aldrich P6801) was added to culture medium at a final concentration of 400 μg/mL throughout the 72-h and 96-h HCMV infections. The cereblon VHL-recruiting molecule, dTag, was kindly provided by Nathanael Gray and Nabet Behnam. dTag dissolved in DMSO was added to culture medium at a final concentration 200 nM for 6 h from 90 to 96 h pi.

### Western blot

Cells were lysed in a buffer (10% glycerol, 1% Triton X-100, 20 mM Tris [pH 7.9], 137 mM NaCl, 1 mM Na_3_VO_4_, 5 mM EDTA, 1 mM EGTA, 10 mM NaF, 1 mM Na pyrophosphate, 1 mM β-glycerophosphate) containing Complete Mini, EDTA-free protease inhibitor mixture (Roche Diagnostics GmbH, Penzberg, Germany) and phenylmethlysulfonyl fluoride (0.5 mM). Cell lysates were briefly sonicated, mixed with 5x gel-loading buffer (final 25 mM Tris [pH 6.8], 2% SDS, 4% glycerol, 20 mM beta-mercaptoethanol, and 0.01% bromophenol blue), and denatured at 95°C for 5 minutes. Cell lysates were then electrophoresed in freshly made 8% SDS-Tris-glycine polyacrylamide gels and transferred to Amersham Protran 0.45-μm nitrocellulose membranes (GE Healthcare Life Sciences, 10600002) using the Thermo Scientific Owl Panther Semidry Electroblotter at 200 mA for 30 minutes. The amino-terminal end shared by IE1–72 and IE2–86 was detected using murine monoclonal antibody MAB810 (EMD Millipore, 1:1,000 dilution), whereas murine monoclonal antibody MAB8140 (Millipore Sigma, 1:1,000 dilution) was used to detect the shared regions of IE2–86, IE2–60, and IE2–40. HCMV pUL44 and pp28 were detected using mouse monoclonal CMV UL44 antibody (Fitzgerald, 1:2,000 dilution) and CMV p28 UL99 antibody (Virusys, 1:500 dilution), respectively. The HA-tagged UL87 protein was detected using monoclonal mouse HA.11 epitope tag antibody (BioLegend, 1:1,000 dilution). Actin was detected using polyclonal rabbit anti-actin antibody (Sigma-Aldrich, A2066, 1:4,000 dilution). Secondary antibodies included peroxidase AffiniPure F(ab’)2 fragment goat anti-mouse IgG (Jackson ImmunoResearch, 115-036-006, 1:40,000 dilution), rabbit anti-mouse IgG (whole molecule)–peroxidase antibody (Sigma-Aldrich, A9044, 1:40,000 dilution), or goat anti-rabbit IgG (whole molecule)–peroxidase antibody (Sigma-Aldrich, A0545, 1:40,000 dilution). Peroxidase was detected by chemiluminescence using the SuperSignal West Femto Maximum Sensitivity Substrate (ThermoFisher Scientific, 34095). The iBright FL1500 Imaging System (Invitrogen) was used to capture images of the chemiluminescence and quantify relative differences in band intensities.

### Viral DNA replication assay

HFF and D-NT2 cells were infected in triplicate with HCMV at the indicated MOI. Cells were washed three times after 90 min of virus adsorption. Infected cells were washed with 1x PBS before adding PCR lysis buffer (10 mM Tris-HCl, pH 8.0, 1 mM EDTA, 0.001% Triton X-100, 0.0001% SDS) containing 20 μg/mL proteinase K [43]. The lysate was incubated at 55°C for 100 min and then heat-inactivated at 95°C for 20 min. The Applied Biosystems 7500 Fast Real-Time PCR System was used with Power SYBR Green PCR Master Mix (ThermoFisher, 4367659) to quantify HCMV DNA with primers targeting IE1 exon 4 [14]. The quantity of amplified HCMV DNA was determined using the standard curve method and normalized to the amount of host glyceraldehyde-3-phosphate dehydrogenase (GAPDH) DNA. PCR parameters were 95°C for 10 min, followed by 40 cycles of 95°C for 15 s and 60°C for 60 s.

For comparison of HCMV DNA levels in nuclei of D-NT2 and HFF over time, D-NT2 and HFF were prepared in the manner described above and infected in duplicate with HCMV WT Towne BACmid at MOI 6 and 2, respectively. Nuclei were isolated at 24, 48, 72, and 96 h pi, according to the rapid nuclei isolation method. The number of nuclei per unit volume was calculated according to the DFF ChIP-Seq method. For qPCR, equal amounts of nuclei from D-NT2 and HFF were used for each time point, and HCMV and GAPDH DNA levels were quantified using the method described above.

### RT-qPCR analysis

Reverse-transcription followed by quantitative PCR (RT-qPCR) was applied to measure changes in levels of specific RNAs. Whole-cell RNA isolated by TRIzol Reagent (Invitrogen, 15596026) was subjected to reverse transcription using random hexamer primers (Roche, 11034731001) and SuperScript III Reverse Transcriptase (Invitrogen, 18080044), according to the manufacturer’s instructions. To measure unspliced HCMV UL99 and IE1 Exon 4 RNAs, the residual DNA in the RNA sample was removed by DNase I digestion. One microgram of RNA sample in a final volume 10 µL, containing reaction buffer and 1 µL of DNase I (Thermo Fisher, EN0521), was incubated at 37°C for 30 min. The reaction was stopped by addition of 1 µL of 50 mM EDTA, followed by incubation at 65°C for 10 min. Measurements of host gene RNA levels identified by RNA-Seq were done using DNAse-treated RNA, described in the RNA-Seq method. Primers listed in S4 Table, combined with Power SYBR Green PCR Master Mix, were used for cDNA amplification on the Applied Biosystems 7500 Fast Real-Time PCR System. The cycling conditions were 95°C for 10 min, followed by 40 cycles of 95°C for 15 s and 60°C for 60 s. The quantity of amplified cDNA was determined using the standard curve method and normalized to the amount of host GAPDH cDNA. RT-qPCR analyses were performed on triplicate infections, with each sample processed for cDNA synthesis and subjected to three PCR reactions. However, RT-qPCR analysis of RNA used for RNA-Seq was conducted on duplicate infections.

### Rapid nuclei isolation

Nuclei of uninfected and infected cells were isolated under conditions that stabilize engaged RNA polymerases with their attached nascent RNA while preserving chromatin structure [19, 46]. The cells were rinsed with ice cold PBS and scraped into 10 mL of ice-cold lysis buffer containing 20 mM HEPES (pH 7.6), 300 mM sucrose, 1% IGEPAL CA-630, 1 mM spermine, 1 mM spermidine, 1 mM EDTA, 1 mM dithiothreitol (DTT), 0.004 U/μl SUPERase-In (Invitrogen, AM2696), 0.1% isopropanol-saturated phenylmethylsulfonyl fluoride (PMSF), and cOmplete EDTA-free protease inhibitor cocktail (Roche, 11873580001). Cell lysates were layered over a 20 mL sucrose cushion and centrifuged at 22,500 x g for 5 min at 4˚C. The pelleted nuclei were resuspended in storage buffer (20 mM HEPES, pH 7.6, 5 mM magnesium acetate, 150 mM potassium acetate, 5 mM DTT, and 25% glycerol), homogenized using a 1 mL Dounce with a tight pestle, aliquoted, and stored at –80°C. 100 μL storage buffer was used per T-150 flask or 100 mm cell culture dish.

### PRO-Seq method

Each PRO-Seq library was generated from nuclei isolated from either a single 100 mm cell culture dish of subconfluent D-NT2 (MOI 5–8, based on MOI determination in HFF) or a single T-150 flask of HFF (MOI 2–3). Nuclei in storage buffer were gently pelleted, and storage buffer removed. The pelleted nuclei were re-suspended in 40 μL buffer containing 20 mM HEPES (pH 7.8), 100 mM potassium chloride, 5 mM magnesium chloride, 5 mM DTT, and 0.6 U/μL SUPERase-In (Invitrogen, AM2696). This buffer also contained moth Sf21 cell nuclei (Spodoptera frugiperda origin) as a normalization control (~100, 000 Sf21 nuclei per library). The procedure for using engaged RNA Pol to incorporate a biotinylated nucleotide at the 3’-end of nascent RNA and thereby terminate Pol elongation involved adding 20 μL of a 3X reaction mixture—20 mM HEPES (pH 7.8), 5 mM magnesium chloride, 100 mM potassium chloride, 5 mM DTT, 1.5% Sarkosyl, and 60 μM biotinylated ATP, UTP, GTP, and CTP (Perkin Elmer, NEL544, NEL543, NEL545, and NEL542)—and incubation at 37˚C for 10 minutes. The reaction was stopped by the sequential addition of 40 μL of 50 mM EDTA and 300 μL TRIzol LS (Ambion, 10296028). After Trizol RNA extraction according to the manufacturer’s protocol, the RNA pellet (washed with 70% ethanol) was resuspended in 20 μL RNase-free H2O, incubated at 65˚C for 2 min, and then chilled on ice for subsequent RNA fragmentation by base hydrolysis. This entailed adding 5 μL 1 N NaOH, incubating the mixture on ice for 20 minutes, and stopping the RNA hydrolysis reaction with 25 μL 1 M Tris (pH 7.8). To isolate the biotinylated RNA, 1 μL SUPERase-In and 50 μL of washed Dynabeads Protein G (Invitrogen, 100.03D) were added to the 50 μL sample of fragmented RNA and incubated at room temperature (RT) for 15 min with rotation. The beads were washed three times with 500 μL M-280 high-salt wash buffer and twice with 500 μL M-280 low-salt wash buffer. The RNA was extracted from the beads using 300 μL TRIzol LS, 2 μL glycogen, and 81 μL chloroform, and the RNA precipitated with 95% ethanol and 500 mM ammonium acetate followed by washing the RNA pellet with 70% ethanol. An RNA adapter VRA3-8N – unique molecular identifier (UMI) composed of 8 random nucleotides [14] – was ligated to the 3’ end of the RNAs after first dissolving the RNA pellet in 8 μL 12.5 μM RNA adapter VRA3-8N and heating to 65˚C for 2 minutes followed by snap-cooling on ice. To this, 12 μL 5/3X Rnl1 mix (1.67 U/μL T4 RNA ligase 1 ssRNA (NEB, M0204), 5/3X ligase reaction buffer, 1.67 mM ATP, 25% PEG 8000, and 1.67 U/μL SUPERase-In) was added and incubated at 37˚C for 4 h. Ligated samples were then mixed 30 µl RNase-free H_2_O and 50 µl washed Dynabeads at RT for 15 min with rotation. Beads were washed and RNA extracted using the same procedures noted above, except glycogen was not added. To remove the 5’ cap from the RNA, the RNA pellet was resuspended in 10 µl RNase-free H_2_O, incubated at 65°C for 2 min, and snap-cooled on ice. To this, 10 μL 2X RppH mix (0.5 U/μL RNA 5’ pyrophosphohydrolase (NEB, M0356), 2X ThermoPol buffer, and 1 U/μL SUPERase-In) was added and incubated at 37˚C for 1 h. The 5’ end of the RNA was then phosphorylated by addition of 80 μL 5/4X T4 PNK mix (0.313 U/μL T4 PNK (NEB, M0201), 5/4X T4 PNK buffer, 1.25 mM ATP, and 0.625 U/μL SUPERase-In) and incubation at 37˚C for 1 h. RNA was isolated with TRIzol LS and precipitated, and pellets were resuspended in 8 µl 12.5 µM RNA adapter VRA5-8N (UMI sequence composed of 8 random nucleotides [14]), incubated at 65°C for 2 min, snap-cooled on ice, and incubated at 37°C for 4 h with 12 µl 5/3 × Rnl1 mix. To this sample, 30 µl RNase-free H_2_O and 50 µl washed Dynabeads were added and incubated at RT for 15 min with rotation. Beads were again washed and RNA extracted using the same procedures noted above.

The adapter ligated RNA was reverse transcribed with SuperScript IV (Thermofisher 18090010) in total volume 20 µL. This was performed using 5 μM RP1 primer, 1 mM deoxynucleoside triphosphate [dNTP] mix [NEB N0447], 2X SuperScript IV buffer, 10 mM DTT, 2 U/μL SUPERase-In, and 20 U/ μL SuperScript IV enzyme incubated at 45°C for 15 min, 50°C for 40 min, 55°C for 10 min, and 70°C for 15 min. To compare cDNA concentrations between samples, 3 µL of RNase-free H₂O was added to each sample (totaling 23 µL). Then, 2 µL was removed and subjected to four stepwise 1:4 dilutions in RNase-free H₂O. The cDNA amount in 2 µL of each of four stepwise dilutions after 20 cycles inversely corresponds to PCR cycle differences of 16, 14, 12, and 10, respectively. The PCR reaction was performed in final volume of 25 µL containing 1.5 μM RP1, 1.5 μM Illumina forward index primer RPI-6, and 12.5 μL KAPA HiFi HotStart ReadyMix (KAPA Biosystems, KK2601) and following PCR parameters: 98°C for 45 s; 20 cycles of 98°C for 15 s, 60°C for 30 s, and 72°C for 30 s; followed by 72°C for 1 minute and a hold at 4°C. PCR products were analyzed on TAE 6% acrylamide gels. Given the consistent product yield across library constructions, all PRO-Seq libraries in S1 Table were subjected to 13 cycles of amplification using the following PCR parameters: 98°C for 45 seconds; 13 cycles of 98°C for 15 s, 60°C for 30 s, and 72°C for 30 s; followed by 72°C for 1 min and holding at 4°C. This PCR was performed in a volume totaling 50 μL and containing the remaining 21 μL of cDNA sample, 1.5 μM of an Illumina barcoded index primer I-N (N, a unique bar code for each library), 1.5 μM RP1, and 25 μL KAPA HiFi HotStart ReadyMix. PCR products were purified using a Qiagen MinElute kit in a 30 μL volume. The Qubit high-sensitivity dsDNA assay was used to determine the concentration of the purified cDNA library. For cDNA size distribution analysis, 1 μL of each cDNA library was diluted to 2.5 ng/μL and analyzed using the Agilent Bioanalyzer 2100. Equimolar quantities of libraries were pooled and DNA fragments between 145–600 bp in length were selected on a Sage Science Blue Pippen instrument with a 2% agarose gel cassette (BDF2010) and Marker V1 internal standards. The Qiagen MinElute kit was used to purify the size-selected DNA, and the Qubit Fluorometer used to measure DNA concentration. The size-selected DNA library pools were diluted to 2.5 ng/μL for re-analysis on the Agilent Bioanalyzer. Once correct size-selection was confirmed, the PRO-Seq libraries were sequenced at the Iowa Institute of Human Genetics using the Illumina NovaSeq 6000 System and SP flow cells to generate 50-bp paired-end reads for Experiment 1 and 100-bp paired-end reads for Experiments 2–6 (S1 Table).

### DFF-ChIP Seq method

Human DNA Fragmentation Factor (DFF) produced from a bacterial expression plasmid was purified, and its DNA endonuclease activity was assessed following the procedures reported by Spector et al. [21]. Both native and cross-linked DFF-ChIP Seq was applied to nuclei rapidly isolated using the method detailed above. Cells were cross-linked prior to nuclei isolation by adding paraformaldehyde (Electron Microscopy Sciences, 15710) at a final concentration of 1% for 10 minutes at room temperature. The crosslinking was stopped by adding Tris (pH 7.6) at a final concentration of 1.33 M and followed by the rapid nuclei isolation procedure. To compare results of multiple ChIP antibodies within an experimental group, more than a single dish or flask of cells was needed to obtain enough nuclei. Nuclei from each experimental group were pooled, and three small aliquots were set aside for DNA quantification prior to storage at -80˚C. The DFF amount needed to digest the chromatin required knowing the number of nuclei per unit volume. The number of nuclei per unit volume for each nuclei stock was calculated based on the assumption of 7 pg of DNA per nucleus. DNA was isolated from the three small aliquots of nuclei using the following method. Each of these aliquots was brought up to final 200 μL with buffer containing 20 mM HEPES (pH 7.6), 100 mM potassium acetate, 5 mM magnesium acetate, and 5 mM DTT. Each aliquot was then sonicated for 6 cycles of 20 s-on and 40 s-off in a Qsonica Q800R3 Sonicator at 40% amplitude. RNAse A (0.1 μg/μL final concentration, Thermo-Scientific, EN0531) was added for 30 minutes at 37°C followed by addition of proteinase K (0.2 μg/μL final concentration, Thermo-Scientific, EO0491) for 2 h at 55°C. The soluble DNA was extracted with an equal volume of phenol chloroform (1:1) and then precipitated by adding 3 volumes of 95% ethanol containing 0.5 M ammonium acetate. The DNA pellet was washed with 70% ethanol and resuspended in 30 μL of water. The DNA concentration in each aliquot was measured using a NanoDrop spectrophotometer, and the average concentration for the three aliquots determined.

Just before performing the DFF-ChIP procedure, pooled nuclei in storage buffer were gently pelleted in a Prism Mini Centrifuge for 10–15 s, and the storage buffer was discarded. Five million nuclei were used for DFF digestion of native and cross-linked chromatin in nuclei. After digestion, nuclei were pooled again before aliquoting 5 million nuclei for each ChIP. Nuclei were digested with 15 μg DFF in a final volume of 100 µL containing 20 mM HEPES (pH 7.6), 5 mM magnesium acetate, 100 mM potassium acetate, and 5 mM DTT. Native and cross-linked chromatin was digested at 37 °C for 30 and 45 min, respectively. The digestion was stopped by adding 2.5 µL of 0.5 M EDTA. To isolate fragmented chromatin, nuclei were lightly sonicated for 20 s using a Qsonica Q800R3 Sonicator at 40% amplitude, then centrifuged at 13000 rpm for 10 min at 4°C in an Eppendorf 5415R refrigerated centrifuge. The supernatant was collected and adjusted to a final volume of 1 mL with a solution containing 10 mM Tris (pH 7.5), 100 mM sodium chloride, 1 mM EDTA, and 0.1% TritonX-100. To reduce non-specific protein binding, 40 µL of Protein A/G PLUS-agarose beads (Santa Cruz, sc-2003) were added for 30 min to pre-clear the supernatant. The beads were then removed, and primary antibodies were added to solution mixture, followed by overnight incubation at 4°C with rotation on the HulaMixer sample mixer (Thermo Fisher Scientific) (Settings: orbital: 20 02; reciprocal: 90˚ 07; vibro/pause: 1˚ 1). To capture antibody-protein-DNA complexes, 40 µL of Protein A/G PLUS-agarose beads were added and incubated for 2 h at 4°C with rotation on the HulaMixer. The beads were washed five times with 10 mM Tris (pH 7.5), 1 mM EDTA, 150 mM sodium chloride, and 0.1% TritonX-100 for 5 min per wash. To elute the bound material, 50 μL of 10 mM Tris (pH 7.5), 1% SDS, and 1 mM EDTA was added and incubated at 65°C for 5 min, and this was repeated. The eluted material was treated with 20 μg RNAse A (Thermo-Scientific, EN0531) for 30 min at 37°C followed by addition of 40 μg of proteinase K (Thermo-Scientific, EO0491) for 2 h at 50°C. Phenol-chloroform was used to extract the DNA, and the soluble DNA was precipitated using 3 volumes of 95% ethanol containing 0.5 M ammonium acetate. The DNA pellet was washed with 70% ethanol and resuspended in RNAse/DNase-free water for library preparation.

The ChIP experiments used antibodies against Pol II (3 μg, Santa Cruz, sc-55492), H3K4me3 (3 μg, Abcam, ab8580), TBP (3 μg, Abcam, ab51841), H3K27Ac (5 μg, Cell Signaling, D5E4), H3.3 (4 μg, Active Motif, 91191), and HCMV IE2 (10 μg, Millipore Sigma, MAB8140).

All libraries were prepared using the NEBNext UltraII DNA Library Prep Kit for Illumina (NEB#7645S). Construction of libraries for Experiments 1, 2, and 3 (S2 Table) included use of custom adapters with an 8 bp UMI, as described by Spector et al. [21]. In Experiment 4 and 5 (S2 Table), we used NEBNext Multiplex Oligos for Illumina, which include 12 bp UMIs attached to the 8 bp i7 index (Unique Dual Index UMI Adaptors DNA Set 1, NEB#E7395S). Test PCR amplifications were performed on each library using the stepwise dilution approach and TAE 6% acrylamide gel analysis, as described for PRO-Seq. This process determined the number of cycles required to achieve comparable DNA quantities across libraries for subsequent sequencing. Fourteen PCR cycles were applied to Pol II and IE2 ChIPs, 13 cycles for TBP ChIP, and 5 cycles for H3K4me3 and H3K27Ac ChIPs. After library amplification, the DNA was purified and quantified using methods applied to PRO-Seq libraries. Equimolar quantities of libraries were pooled and DNA fragments between 145–850 bp (Experiments 1–3) and 164–850 bp (Experiments 4 and 5) in lengths were selected using the BluePippin. The selected DNA fragments were purified and analyzed as described for PRO-Seq libraries. Libraries were sequenced at the Iowa Institute of Human Genetics on the Illumina NovaSeq 6000 System using SP flow cells, generating 50-bp paired-end reads for Experiments 1–4. Experiment 5 was sequenced on the Element Aviti24, using a Cloudbreak Freestyle sequencing kit with a high output capacity and generating 75-bp paired-end reads.

### RNA-Seq method

Total RNA was isolated from biological duplicates of D-NT2, either uninfected or infected in parallel with HCMV Towne WT, N1, NB, and CK viruses for 96 h. TRIzol Reagent (Invitrogen, 15596026) was used according to the manufacturer’s instructions to generate RNA pellets, which were resuspended in 50 μL of DNase digestion buffer containing 2 U Turbo DNase (Invitrogen, AM2238) and 20 U SUPERase-In RNase inhibitor. After incubation at 37°C for 30 min, 300 μL TRIzol was added to stop the DNA digestion. The RNAs were further purified using the Zymo Direct-zol RNA Miniprep kit (Zymo, R0250). RNA concentration and integrity (RIN score >9) were determined using a NanoDrop spectrophotometer and the Agilent Bioanalyzer 2100, respectively. Lunatic spectrophotometer measurements confirmed the absence of significant DNA or other organic contaminants. Total RNA-Seq libraries were prepared using the Illumina TruSeq-Stranded Total RNA Prep kit with Ribo-Zero for Human/Mouse/Rat (Illumina, 200020596), according to manufacturer’s instructions. Briefly, 500 ng total RNA was resuspended in 10 μL of nuclease-free water in individual 0.2 mL PCR tubes. Five microliters of each ribosomal RNA (rRNA) removal mix and rRNA binding buffer were added, followed by RNA denaturation at 68°C for 5 min and transfer of the sample to a 1.5 mL Eppendorf tube. To deplete the rRNA, 35 μL of rRNA removal beads were added to the sample, mixed thoroughly, and incubated at room temperature for 1 min, and captured on a magnetic stand (EpiCypher, 10–0008) for 1 min. The supernatant was transferred to a new 1.5 mL Eppendorf tube. The rRNA-depleted RNA was then captured on 99 μL of RNAClean XP beads (Beckman Coulter, A63987) and the magnetic stand, followed by ethanol washes. The rRNA was eluted in 11 μL elution buffer. To fragment the rRNA-depleted RNA, 8.5 μL of the eluted sample was transferred to a 0.2 mL PCR tube, combined with 8.5 μL elution-prime-fragmentation mix, and incubated at 94°C for 8 min. First strand cDNA was synthesized using 8 μL first strand superscript mix (Superscript II: First Strand reagent = 1:9) containing random hexamers, with the following conditions: 25°C for 10 min, 42°C for 15 min, 70°C for 15 min, and hold at 4°C. Second strand cDNA was synthesized using 20 μL second strand marking mix and 5 μL resuspension buffer. The double-stranded cDNA was purified using AMPure XP beads (Beckman Coulter, A63880). After adenylation of 3′ ends and adapter ligation, PCR amplification was performed under the following cycle parameters: 1 cycle of 98°C for 30 s; 15 cycles of 98°C for 10 s, 60°C for 30 s, 72°C for 30 s; 1 cycle of 72°C for 5 min; and hold at 4°C. The PCR products were purified using AMPure XP beads, and the DNA concentration measured using the Qubit high-sensitivity dsDNA assay. Library quality was assessed on the Agilent Bioanalyzer 2100 system, after diluting 1 μL of each DNA library to 2.5 ng/μL. Once deemed acceptable, all samples were diluted to 3 ng/μL and pooled in equimolar amounts. Sequencing was performed at the Iowa Institute of Human Genetics on the Illumina NovaSeq 6000 System using SP flow cells, generating 50-bp paired-end reads.

### Processing of PRO-Seq, DFF-ChIP Seq and RNA-Seq datasets

PRO-Seq FASTQ files from paired-end sequencing datasets were processed using methods previously reported [15,28], with some modifications. Libraries were prepared using custom made random 8N UMI RNA adapters at both ends of the nascent RNA fragments [14]. The 3’ sequencing adapter sequences were trimmed using trim_galore v0.6 (https://github.com/FelixKrueger/TrimGalore) with a minimum read length of 18. The reads were then aligned to three concatenated reference genomes - HCMV Towne sequence (GenBank accession number FJ616285.1), human genome sequence (UCSC assembly hg38), and moth Sf21 genome sequence (WGS number JQCY02), using Bowtie v1.2.3 [47], with -trim5 8 and -trim3 8 to account for the 8N UMIs. The concatenation was adjusted to account for mutations introduced into HCMV Towne FJ616285.1 genomes – genome concatemers for Towne WT, hg38, and JQCY02.1; Towne N1, hg38, and JQCY02.1; Towne N2, hg38, and JQCY02.1; Towne NB, hg38, and JQCY02.1; and Towne CK, hg38, and JQCY02.1. Output.sam files were then deduplicated and the biotinylated nucleotide removed from the 3’ end as described in (https://github.com/P-TEFb/dedup) [9] to generate de-duplicated bed files. The bedGraph files were converted to bigWigs using bedtools genome coverage [48] and bedGraphToBigWig [49]. Datasets with spike-in controls (moth Sf21) were normalized, as described in Ball et al. [50]. Other datasets were normalized to total number of mapped reads. Flavo and vehicle control (No Flavo) groups were normalized separately. Normalization factors were applied to genome coverage values in bedGraph files and included in the mapping statistics available in S1 Table. Strand-specific 5’ tracks were generated using bedtools genome coverage and bedGraphToBigWig. The PRO-Seq data was visualized on track hubs in the UCSC Genome Browser.

For DFF-ChIP Seq, we utilized a custom Snakemake pipeline (https://doi.org/10.5281/zenodo.10079297) to convert fastq files into bigwig format. Initially, adapter sequences were trimmed from the fastq files using trim_galore v0.6. Trimming was conducted with a length parameter of 18, using adapter 1 sequence AGATCGGAAGAGCACACGTCTGAACTCCAGTCA and adapter 2 sequence AGATCGGAAGAGCGTCGTGTAGGGAAAGAGTGT. Sequences were aligned using Bowtie v1.2.3. Processed reads were aligned to concatenated human hg38 and HCMV Towne FJ616285.1 genomes. Eight nucleotide UMIs present in the index reads (R2) for Experiments 1–3, and 12 nucleotide UMIs present in the index reads (R2) for Experiments 4 and 5 were used for deduplication (https://doi.org/10.5281/zenodo.10041794). Mapping statistics are provided in S2 Table. After deduplication, the files were converted into bedGraphs using bedtools genome coverage and bigWig files were generated with bedGraphToBigWig. (https://github.com/ENCODE-DCC/kentUtils/tree/master/bin/linux.x86_64).

RNA-Seq reads were trimmed of Illumina adapters using trim_galore v0.6. The trimmed reads were mapped to two concatenated reference genomes – human hg38 and HCMV Towne FJ616285.1 – using the stranded/splicing-aware aligner HISAT2 (http://daehwankimlab.github.io/hisat2/manual/). The concatenation was adjusted to account for mutations introduced into HCMV Towne FJ616285.1 genomes – genome concatemers for Towne WT and hg38; Towne N1 and hg38; Towne NB and hg38; and Towne CK and hg38. Alignment files were subsequently converted to bedGraphs using bedtools genome coverage, and bigwig format files were generated with bedGraphToBigWig. Read mapping statistics are provided in S3 Table. Changes in host gene expression were assessed using DESeq2, and the visualization was performed using EnhancedVolcano in R.

### Analysis of differences in viral TSS strength in HFF vs D-NT2

The tsrFinderM1 tool (https://github.com/P-TEFb/tsrFinderM1) was used with default parameters to identify all strand specific positions having a clusters of 5′-end read density that met pre-specific criteria of a transcription start region (TSR) [15]. The position within each TSR window with the highest number of 5’-end reads was designated as the MAXTSS, representing TSS strength. These parameters were applied to PRO-Seq data for HFF and D-NT2 infected with HCMV (WT Towne) for 96 h and treated with Flavo for the final hour (Exp 5, S1 Table) to determine MAXTSS positions and number of MAXTSS reads at each site. The resulting 5’-end counts were adjusted by multiplying them with their sample-associated spike-in correction factors. The strengths of viral MAXTSSs were then normalized based on the same total viral reads, excluding reads from the RNA4.9 region. A union of the genomic intervals of MAXTSSs was generated from the HFF and D-NT2 datasets, retaining 2,464 MAXTSSs with a strength of at least 200 reads or higher in at least one dataset for further analysis. Notably, the high sequencing depth, which yielded up to 42 million reads mapping to the HCMV genome, increased the number of TSRs with a MAXTSS exceeding 200 reads. Differentially expressed MAXTSSs were identified using the NOISeq program [51] in R was used with upper quartile normalization and no replicates (https://www.bioconductor.org/packages/release/bioc/html/NOISeq.html). 124 MAXTSSs with significance <0.005 were displayed on a volcano plot to indicate their estimated probability of differential expression. The volcano plot was generated using the log-fold change (M), and the prob columns from the NOISeq output file. The plot displays the viral TSSs with highly significant strength differences between HFF and D-NT2 datasets that surpass an estimate probability of 0.995 (1-probability value). Similarly, PRO-Seq datasets for HFF (GSE139114) and D-NT2 (Exp 2, S1 Table) were adjusted to achieve equivalence in total viral reads across the genome, excluding reads from the RNA4.9 region, and analyzed using the same method. The plot was created using Python’s matplotlib and the color gradients generated using the matplotlib colormaps Blues and Reds.

### Analysis of differences in viral TSS strength in D-NT2 treated with dTag or PFA

The tsrFinderM1 tool was applied to PRO-Seq with Flavo datasets to call strand-specific TSRs based on default parameters. The position within the TSR window with the highest number of 5’ end reads was designated as the MAXTSS. The strength of a MAXTSS or TSR was assigned as the total number of strand-specific 5’ ends at the MAXTSS or TSR genomic position, respectively. The strength of every MAXTSS or TRS was then adjusted by multiplying it with its sample-associated spike-in correction factor.

The effect of IE2^F^ depletion on viral TSS strength was assessed by comparing the MAXTSS strength after dTag treatment (IE2^F^ Flavo dTAG) (Exp 3, S1 Table) with the MAXTSS strength without dTag treatment (IE2^F^ Flavo) (Exp 3, S1 Table). Similarly, the effect of pUL87^HF^ depletion on viral TSS strength was evaluated by comparing the MAXTSS strength post-dTag treatment (UL87^HF^ Flavo dTAG, Exp 6, S1 Table) with that of untreated infections (UL87^HF^ Flavo) (Exp 6, S1 Table). These datasets were spike-in normalized prior to running the tsrFinderM1. For S4C Fig (Exp 4, S1 Table) and S5C Fig (Exp 3, S1 Table), total reads normalization was applied. The top 500 strongest viral MAXTSSs were identified from the control (CTRL, No dTag) datasets, and their strengths were determined from the methods described above.

The extent to which PFA treatment affects the strength of a viral TSS is represented by the PFA Sensitivity Index (PSI), defined as the ratio of viral TSS strength without PFA treatment to its strength with PFA treatment. PSI determinations were performed for the top 500 most active viral TSSs in D-NT2 infected with HCMV (Towne) for 72 h in the absence of PFA, using previously reported methods [14,15]. This analysis involved quantifying 5’ end reads at each viral TSR, using the 72-h pi Flavo and 72 h-pi Flavo PFA datasets (Exp 1, S1 Table), and computing the ratio of viral TSR strength without PFA (72-h pi Flavo) to its strength with PFA (72-h pi Flavo PFA). The same approach was applied to determine PSIs for viral TSRs in D-NT2 infected for 96 h with and without PFA by analyzing the 96-h pi Flavo and 96-h pi Flavo PFA datasets (Exp 4, S1 Table). To prevent division by zero errors, TSRs with zero reads in the 72-h pi Flavo PFA and 96-h pi Flavo PFA datasets were assigned a value of one. PSIs for viral TSRs in HCMV (Towne)-infected HFF at 72 h pi were previously reported by Li et al. [15].

### FragMaps

We used fragMap.py to create two-dimensional (2D) heatmaps that display the average distribution and position of DFF-ChiP Seq fragments. The source code for fragMap.py is available at https://github.com/JuanFSantana/fragMap-v2. In general, fragMaps were centered on the MAXTSSs with proper strand orientation. For each fragment size spanning from 18 to 400 bp, data was generated by counting the coverage at each base across the designated genomic interval generated only from fragments of the indicated length. The aspect ratio, number of pixels, intensities assigned, and the shape of major and minor tick marks were controlled. The desired aspect ratio was implemented by choosing a discrete number of pixels for each base or fragment size. Values at each horizontal position of the fragment sizes were used to assign intensities, with 0 being white and the maximum being black. A linear relationship between relative read value and intensity was utilized, with black set at the maximum read value for most frag maps. To correct for human inaccuracies in perceiving dark and light patterns on heatmaps, a gamma correction of 0.5 was applied to all fragMaps.

### Fragment distribution plots

Fragment size frequencies were determined by counting the occurrences of individual fragment sizes or size ranges within each sample. Bash and awk scripts were used to generate these counts, which were then sorted in ascending order. Separate analyses were conducted for fragment size frequencies mapping to the host and viral genomes. Microsoft Excel was used to visualize both relative and absolute fragment size distributions for each sample, employing the scatter plot with straight lines option.

### Feature analysis

To determine the ratio of free TBP to TBP-nucleosome complexes at the MIEP, we quantified fragments corresponding to free TBP, TBP-nucleosome complex 1, and TBP-nucleosome complex 2. This involved counting fragments of specific lengths, centered within designated genomic intervals using Bedtools v2.26 intersect program, and simple awk commands for each MAXTSS in the HMCV Towne genomes. The analysis focused on 40–80 bp fragments with centers between -80 and +40 (free TBP), 140–220 bp fragments with centers between -80 and +160 (TBP-Nucleosome complex 1), and 265–360 bp fragments with centers between -80 and +300 (TBP-Nucleosome complex 2).

## Supporting information

S1 Fig HCMV DNA replication and viral transcription start-site usage in HFF vs D-NT2. (**A**) D-NT2 (MOI 6.0, determined in HFF) and HFF (MOI 2.0) were infected in parallel with HCMV. Nuclei from duplicate infections were prepared at 24, 48, 72, and 96 h pi, using the method applied in PRO-Seq. Mean and range of results of HCMV DNA levels, measured by qPCR and normalized to human GAPDH DNA levels, are shown relative the HCMV DNA level in HFF nuclei at 24 h pi. (**B**) HFF and D-NT2 were infected in separate experiments for 96 h, with Flavo added during the final hour to assess viral TSS usage. PRO-Seq was performed for nascent RNA quantification and sequence analysis, and viral TSS strength was determined for each active viral promoter. PRO-Seq datasets for HFF (GSE139114) and D-NT2 (Exp 2, S1 Table) were normalized for total viral reads, excluding reads from the RNA4.9 region, to allow comparison of transcription across the HCMV genome in HFF vs D-NT2. Differential analysis identified 97 viral promoters with ≥4-fold differences in TSS strength and their estimated probability of differential expression ≥0.995 (1 minus the p-value ≤0.005), represented as blue or red dots. Darker shades indicate overlapping data points. Six viral TSSs (UL5, UL72, EP3, UL57-AS, US16-AS, and US30-S promoters) that are ≥ 15-fold more active in D-NT2 than in HFF are marked with large dark red dots and selected for further analyses. Differences in experimental conditions and depth of sequencing between the two groups resulted in fewer evaluable viral TSSs with normalized TSS strength >200 reads (see Methods). Consequently, fewer viral promoters met the ≥ 4-fold difference threshold compared to the analysis in Fig 1D. Nonetheless, 89% of the viral promoters identified here were concordant with those shown in Fig 1D
(TIFF)

S2 Fig Additional active HCMV long promoters in D-NT2 at 96 h pi. PRO-Seq was carried out at 96 h pi of HFF (GSE139114) and D-NT2 (Exp 2, S1 Table) with CTRL or Flavo, as detailed in Fig 2 legend. Auto-scaled UCSC genome Browser views of spike-in normalized nascent RNA reads aligned to the annotated HCMV Towne genome (FJ616285.1) show HCMV long promoters for UL57-antisense (UL57-AS) (**A**), US16-AS (**B**), and US30-sense nascent RNA arising internal to US30 ORF (US30-S) (**C**).
(TIFF)

S3 Fig Additional viral long promoters relying on viral DNA replication for their activation. D-NT2 were infected for 12 h, 72 h, and 72 h in the presence of PFA inhibitor of HCMV DNA replication, as detailed in the Fig 3 legend (Exp 1, S1 Table). Auto-scaled spike-in normalized UCSC genome Browser views of nascent RNA reads aligned to the annotated HCMV Towne genome (FJ616285.1) at viral long promoters for UL57-AS (**A**), US16-AS (**B**), and US30-S (**C**) nascent RNAs. Dark red arrowheads point to TSSs of viral long promoters. Abbreviation: phosphonoformic acid, PFA.
(TIFF)

S4 Fig Independent experiment confirming that viral IE2 depletion in late infection does not affect viral long promoter transcription. D-NT2 were infected for 96 h with HCMV IE2^F^ and treated with dTag degrader vs CTRL for the last 6 h of infection, as detailed in Fig 5 legend. (**A**) Western blot analysis shows that the dTag degrader decreased IE2^F^-86 amount by 88%, compared to CTRL and normalized to the host actin control. In contrast, levels of viral IE1 had not changed. (**B, C**) Spike-in normalized nascent RNAs generated by PRO-Seq coupled with Flavo were aligned to the annotated HCMV Towne genome (FJ616285.1) (Exp 4, S1 Table). (**B**) UCSC genome Browser views of the effects of dTag vs CTRL on viral UL83 and UL84 promoters. Vertical scales were set to view dTag treatment effects on amount of nascent RNA reads produced by UL83 and UL84 promoters. Green arrowhead points to position of IE2-driven UL83 promoter. (**C**) Effect of IE2^F^ depletion on strength of each of the top 500 viral TSSs minus the RNA4.9 promoter region (any promoter) was plotted against the strength of each viral TSS in absence of dTag degrader. Viral TSSs with strengths that increase ≥2-fold or decrease by ≥50% by IE2 depletion (demarcated by hatched lines) are deemed as IE2-responsive and marked by green dots, whereas viral long promoters are marked by red dots.
(TIFF)

S5 Fig Independent experiment showing depletion of viral UL87 LTF in late infection does not affect viral long promoter transcription. (**A**) Western blot assessment of change in FKBP12^F36V^-tagged UL87 LTF (pUL87^HF^) amount in HCMV-infected D-NT2 after exposed to the dTag degrader vs vehicle control (CTRL) from 90-96 h pi, compared to levels of viral IE1 and IE2, and host actin. (**B, C**) In parallel studies, spike-in controlled PRO-Seq was applied to quantify change in viral promoter strength 1 h after Flavo was added to the infected cells (Exp 3, S1 Table). (**B**) UCSC genome Browser view of results for the MIE gene and promoter/enhancer regions. Gold arrowheads point to positions of UL87 LTF-driven UL124 and EP2 promoters. (**C**) Effect of pUL87^HF^ LTF depletion on viral TSS strength plotted vs viral TSS strength in absence of dTag degrader for the top 500 viral TSSs (any promoter). Gold and dark red dots mark LTF-driven promoters and long promoters, respectively. Arrowheads point to MIEP, EP2, and EP3, as well as UL83 and UL124 promoters.
(TIFF)

S6 Fig Major nucleotide changes in proximal MIE enhancer GC boxes do not affect HCMV MIE gene expression and replication in HFF. (**A**) Annotated section of HCMV Towne genome (FJ616285.1) showing the MIEP, enhancer, cis-repression sequence (*crs*), and the UL122 and UL123 open reading frames encoding IE2–86 and IE1–72, respectively. (**B, C**) Schematic representation of site-directed mutations (SDMs) introduced into the N1, N2, and NB GC-boxes in the proximal MIE enhancer (B) and the electrophoretic pattern of EcoRI fragments (**C**) from HCMV genomes carrying N1, N2, and NB SDMs, compared to the WT HCMV genome. Blue and red asterisks indicate WT and mutated fragments, respectively. (**D-F**) Comparison of viruses at MOI 1.0 in HFF at 24 h pi includes levels of spliced MIE RNA (**D**) and levels of IE1–72 and IE2–86 proteins relative to host actin (**E**). HCMV DNA levels at MOI 0.05 were assessed at 24, 48, 72, and 96 h pi, expressed relative to WT DNA at 24 h pi (**F**). Viral RNA and DNA from triplicate infections were quantified by PCR and normalized to host GAPDH RNA and DNA, respectively (**D, F**). WT, N1, N2, and NB DNA levels at 24 h pi were 1.00, 1.07, 1.05, and 0.96, respectively.
(TIFF)

S7 Fig Differences in HCMVs with one (N1 or N2) and two (NB) GC-box mutations. (**A**) D-NT2 were inoculated with equal amounts (MOI 3) of WT, N1, N2, and NB viruses, and viral DNA levels were measured by qPCR at 24, 48, 72, and 96 h pi. Relative DNA levels at 24 h pi was 1.00, 0.94, 0.96, and 1.02 for WT, N1, N2, and NB, respectively. (**B**) HFF (MOI 3) and D-NT2 (MOI 5) were infected in parallel with WT, N1, N2, and NB viruses for 96 h pi. Cell morphology and viral GFP fluorescence were visualized using bright-field and fluorescence microscopy (original magnification, 10x). (**C**) D-NT2 infected with WT, N1, N2, and NB viruses (MOI 5) in panel B were also subjected to PRO-Seq coupled with Flavo (Exp 2, S1 Table). Spike-in normalized reads from N1, N2, and NB infections were aligned to HCMV Towne genomes (FJ616285.1) incorporating N1, N2, and NB mutations, respectively. UCSC Genome Browser views depict the HCMV region containing MIEP and the enhancer, with scales set to facilitate comparison of MIEP and EP3 nascent RNA levels.
(TIFF)

S8 Fig Mutations in CREB (CRE) and NF-kB (kB) response elements greatly reduce EP3 transcription initiation without affecting HCMV spliced IE1 and IE2 RNA expression or viral DNA replication. (**A**) Annotated HCMV genome region showing the MIEP, enhancer, cis-repression sequence (*crs*) and UL122 and UL123 open reading frames for IE2–86 and IE1–72, respectively. (**B, C**) Schematic of mutations in CRE and kB mutations (CK virus) (**B**), and of EcoRI fragment electrophoretic pattern (**C**) comparing mutated CK genomes with WT and GalK intermediate genomes. Blue and red asterisks denote WT and mutated fragments, respectively. (**D-E**) Comparative analysis of WT and CK viruses at MOI 0.05, assessing spliced IE1 and IE2 RNA expression at 24 and 96 h pi (**D**) and viral DNA produced at 24, 48, 72, and 96 h pi, relative to WT DNA at 24 h (**E**). (**F**) HCMV DNA levels in D-NT2 infected at MOI 3 were assessed at 24, 48, 72, and 96 h pi. Viral RNA and DNA from triplicate infections were quantified by PCR and normalized to host GAPDH RNA and DNA, respectively. (**G**) UCSC Genome Browser view of spike-in normalized nascent RNA reads aligned to the HCMV MIE gene regulatory region (FJ616285.1). PRO-Seq coupled to Flavo was used to measure MIEP and EP3 nascent RNA levels, with scales adjusted to enable direct comparisons (Exp 4, S1 Table). Corresponding tracks show the base positions of 5’-ends of nascent RNAs at viral TSSs. Inset (**G**) displays the ratio of CK/WT TSS strength for MIEP, EP1, EP2, and EP3 promoters.
(TIFF)

S9 Fig D-NT2 suppress less-active but not highly active LTF-dependent viral promoters. HFF and D-NT2 were infected for 96 h in parallel with HCMV, and PRO-Seq coupled with Flavo was used to quantify 5′-ends of viral nascent RNA reads for the top 500 most active viral TSSs (Exp 5, S1 Table). Total viral reads were normalized between HFF and D-NT2 infections. Graphs display viral TSS strengths (number of reads) for LTF-dependent and LTF-independent viral promoters, ranked by promoter strength in HFF (orange red) and D-NT2 (blue) infections.
(TIFF)

S10 Fig Paraformaldehyde-crosslinking does not prevent the increased H3K4me3 histone occupancy of nucleosome-sized DNA segments of HCMV genomes in D-NT2. D-NT2 were infected with HCMV for 96 h and treated with or without paraformaldehyde crosslinking before DFF-ChIP for H3K4me3 (Exp 4, S2 Table). DNA fragment reads were aligned to the host (human hg38) and HCMV Towne (FJ616285.1) genomes. The graph compares the number of reads and fragment sizes (18–500 bp) for native (solid lines) and crosslinked (hatched lines) DNA fragments from the host genome (blue) and HCMV genome (red). Total viral reads were normalized to account for a 27% difference in GAPDH-normalized viral DNA quantities between native and crosslinked D-NT2 nuclei. Percentages of host and HCMV genome reads are indicated. Insets show UCSC Genome Browser snapshots of native and crosslinked H3K4me3 DFF-ChIP Seq results for the host GAPDH gene, aligned with PRO-Seq nascent RNA reads in D-NT2 at 96 h pi.
(TIFF)

S11 Fig Viral IE2 and TBP are major components in the TBP-IE2-nucleosome complex at the MIEP. Nuclei of D-NT2 infected with HCMV for 96 h were subjected to DFF digestion and divided for TBP and IE2 ChIP Seq. The resulting DNA fragment reads were aligned to host (human hg38) and HCMV Towne (FJ616285.1) genomes (Exp 3, S2 Table). Top panels: UCSC Genome Browser views show TBP or viral IE2 occupancy across the MIEP region, spanning -800 bp upstream to +800 downstream of TSS for the MIEP. Bottom panels: FragMaps display DNA fragment distribution by length (left vertical axis) and relative abundance (right vertical axis), mapped to base coordinates in MIEP region (horizontal axis). All experimental procedures—including D-NT2 infected cell preparations, DFF-ChIP, library construction and Illumina sequencing—were performed in parallel. Green bracket highlights TBP-nucleosome fragments.
(TIFF)

S12 Fig The MIEP TBP-IE2-nucleosome complex contains higher levels of H3K4me3 and H3K27Ac, and H3.3 histones in D-NT2 compared to HFF. Nuclei of D-NT2 infected with HCMV for 96 h were subjected to DFF digestion and divided for H3K4me3 and H3K27Ac ChIP Seq. The resulting DNA fragment reads were aligned to host (human hg38) and HCMV Towne (FJ616285.1) genomes (Exp 3, S2 Table). Top panels: UCSC Genome Browser views show H3K4me3 or H3K27Ac occupancy across the MIEP (**A**) and RNA1.2 promoter (**B**) regions, spanning -800 bp upstream to +800 downstream of the TSSs. Bottom panels: FragMaps display DNA fragment distribution by length (left vertical axis) and relative abundance (right vertical axis), mapped to base coordinates in MIEP and RNA1.2 promoter regions (horizontal axis). All experimental procedures, including D-NT2 infected cell preparations, DFF-ChIP, library construction, and Illumina sequencing, were performed in parallel. Green bracket marks TBP-nucleosome fragments. (**C**) In a separate experiment, nuclei of D-NT2 and HFF infected in parallel with HCMV for 96 h were subjected to DFF digestion and divided for TBP, H3.3, and H3K4me3 ChIP Seq (Exp 5, S2 Table). UCSC Genome Browser views profile TBP, H3.3, and H3K4me3 occupancies across the MIEP region, spanning -800 bp to +800 relative to the MIEP TSS. All experimental procedures, including cell nuclei preparation, DFF-ChIP, library construction, and sequencing, were performed in parallel. PRO-Seq tracks show MIEP, EP1, EP2, and EP3 TSS positions (positions of 5’-ends of nascent RNAs) (Exp 5, S1 Table). (**D**) UCSC Genome Browser displays H3.3 occupancy across a 70-kbp HCMV genome region containing the RNA4.9 gene at 96 h after HFF vs D-NT2 infections, as described in panel C.
(TIFF)

S1 Table PRO-Seq datasets. (DOCX)

S2 Table DFF-ChIP seq datasets. (DOCX)

S3 Table RNA-seq datasets. (DOCX)

S4 Table Nucleic acid reagents. (DOCX)

## References

[1] Sinzger C, Digel M, Jahn G. Cytomegalovirus cell tropism. Curr Top Microbiol Immunol. 2008;325:63–83. doi: 10.1007/978-3-540-77349-8_4 18637500

[2] Cheeran MC-J, Lokensgard JR, Schleiss MR. Neuropathogenesis of congenital cytomegalovirus infection: disease mechanisms and prospects for intervention. Clin Microbiol Rev. 2009;22(1):99–126, Table of Contents. doi: 10.1128/CMR.00023-08 19136436 PMC2620634

[3] Towler JC, Ebrahimi B, Lane B, Davison AJ, Dargan DJ. Human cytomegalovirus transcriptome activity differs during replication in human fibroblast, epithelial and astrocyte cell lines. J Gen Virol. 2012;93(Pt 5):1046–58. doi: 10.1099/vir.0.038083-0 22258857 PMC3541802

[4] Schwartz M, Shnayder M, Nachshon A, Arazi T, Kitsberg Y, Levi Samia R, et al. Molecular characterization of human cytomegalovirus infection with single-cell transcriptomics. Nat Microbiol. 2023;8(3):455–68. doi: 10.1038/s41564-023-01325-x 36732471

[5] Cheng S, Caviness K, Buehler J, Smithey M, Nikolich-Žugich J, Goodrum F. Transcriptome-wide characterization of human cytomegalovirus in natural infection and experimental latency. Proc Natl Acad Sci U S A. 2017;114(49):E10586–95. doi: 10.1073/pnas.1710522114 29158406 PMC5724264

[6] Kwak H, Fuda NJ, Core LJ, Lis JT. Precise maps of RNA polymerase reveal how promoters direct initiation and pausing. Science. 2013;339(6122):950–3. doi: 10.1126/science.1229386 23430654 PMC3974810

[7] Herzog VA, Reichholf B, Neumann T, Rescheneder P, Bhat P, Burkard TR, et al. Thiol-linked alkylation of RNA to assess expression dynamics. Nat Methods. 2017;14(12):1198–204. doi: 10.1038/nmeth.4435 28945705 PMC5712218

[8] Erhard F, Baptista MAP, Krammer T, Hennig T, Lange M, Arampatzi P, et al. scSLAM-seq reveals core features of transcription dynamics in single cells. Nature. 2019;571(7765):419–23. doi: 10.1038/s41586-019-1369-y 31292545

[9] Parida M, Nilson KA, Li M, Ball CB, Fuchs HA, Lawson CK, et al. Nucleotide resolution comparison of transcription of human cytomegalovirus and host genomes reveals universal use of RNA polymerase II elongation control driven by dissimilar core promoter elements. mBio. 2019;10(1):e02047-18. doi: 10.1128/mBio.02047-18 30755505 PMC6372792

[10] Birkenheuer CH, Danko CG, Baines JD. Herpes simplex virus 1 dramatically alters loading and positioning of RNA polymerase II on host genes early in infection. J Virol. 2018;92(8):e02184-17. doi: 10.1128/JVI.02184-17 29437966 PMC5874419

[11] Dunn LEM, Lu F, Su C, Lieberman PM, Baines JD. Reactivation of epstein-barr virus from latency involves increased RNA polymerase activity at CTCF binding sites on the viral genome. J Virol. 2023;97(2):e0189422. doi: 10.1128/jvi.01894-22 36744959 PMC9972995

[12] Lodha M, Muchsin I, Jürges C, Juranic Lisnic V, L’Hernault A, Rutkowski AJ, et al. Decoding murine cytomegalovirus. PLoS Pathog. 2023;19(5):e1010992. doi: 10.1371/journal.ppat.1010992 37172056 PMC10208470

[13] Weiß E, Hennig T, Graßl P, Djakovic L, Whisnant AW, Jürges CS, et al. HSV-1 infection induces a downstream shift of promoter-proximal pausing for host genes. J Virol. 2023;97(5):e0038123. doi: 10.1128/jvi.00381-23 37093003 PMC10231138

[14] Li M, Ball CB, Collins G, Hu Q, Luse DS, Price DH, et al. Human cytomegalovirus IE2 drives transcription initiation from a select subset of late infection viral promoters by host RNA polymerase II. PLoS Pathog. 2020;16(4):e1008402. doi: 10.1371/journal.ppat.1008402 32251483 PMC7162547

[15] Li M, Hu Q, Collins G, Parida M, Ball CB, Price DH, et al. Cytomegalovirus late transcription factor target sequence diversity orchestrates viral early to late transcription. PLoS Pathog. 2021;17(8):e1009796. doi: 10.1371/journal.ppat.1009796 34339482 PMC8360532

[16] Ball CB, Li M, Parida M, Hu Q, Ince D, Collins GS, et al. Human cytomegalovirus IE2 both activates and represses initiation and modulates elongation in a context-dependent manner. mBio. 2022;13(3):e0033722. doi: 10.1128/mbio.00337-22 35579393 PMC9239164

[17] Stinski MF, Meier JL. Immediate-early viral gene regulation and function. In: Arvin A, Campadelli-Fiume G, Mocarski E, Moore PS, Roizman B, Whitley R, editors. Human Herpesviruses: Biology, Therapy, and Immunoprophylaxis. Cambridge. 2007. 21348071

[18] Gruffat H, Marchione R, Manet E. Herpesvirus late gene expression: a viral-specific pre-initiation complex is key. Front Microbiol. 2016;7:869. doi: 10.3389/fmicb.2016.00869 27375590 PMC4893493

[19] Spector BM, Parida M, Li M, Ball CB, Meier JL, Luse DS, et al. Differences in RNA polymerase II complexes and their interactions with surrounding chromatin on human and cytomegalovirus genomes. Nat Commun. 2022;13(1):2006. doi: 10.1038/s41467-022-29739-x 35422111 PMC9010409

[20] Ball CB, Parida M, Li M, Spector BM, Suarez GA, Meier JL, et al. Human cytomegalovirus infection elicits global changes in host transcription by RNA polymerases I, II, and III. Viruses. 2022;14(4):779. doi: 10.3390/v14040779 35458509 PMC9026722

[21] Spector BM, Santana JF, Pufall MA, Price DH. DFF-ChIP: a method to detect and quantify complex interactions between RNA polymerase II, transcription factors, and chromatin. Nucleic Acids Res. 2024;52(18):e88. doi: 10.1093/nar/gkae760 39248105 PMC11472042

[22] Luse DS, Parida M, Spector BM, Nilson KA, Price DH. A unified view of the sequence and functional organization of the human RNA polymerase II promoter. Nucleic Acids Res. 2020. 10.1093/nar/gkaa531 PMC7641323 32597978

[23] Gönczöl E, Andrews PW, Plotkin SA. Cytomegalovirus replicates in differentiated but not in undifferentiated human embryonal carcinoma cells. Science. 1984;224(4645):159–61. doi: 10.1126/science.6322309 6322309

[24] Meier JL. Reactivation of the human cytomegalovirus major immediate-early regulatory region and viral replication in embryonal NTera2 cells: role of trichostatin A, retinoic acid, and deletion of the 21-base-pair repeats and modulator. J Virol. 2001;75(4):1581–93. doi: 10.1128/JVI.75.4.1581-1593.2001 11160656 PMC114067

[25] Yuan J, Li M, Torres YR, Galle CS, Meier JL. Differentiation-coupled induction of human cytomegalovirus replication by union of the major enhancer retinoic acid, cyclic AMP, and NF-κB response elements. J Virol. 2015;89(24):12284–98. doi: 10.1128/JVI.00965-15 26423948 PMC4665231

[26] Chao SH, Price DH. Flavopiridol inactivates P-TEFb and blocks most RNA polymerase II transcription in vivo. J Biol Chem. 2001;276(34):31793–9. doi: 10.1074/jbc.M102306200 11431468

[27] Liu X, Yuan J, Wu AW, McGonagill PW, Galle CS, Meier JL. Phorbol ester-induced human cytomegalovirus major immediate-early (MIE) enhancer activation through PKC-delta, CREB, and NF-kappaB desilences MIE gene expression in quiescently infected human pluripotent NTera2 cells. J Virol. 2010;84(17):8495–508. doi: 10.1128/JVI.00416-10 20504934 PMC2919020

[28] Santana JF, Collins GS, Parida M, Luse DS, Price DH. Differential dependencies of human RNA polymerase II promoters on TBP, TAF1, TFIIB and XPB. Nucleic Acids Res. 2022;50(16):9127–48. doi: 10.1093/nar/gkac678 ; PMCID: PMC9458433. 35947745 PMC9458433

[29] Isomura H, Stinski MF, Kudoh A, Daikoku T, Shirata N, Tsurumi T. Two Sp1/Sp3 binding sites in the major immediate-early proximal enhancer of human cytomegalovirus have a significant role in viral replication. J Virol. 2005;79(15):9597–607. doi: 10.1128/JVI.79.15.9597-9607.2005 16014922 PMC1181558

[30] Stern-Ginossar N, Weisburd B, Michalski A, Le VTK, Hein MY, Huang S-X, et al. Decoding human cytomegalovirus. Science. 2012;338(6110):1088–93. doi: 10.1126/science.1227919 23180859 PMC3817102

[31] Albright ER, Kalejta RF. Canonical and variant forms of histone h3 are deposited onto the human cytomegalovirus genome during lytic and latent infections. J Virol. 2016;90(22):10309–20. doi: 10.1128/JVI.01220-16 27605676 PMC5105665

[32] Meier JL, Stinski MF. Regulation of human cytomegalovirus immediate-early gene expression. Intervirology. 1996;39(5–6):331–42. doi: 10.1159/000150504 9130043

[33] Meier JL, Stinski MF. Major immediate-early enhancer and its gene products. In: Reddehase MJ, editor. Cytomegaloviruses: Molecular Biology and Immunology. Norfolk, UK.: Caister Academic Press; 2006. p. 151–66.

[34] Forte E, Li M, Ayaloglu Butun F, Hu Q, Borst EM, Schipma MJ, et al. Critical role for the human cytomegalovirus major immediate early proteins in recruitment of RNA polymerase II and H3K27Ac to an enhancer-like element in oriLyt. Microbiol Spectr. 2023;11(1):e0314422. doi: 10.1128/spectrum.03144-22 36645269 PMC9927211

[35] Forte E, Ayaloglu Butun F, Marinaccio C, Schipma MJ, Piunti A, Schroeder MW, et al. Epigenetic reprogramming of host and viral genes by Human Cytomegalovirus infection in Kasumi-3 myeloid progenitor cells at early times post-infection. J Virol. 2021;95(11):e00183-21. doi: 10.1128/JVI.00183-21 33731453 PMC10021080

[36] Reeves M, Murphy J, Greaves R, Fairley J, Brehm A, Sinclair J. Autorepression of the human cytomegalovirus major immediate-early promoter/enhancer at late times of infection is mediated by the recruitment of chromatin remodeling enzymes by IE86. J Virol. 2006;80(20):9998–10009. doi: 10.1128/JVI.01297-06 17005678 PMC1617317

[37] Matthews SM, Groves IJ, O’Connor CM. Chromatin control of human cytomegalovirus infection. mBio. 2023;14(4):e0032623. doi: 10.1128/mbio.00326-23 37439556 PMC10470543

[38] Collins-McMillen D, Kamil J, Moorman N, Goodrum F. Control of immediate early gene expression for human cytomegalovirus reactivation. Front Cell Infect Microbiol. 2020;10:476. doi: 10.3389/fcimb.2020.00476 33072616 PMC7533536

[39] Nitzsche A, Paulus C, Nevels M. Temporal dynamics of cytomegalovirus chromatin assembly in productively infected human cells. J Virol. 2008;82(22):11167–80. doi: 10.1128/JVI.01218-08 18786996 PMC2573275

[40] Nitzsche A, Steinhäusser C, Mücke K, Paulus C, Nevels M. Histone H3 lysine 4 methylation marks postreplicative human cytomegalovirus chromatin. J Virol. 2012;86(18):9817–27. doi: 10.1128/JVI.00581-12 22761369 PMC3446588

[41] Manska S, Rossetto CC. Characteristics of immediate-Early 2 (IE2) and UL84 proteins in UL84-independent strains of human cytomegalovirus (HCMV). Microbiol Spectr. 2021;9(2):e0053921. doi: 10.1128/Spectrum.00539-21 34550009 PMC8557881

[42] Yuan J, Liu X, Wu AW, McGonagill PW, Keller MJ, Galle CS, et al. Breaking human cytomegalovirus major immediate-early gene silence by vasoactive intestinal peptide stimulation of the protein kinase A-CREB-TORC2 signaling cascade in human pluripotent embryonal NTera2 cells. J Virol. 2009;83(13):6391–403. doi: 10.1128/JVI.00061-09 19369332 PMC2698552

[43] Meier JL, Stinski MF. Effect of a modulator deletion on transcription of the human cytomegalovirus major immediate-early genes in infected undifferentiated and differentiated cells. J Virol. 1997;71(2):1246–55. doi: 10.1128/JVI.71.2.1246-1255.1997 8995648 PMC191179

[44] Marchini A, Liu H, Zhu H. Human cytomegalovirus with IE-2 (UL122) deleted fails to express early lytic genes. J Virol. 2001;75(4):1870–8. doi: 10.1128/JVI.75.4.1870-1878.2001 11160686 PMC114097

[45] Warming S, Costantino N, Court DL, Jenkins NA, Copeland NG. Simple and highly efficient BAC recombineering using galK selection. Nucleic Acids Res. 2005;33(4):e36. doi: 10.1093/nar/gni035 15731329 PMC549575

[46] Nilson KA, Lawson CK, Mullen NJ, Ball CB, Spector BM, Meier JL, et al. Oxidative stress rapidly stabilizes promoter-proximal paused Pol II across the human genome. Nucleic Acids Res. 2017;45(19):11088–105. doi: 10.1093/nar/gkx724 28977633 PMC5737879

[47] Langmead B, Trapnell C, Pop M, Salzberg SL. Ultrafast and memory-efficient alignment of short DNA sequences to the human genome. Genome Biol. 2009;10(3):R25. doi: 10.1186/gb-2009-10-3-r25 19261174 PMC2690996

[48] Quinlan AR, Hall IM. BEDTools: a flexible suite of utilities for comparing genomic features. Bioinformatics. 2010;26(6):841–2. doi: 10.1093/bioinformatics/btq033 20110278 PMC2832824

[49] Kent WJ, Sugnet CW, Furey TS, Roskin KM, Pringle TH, Zahler AM, et al. The human genome browser at UCSC. Genome Res. 2002;12(6):996–1006. doi: 10.1101/gr.229102 12045153 PMC186604

[50] Ball CB, Nilson KA, Price DH. Use of the nuclear walk-on methodology to determine sites of RNA polymerase II initiation and pausing and quantify nascent RNAs in cells. Methods. 2019;159–160:165–76. doi: 10.1016/j.ymeth.2019.02.003 30743000 PMC6589122

[51] Tarazona S, Furió-Tarí P, Turrà D, Pietro AD, Nueda MJ, Ferrer A, et al. Data quality aware analysis of differential expression in RNA-seq with NOISeq R/Bioc package. Nucleic Acids Res. 2015;43(21):e140. doi: 10.1093/nar/gkv711 26184878 PMC4666377

