# Supplementary figures and images for: Cell type differences in human cytomegalovirus transcription and epigenetic regulation with insights into major immediate-early enhancer-promoter control
Source: PLoS Pathog. 2025 Aug 4;21(8):e1013374. doi: 10.1371/journal.ppat.1013374 (PMC12333995; doi:10.1371/journal.ppat.1013374)

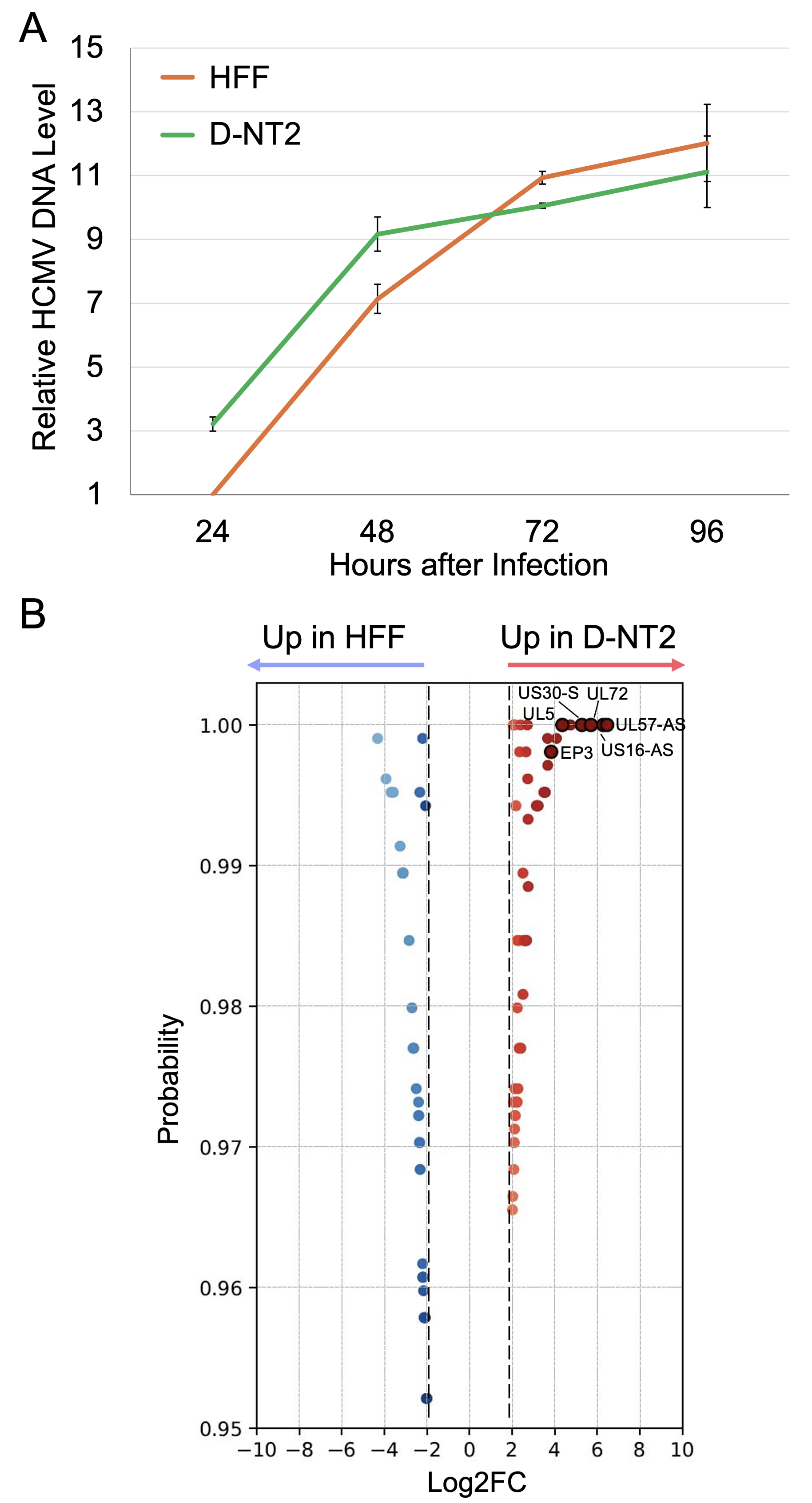

Supplement: S1 Fig — (A) D-NT2 (MOI 6.0, determined in HFF) and HFF (MOI 2.0) were infected in parallel with HCMV. Nuclei from duplicate infections were prepared at 24, 48, 72, and 96 h pi, using the method applied in PRO-Seq. Mean and range of results of HCMV DNA levels, measured by qPCR and normalized to human GAPDH DNA levels, are shown relative the HCMV DNA level in HFF nuclei at 24 h pi. (B) HFF and D-NT2 were infected in separate experiments for 96 h, with Flavo added during the final hour to assess viral TSS usage. PRO-Seq was performed for nascent RNA quantification and sequence analysis, and viral TSS strength was determined for each active viral promoter. PRO-Seq datasets for HFF (GSE139114) and D-NT2 (Exp 2, S1 Table) were normalized for total viral reads, excluding reads from the RNA4.9 region, to allow comparison of transcription across the HCMV genome in HFF vs D-NT2. Differential analysis identified 97 viral promoters with ≥4-fold differences in TSS strength and their estimated probability of differential expression ≥0.995 (1 minus the p-value ≤0.005), represented as blue or red dots. Darker shades indicate overlapping data points. Six viral TSSs (UL5, UL72, EP3, UL57-AS, US16-AS, and US30-S promoters) that are ≥ 15-fold more active in D-NT2 than in HFF are marked with large dark red dots and selected for further analyses. Differences in experimental conditions and depth of sequencing between the two groups resulted in fewer evaluable viral TSSs with normalized TSS strength >200 reads (see Methods). Consequently, fewer viral promoters met the ≥ 4-fold difference threshold compared to the analysis in Fig 1D. Nonetheless, 89% of the viral promoters identified here were concordant with those shown in Fig 1D (TIFF) [file ppat.1013374.s001.tiff]

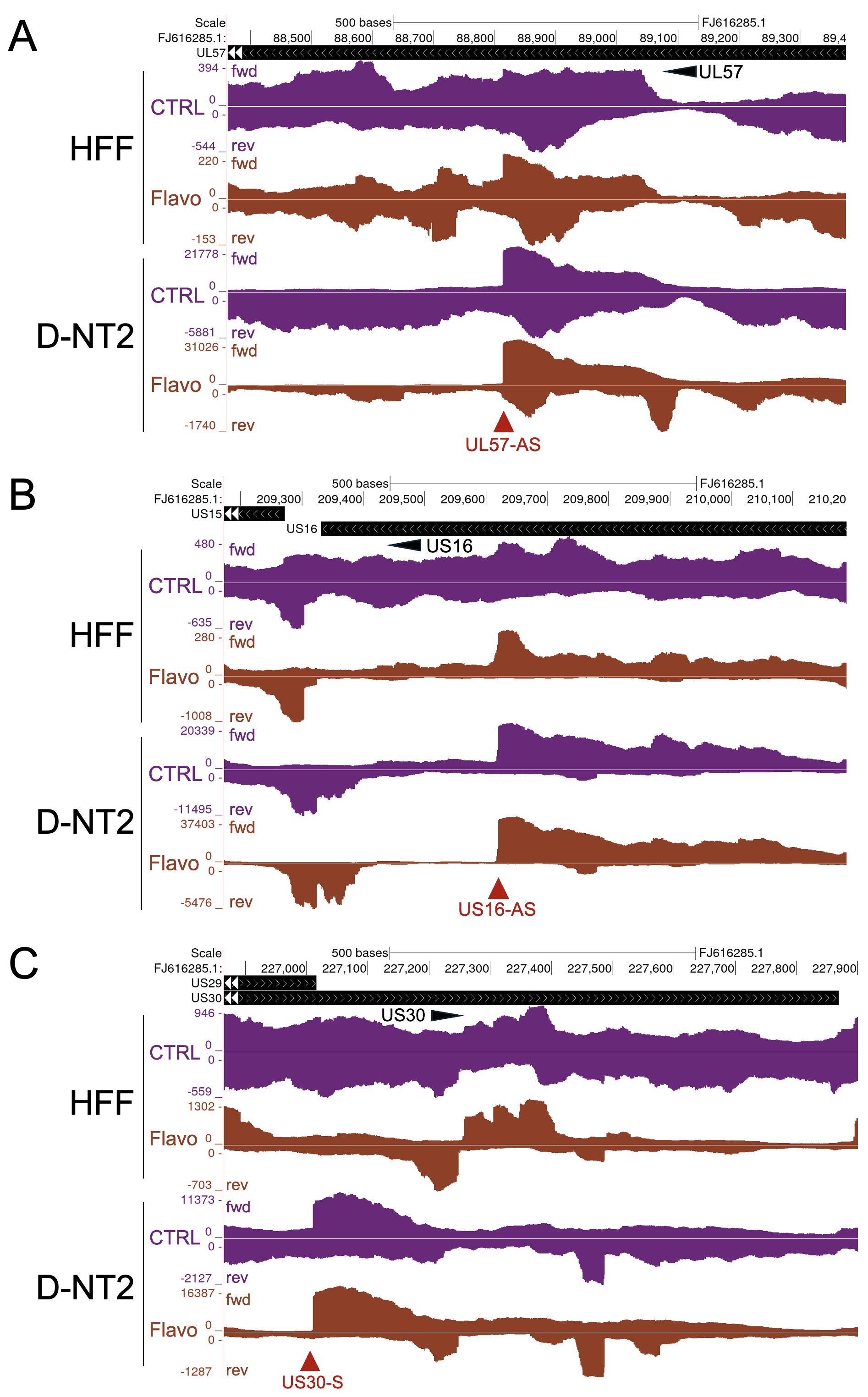

Supplement: S2 Fig — PRO-Seq was carried out at 96 h pi of HFF (GSE139114) and D-NT2 (Exp 2, S1 Table) with CTRL or Flavo, as detailed in Fig 2 legend. Auto-scaled UCSC genome Browser views of spike-in normalized nascent RNA reads aligned to the annotated HCMV Towne genome (FJ616285.1) show HCMV long promoters for UL57-antisense (UL57-AS) (A), US16-AS (B), and US30-sense nascent RNA arising internal to US30 ORF (US30-S) (C). (TIFF) [file ppat.1013374.s002.tiff]

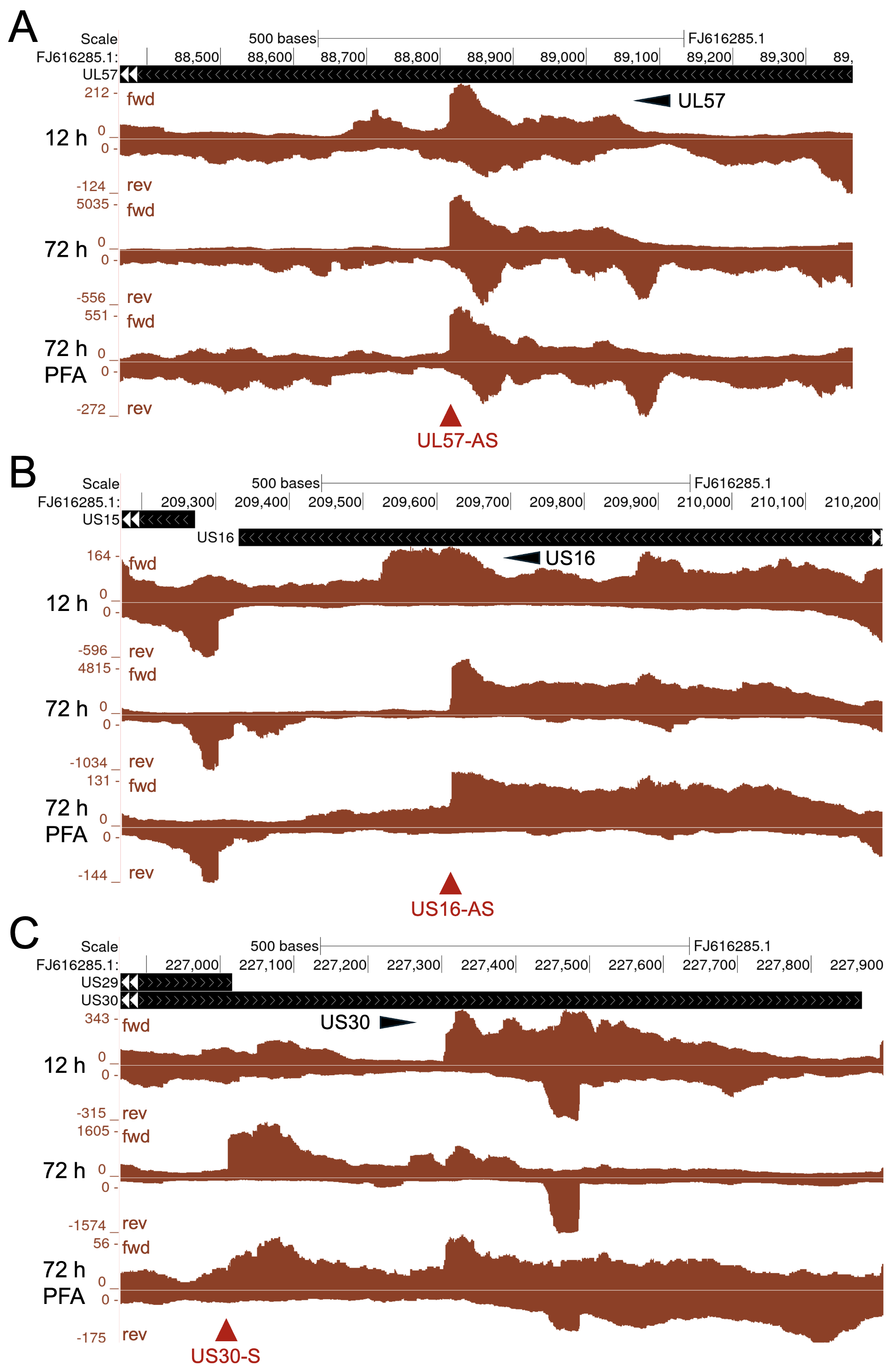

Supplement: S3 Fig — D-NT2 were infected for 12 h, 72 h, and 72 h in the presence of PFA inhibitor of HCMV DNA replication, as detailed in the Fig 3 legend (Exp 1, S1 Table). Auto-scaled spike-in normalized UCSC genome Browser views of nascent RNA reads aligned to the annotated HCMV Towne genome (FJ616285.1) at viral long promoters for UL57-AS (A), US16-AS (B), and US30-S (C) nascent RNAs. Dark red arrowheads point to TSSs of viral long promoters. Abbreviation: phosphonoformic acid, PFA. (TIFF) [file ppat.1013374.s003.tiff]

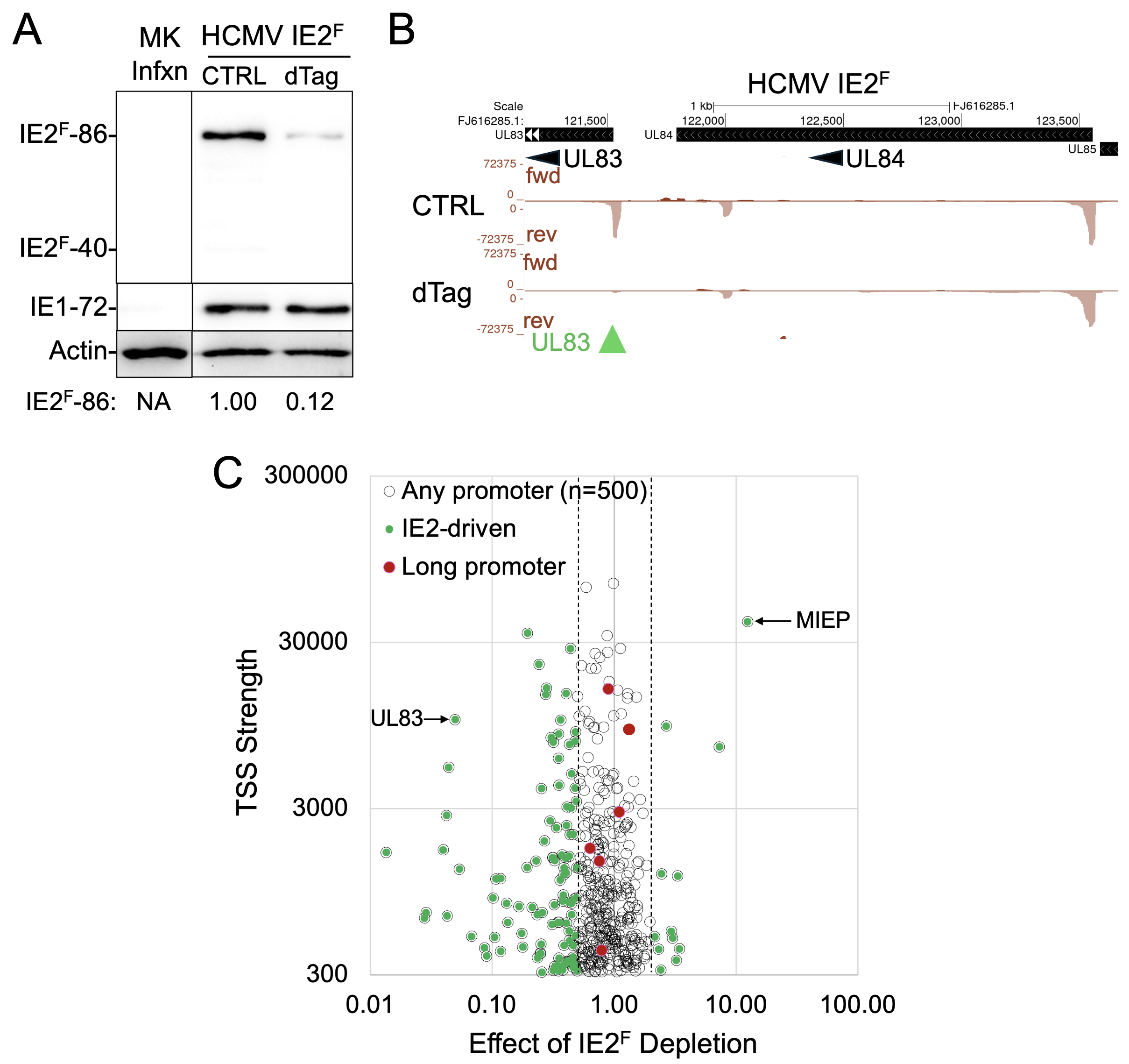

Supplement: S4 Fig — D-NT2 were infected for 96 h with HCMV IE2F and treated with dTag degrader vs CTRL for the last 6 h of infection, as detailed in Fig 5 legend. (A) Western blot analysis shows that the dTag degrader decreased IE2F-86 amount by 88%, compared to CTRL and normalized to the host actin control. In contrast, levels of viral IE1 had not changed. (B, C) Spike-in normalized nascent RNAs generated by PRO-Seq coupled with Flavo were aligned to the annotated HCMV Towne genome (FJ616285.1) (Exp 4, S1 Table). (B) UCSC genome Browser views of the effects of dTag vs CTRL on viral UL83 and UL84 promoters. Vertical scales were set to view dTag treatment effects on amount of nascent RNA reads produced by UL83 and UL84 promoters. Green arrowhead points to position of IE2-driven UL83 promoter. (C) Effect of IE2F depletion on strength of each of the top 500 viral TSSs minus the RNA4.9 promoter region (any promoter) was plotted against the strength of each viral TSS in absence of dTag degrader. Viral TSSs with strengths that increase ≥2-fold or decrease by ≥50% by IE2 depletion (demarcated by hatched lines) are deemed as IE2-responsive and marked by green dots, whereas viral long promoters are marked by red dots. (TIFF) [file ppat.1013374.s004.tiff]

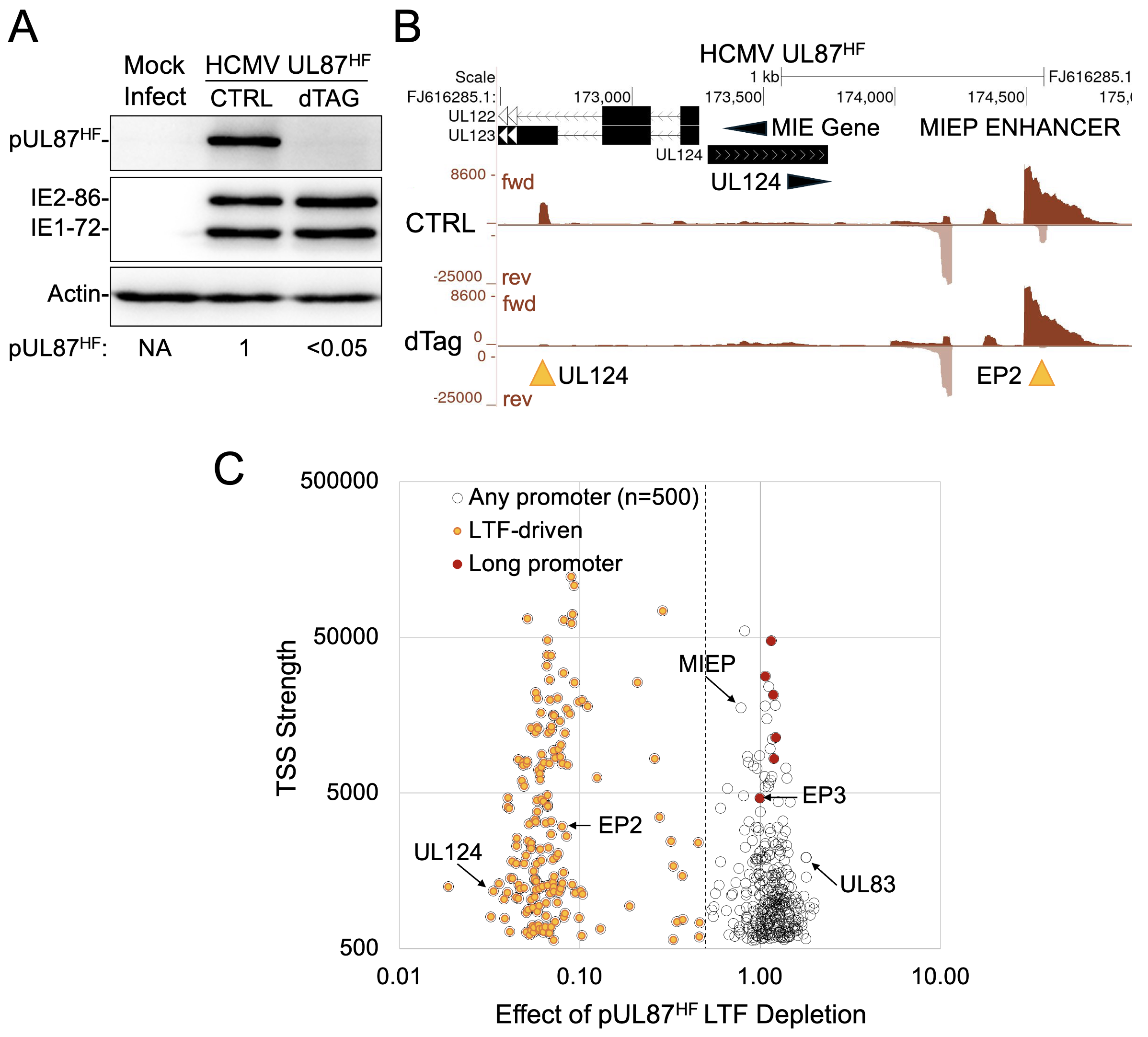

Supplement: S5 Fig — (A) Western blot assessment of change in FKBP12F36V-tagged UL87 LTF (pUL87HF) amount in HCMV-infected D-NT2 after exposed to the dTag degrader vs vehicle control (CTRL) from 90-96 h pi, compared to levels of viral IE1 and IE2, and host actin. (B, C) In parallel studies, spike-in controlled PRO-Seq was applied to quantify change in viral promoter strength 1 h after Flavo was added to the infected cells (Exp 3, S1 Table). (B) UCSC genome Browser view of results for the MIE gene and promoter/enhancer regions. Gold arrowheads point to positions of UL87 LTF-driven UL124 and EP2 promoters. (C) Effect of pUL87HF LTF depletion on viral TSS strength plotted vs viral TSS strength in absence of dTag degrader for the top 500 viral TSSs (any promoter). Gold and dark red dots mark LTF-driven promoters and long promoters, respectively. Arrowheads point to MIEP, EP2, and EP3, as well as UL83 and UL124 promoters. (TIFF) [file ppat.1013374.s005.tiff]

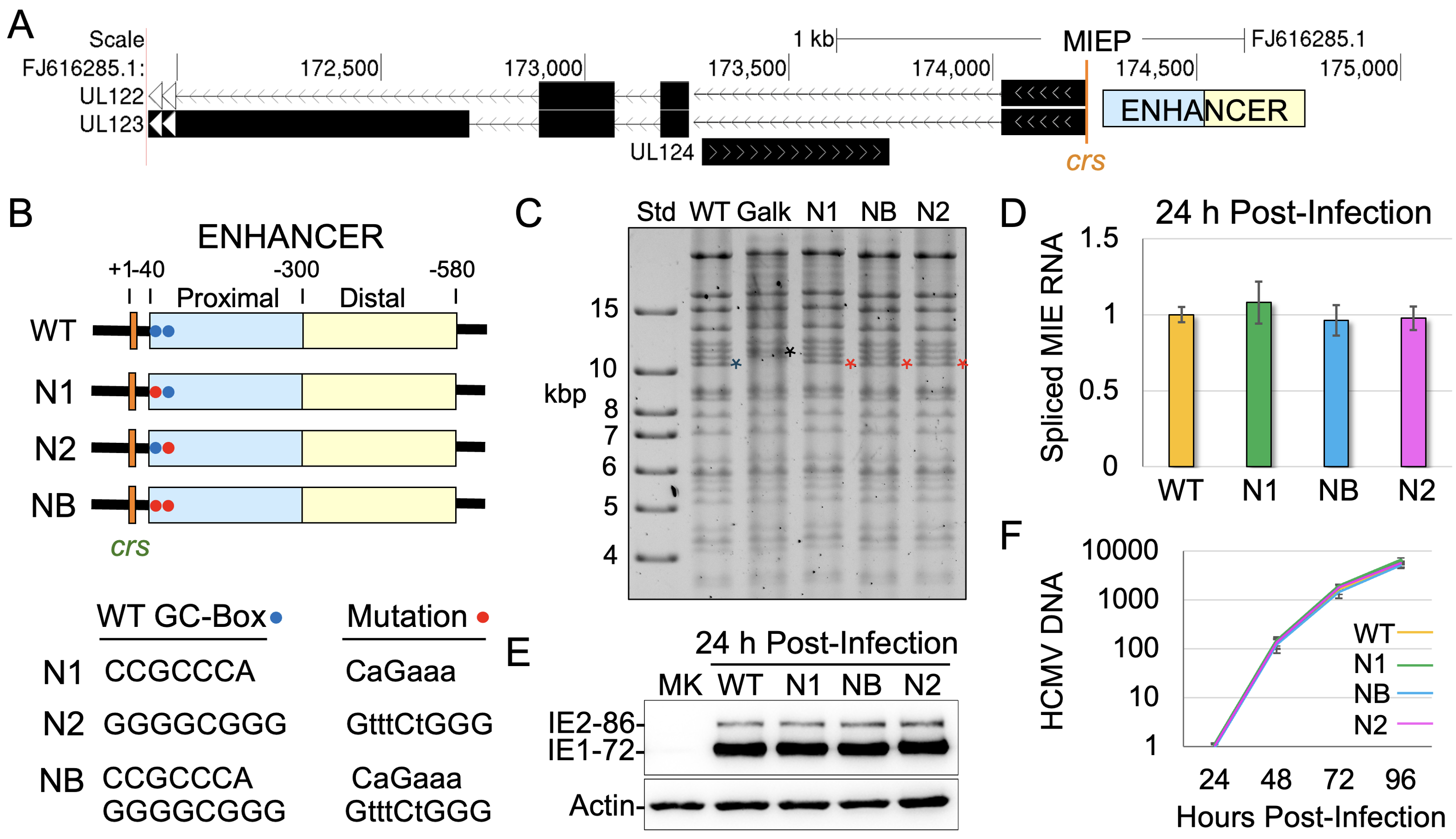

Supplement: S6 Fig — (A) Annotated section of HCMV Towne genome (FJ616285.1) showing the MIEP, enhancer, cis-repression sequence (crs), and the UL122 and UL123 open reading frames encoding IE2–86 and IE1–72, respectively. (B, C) Schematic representation of site-directed mutations (SDMs) introduced into the N1, N2, and NB GC-boxes in the proximal MIE enhancer (B) and the electrophoretic pattern of EcoRI fragments (C) from HCMV genomes carrying N1, N2, and NB SDMs, compared to the WT HCMV genome. Blue and red asterisks indicate WT and mutated fragments, respectively. (D-F) Comparison of viruses at MOI 1.0 in HFF at 24 h pi includes levels of spliced MIE RNA (D) and levels of IE1–72 and IE2–86 proteins relative to host actin (E). HCMV DNA levels at MOI 0.05 were assessed at 24, 48, 72, and 96 h pi, expressed relative to WT DNA at 24 h pi (F). Viral RNA and DNA from triplicate infections were quantified by PCR and normalized to host GAPDH RNA and DNA, respectively (D, F). WT, N1, N2, and NB DNA levels at 24 h pi were 1.00, 1.07, 1.05, and 0.96, respectively. (TIFF) [file ppat.1013374.s006.tiff]

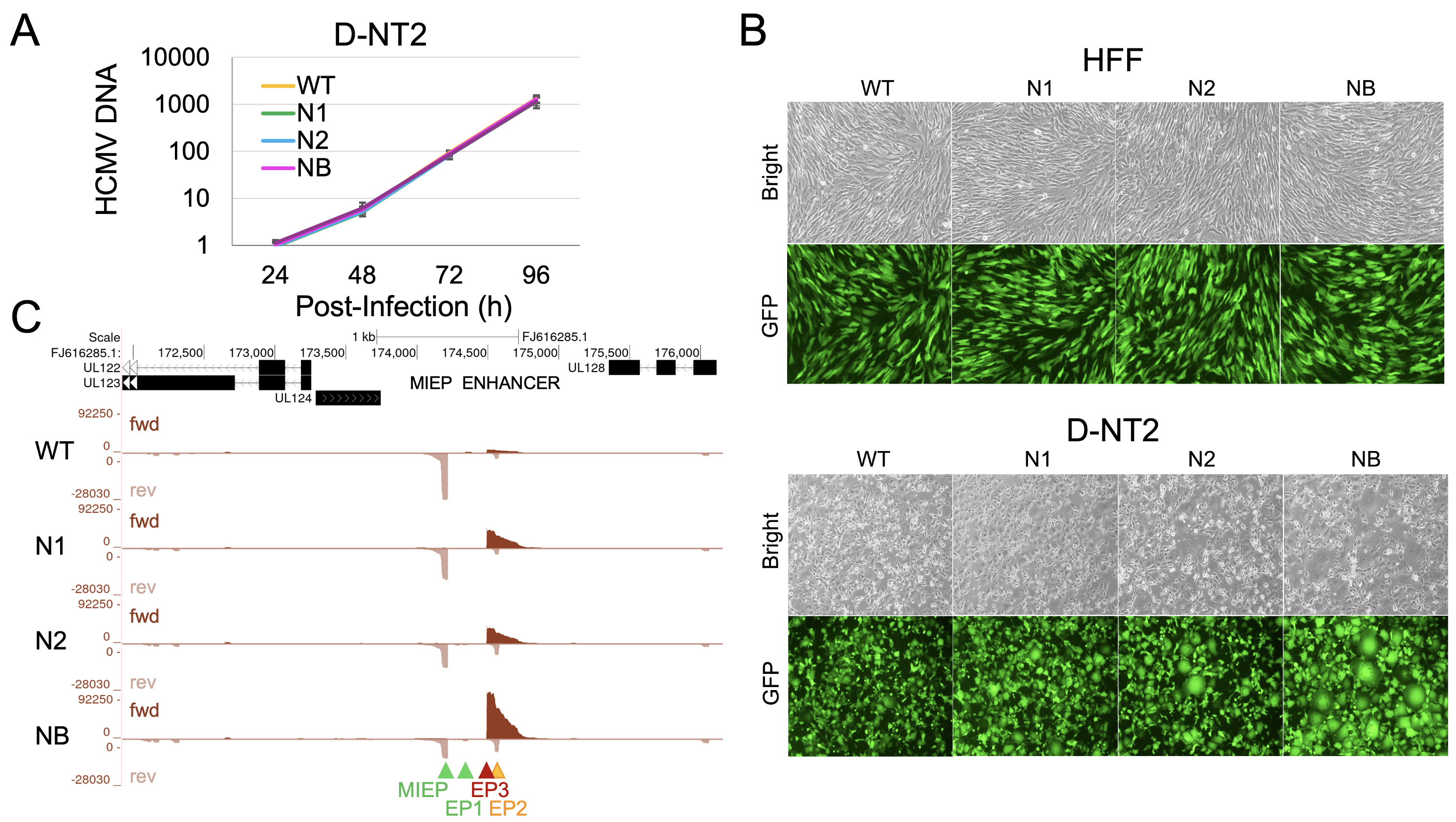

Supplement: S7 Fig — (A) D-NT2 were inoculated with equal amounts (MOI 3) of WT, N1, N2, and NB viruses, and viral DNA levels were measured by qPCR at 24, 48, 72, and 96 h pi. Relative DNA levels at 24 h pi was 1.00, 0.94, 0.96, and 1.02 for WT, N1, N2, and NB, respectively. (B) HFF (MOI 3) and D-NT2 (MOI 5) were infected in parallel with WT, N1, N2, and NB viruses for 96 h pi. Cell morphology and viral GFP fluorescence were visualized using bright-field and fluorescence microscopy (original magnification, 10x). (C) D-NT2 infected with WT, N1, N2, and NB viruses (MOI 5) in panel B were also subjected to PRO-Seq coupled with Flavo (Exp 2, S1 Table). Spike-in normalized reads from N1, N2, and NB infections were aligned to HCMV Towne genomes (FJ616285.1) incorporating N1, N2, and NB mutations, respectively. UCSC Genome Browser views depict the HCMV region containing MIEP and the enhancer, with scales set to facilitate comparison of MIEP and EP3 nascent RNA levels. (TIFF) [file ppat.1013374.s007.tiff]

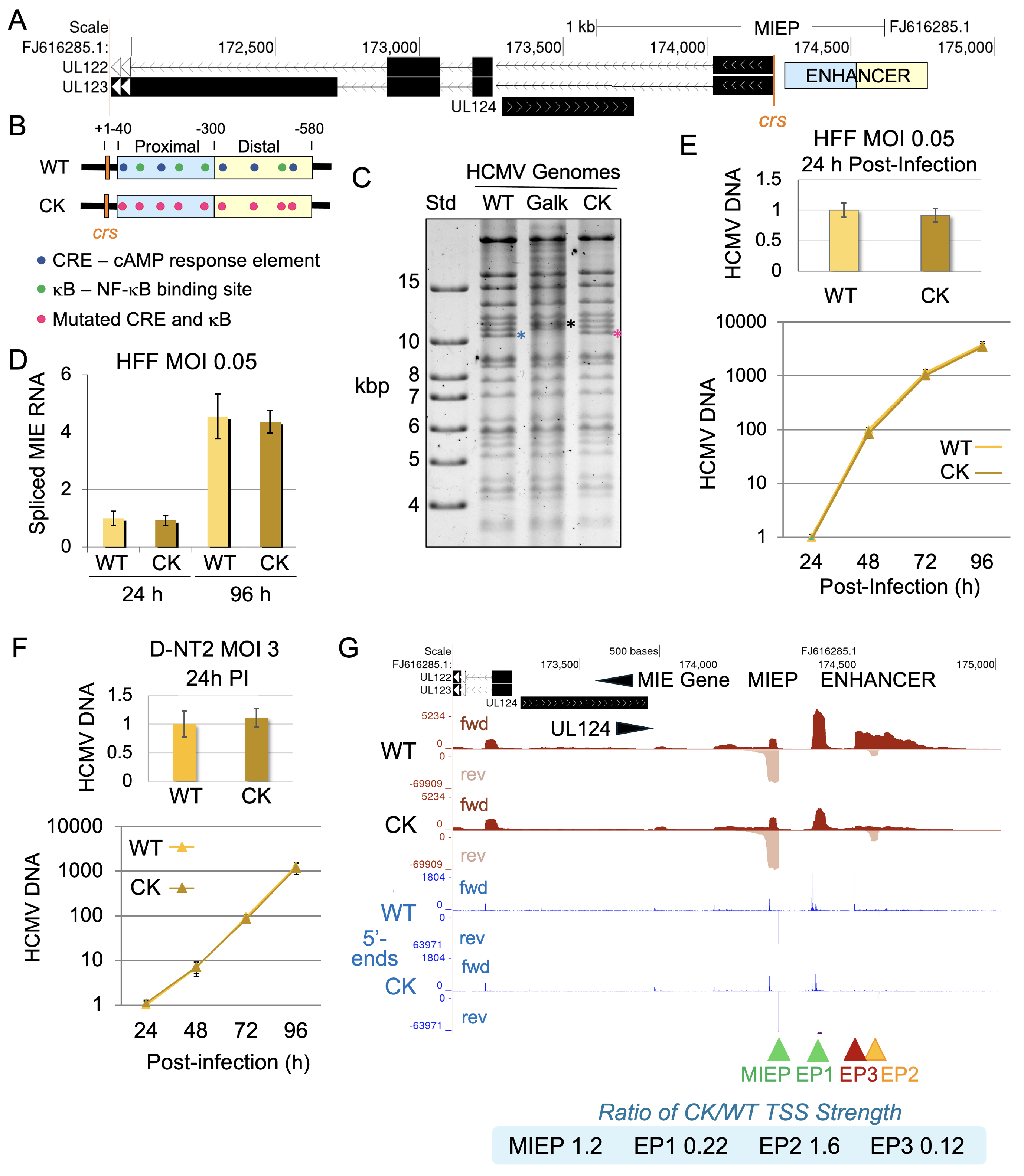

Supplement: S8 Fig — (A) Annotated HCMV genome region showing the MIEP, enhancer, cis-repression sequence (crs) and UL122 and UL123 open reading frames for IE2–86 and IE1–72, respectively. (B, C) Schematic of mutations in CRE and kB mutations (CK virus) (B), and of EcoRI fragment electrophoretic pattern (C) comparing mutated CK genomes with WT and GalK intermediate genomes. Blue and red asterisks denote WT and mutated fragments, respectively. (D-E) Comparative analysis of WT and CK viruses at MOI 0.05, assessing spliced IE1 and IE2 RNA expression at 24 and 96 h pi (D) and viral DNA produced at 24, 48, 72, and 96 h pi, relative to WT DNA at 24 h (E). (F) HCMV DNA levels in D-NT2 infected at MOI 3 were assessed at 24, 48, 72, and 96 h pi. Viral RNA and DNA from triplicate infections were quantified by PCR and normalized to host GAPDH RNA and DNA, respectively. (G) UCSC Genome Browser view of spike-in normalized nascent RNA reads aligned to the HCMV MIE gene regulatory region (FJ616285.1). PRO-Seq coupled to Flavo was used to measure MIEP and EP3 nascent RNA levels, with scales adjusted to enable direct comparisons (Exp 4, S1 Table). Corresponding tracks show the base positions of 5’-ends of nascent RNAs at viral TSSs. Inset (G) displays the ratio of CK/WT TSS strength for MIEP, EP1, EP2, and EP3 promoters. (TIFF) [file ppat.1013374.s008.tiff]

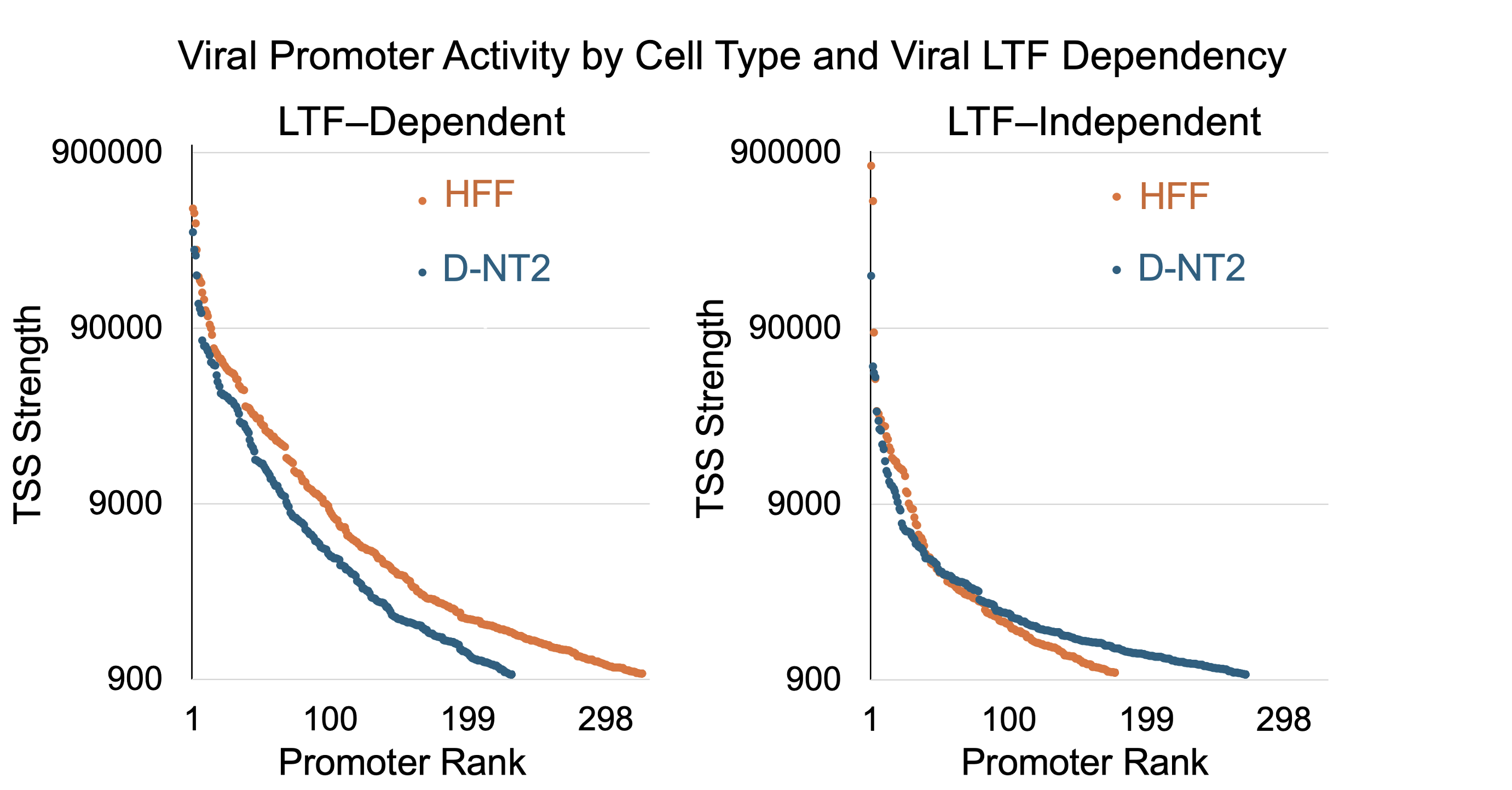

Supplement: S9 Fig — HFF and D-NT2 were infected for 96 h in parallel with HCMV, and PRO-Seq coupled with Flavo was used to quantify 5′-ends of viral nascent RNA reads for the top 500 most active viral TSSs (Exp 5, S1 Table). Total viral reads were normalized between HFF and D-NT2 infections. Graphs display viral TSS strengths (number of reads) for LTF-dependent and LTF-independent viral promoters, ranked by promoter strength in HFF (orange red) and D-NT2 (blue) infections. (TIFF) [file ppat.1013374.s009.tiff]

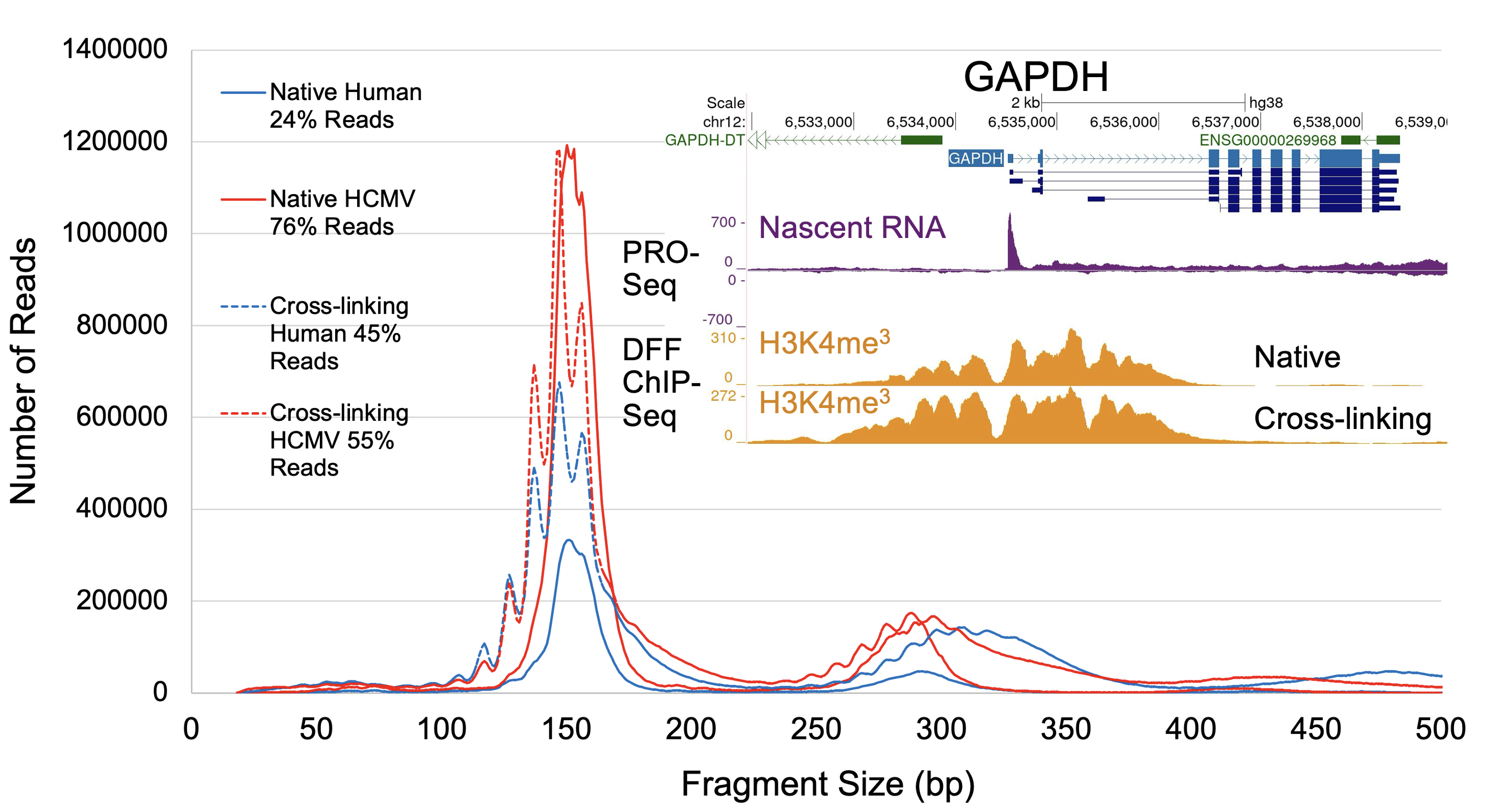

Supplement: S10 Fig — D-NT2 were infected with HCMV for 96 h and treated with or without paraformaldehyde crosslinking before DFF-ChIP for H3K4me3 (Exp 4, S2 Table). DNA fragment reads were aligned to the host (human hg38) and HCMV Towne (FJ616285.1) genomes. The graph compares the number of reads and fragment sizes (18–500 bp) for native (solid lines) and crosslinked (hatched lines) DNA fragments from the host genome (blue) and HCMV genome (red). Total viral reads were normalized to account for a 27% difference in GAPDH-normalized viral DNA quantities between native and crosslinked D-NT2 nuclei. Percentages of host and HCMV genome reads are indicated. Insets show UCSC Genome Browser snapshots of native and crosslinked H3K4me3 DFF-ChIP Seq results for the host GAPDH gene, aligned with PRO-Seq nascent RNA reads in D-NT2 at 96 h pi. (TIFF) [file ppat.1013374.s010.tiff]

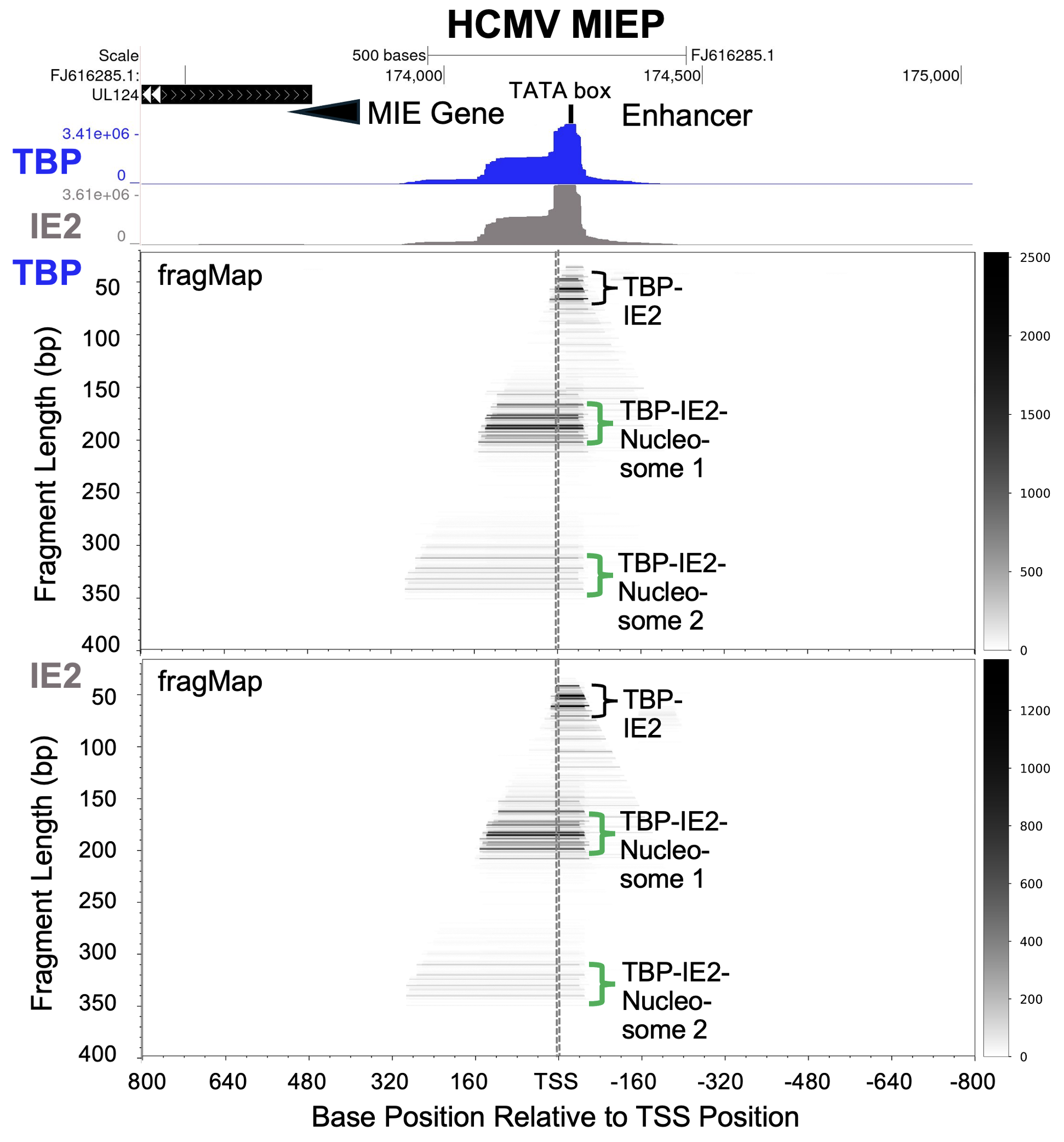

Supplement: S11 Fig — Nuclei of D-NT2 infected with HCMV for 96 h were subjected to DFF digestion and divided for TBP and IE2 ChIP Seq. The resulting DNA fragment reads were aligned to host (human hg38) and HCMV Towne (FJ616285.1) genomes (Exp 3, S2 Table). Top panels: UCSC Genome Browser views show TBP or viral IE2 occupancy across the MIEP region, spanning -800 bp upstream to +800 downstream of TSS for the MIEP. Bottom panels: FragMaps display DNA fragment distribution by length (left vertical axis) and relative abundance (right vertical axis), mapped to base coordinates in MIEP region (horizontal axis). All experimental procedures—including D-NT2 infected cell preparations, DFF-ChIP, library construction and Illumina sequencing—were performed in parallel. Green bracket highlights TBP-nucleosome fragments. (TIFF) [file ppat.1013374.s011.tiff]

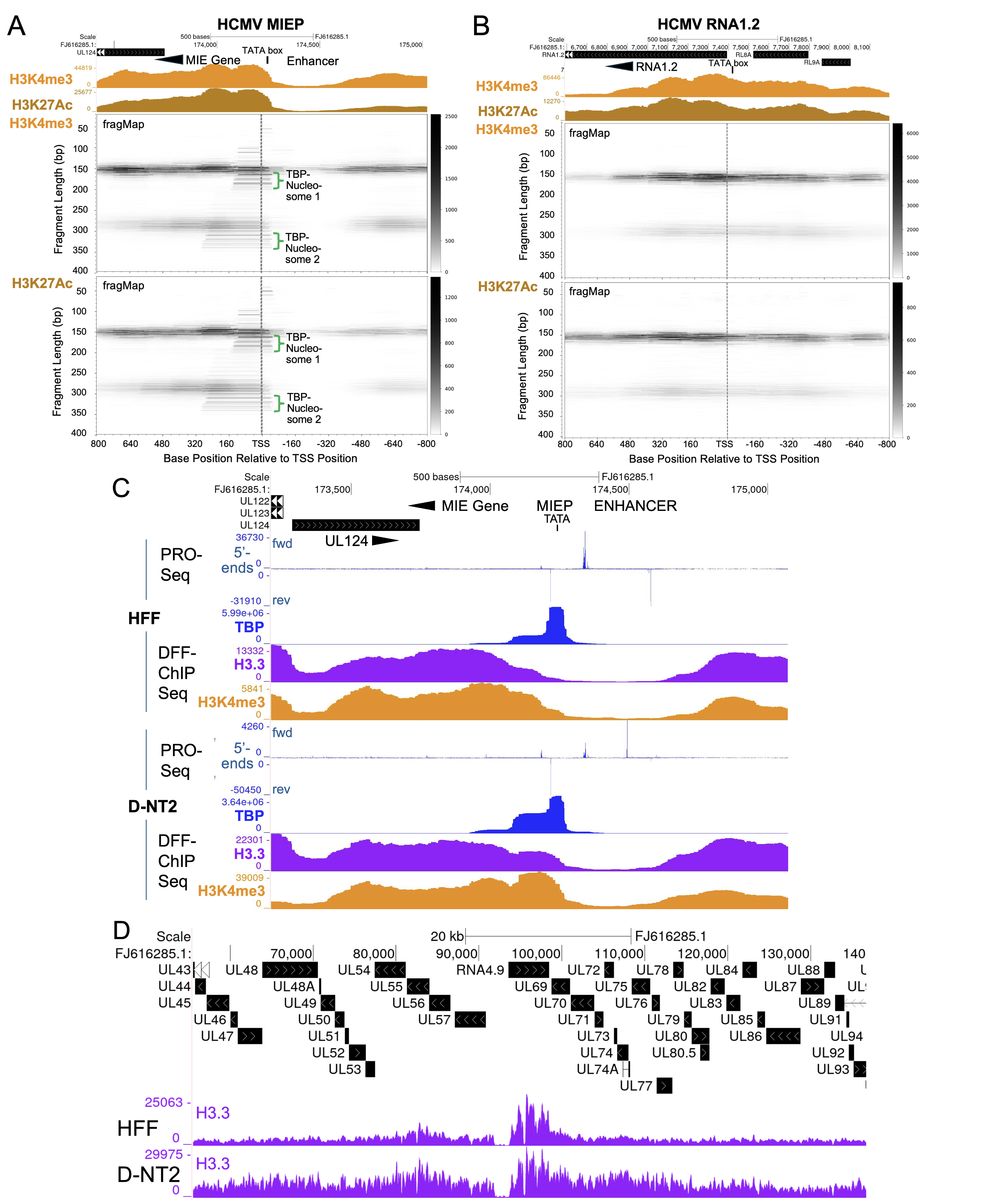

Supplement: S12 Fig — Nuclei of D-NT2 infected with HCMV for 96 h were subjected to DFF digestion and divided for H3K4me3 and H3K27Ac ChIP Seq. The resulting DNA fragment reads were aligned to host (human hg38) and HCMV Towne (FJ616285.1) genomes (Exp 3, S2 Table). Top panels: UCSC Genome Browser views show H3K4me3 or H3K27Ac occupancy across the MIEP (A) and RNA1.2 promoter (B) regions, spanning -800 bp upstream to +800 downstream of the TSSs. Bottom panels: FragMaps display DNA fragment distribution by length (left vertical axis) and relative abundance (right vertical axis), mapped to base coordinates in MIEP and RNA1.2 promoter regions (horizontal axis). All experimental procedures, including D-NT2 infected cell preparations, DFF-ChIP, library construction, and Illumina sequencing, were performed in parallel. Green bracket marks TBP-nucleosome fragments. (C) In a separate experiment, nuclei of D-NT2 and HFF infected in parallel with HCMV for 96 h were subjected to DFF digestion and divided for TBP, H3.3, and H3K4me3 ChIP Seq (Exp 5, S2 Table). UCSC Genome Browser views profile TBP, H3.3, and H3K4me3 occupancies across the MIEP region, spanning -800 bp to +800 relative to the MIEP TSS. All experimental procedures, including cell nuclei preparation, DFF-ChIP, library construction, and sequencing, were performed in parallel. PRO-Seq tracks show MIEP, EP1, EP2, and EP3 TSS positions (positions of 5’-ends of nascent RNAs) (Exp 5, S1 Table). (D) UCSC Genome Browser displays H3.3 occupancy across a 70-kbp HCMV genome region containing the RNA4.9 gene at 96 h after HFF vs D-NT2 infections, as described in panel C. (TIFF) [file ppat.1013374.s012.tiff]
