# Supplementary material for: Cell type differences in human cytomegalovirus transcription and epigenetic regulation with insights into major immediate-early enhancer-promoter control
Source: PLoS Pathog. 2025 Aug 4;21(8):e1013374. doi: 10.1371/journal.ppat.1013374 (PMC12333995; doi:10.1371/journal.ppat.1013374)
Supplement: S1 Table — (DOCX) [file ppat.1013374.s013.docx]

| **S1 TABLE.** **PRO-Seq Datasets*** | | | | | |
| --- | --- | --- | --- | --- | --- |
| **Exp1 D-NT2 WT datasets** | **Total deduplicated reads** | **Total read correction factor** | **Human mapped reads** | **HCMV mapped reads** | **Spike-in mapped reads** |
| Exp1 12 hpi No Flavo | 85680869 | 0.921527426 | 81833412 | 3453393 | 394064 |
| Exp1 12 hpi Flavo | 90119373 | 0.655125738 | 88537728 | 955718 | 625927 |
| Exp1 Mock Flavo | 64883640 | 0.909929233 | 64462772 | 21601 | 399267 |
| Exp1 72 hpi No Flavo | 67262287 | 1.173871335 | 50095150 | 16863917 | 303220 |
| Exp1 72 hpi No Flavo 72 h PFA | 83928656 | 0.940766532 | 73754563 | 9769815 | 404278 |
| Exp1 72 hpi Flavo | 43812588 | 1.347546982 | 37275736 | 6240396 | 296456 |
| Exp1 72 hpi Flavo  72 h PFA | 37342482 | 1.581028298 | 36163440 | 853069 | 325973 |
| **Exp1 D-NT2 WT datasets** | **Corrected human reads** | **Corrected HCMV reads** | **Corrected Spike-in reads** | **Spike-in correction factor** | **Final correction factor** |
| Exp1 12 hpi No Flavo | 75411734 | 3182396 | 363141 | 1.01 | 0.93 |
| Exp1 12 hpi Flavo | 58003344 | 626115 | 410061 | 1.03 | 0.67 |
| Exp1 Mock Flavo | 58656561 | 19655 | 363305 | 1.16 | 1.06 |
| Exp1 72 hpi No Flavo | 58805261 | 19796069 | 355941 | 1.03 | 1.21 |
| Exp1 72 hpi No Flavo 72 h PFA | 69385824 | 9191115 | 380331 | 0.96 | 0.91 |
| Exp1 72 hpi Flavo | 50230806 | 8409227 | 399488 | 1.06 | 1.42 |
| Exp1 72 hpi Flavo  72 h PFA | 57175422 | 1348726 | 515373 | 0.82 | 1.29 |
| **Exp2 D-NT2 96 hpi datasets** | **Total deduplicated reads** | **Total read correction factor** | **Human mapped reads** | **HCMV mapped reads** | **Spike-in mapped reads** |
| Exp2 WT No Flavo | 60264248 | 1.033058809 | 37014680 | 22839819 | 409749 |
| Exp2 N1 No Flavo | 60509015 | 1.028879949 | 29487508 | 30640355 | 381152 |
| Exp2 NB No Flavo | 62797710 | 0.991381887 | 30167109 | 32194716 | 435885 |
| Exp2 N2 No Flavo | 65455076 | 0.95113345 | 32322068 | 32721383 | 411625 |
| Exp2 WT Flavo | 42060541 | 0.930844012 | 28924518 | 12660464 | 475559 |
| Exp2 N1 Flavo | 46703970 | 0.838297103 | 23392624 | 22572912 | 738434 |
| Exp2 NB Flavo | 36831123 | 1.063008661 | 16974762 | 19337239 | 519122 |
| Exp2 N2 Flavo | 31011577 | 1.2624899 | 15632816 | 14850432 | 528329 |
| **Exp2 D-NT2 96 hpi datasets** | **Corrected human reads** | **Corrected CMV reads** | **Corrected Spike-in reads** | **Spike-in correction factor** | **Final correction factor** |
| Exp2 WT No Flavo | 38238341 | 23594876 | 423295 | 0.97 | 1.00 |
| Exp2 N1 No Flavo | 30339106 | 31525247 | 392160 | 1.04 | 1.08 |
| Exp2 NB No Flavo | 29907125 | 31917258 | 432128 | 0.95 | 0.94 |
| Exp2 N2 No Flavo | 30742600 | 31122402 | 391510 | 1.05 | 1.00 |
| Exp2 WT Flavo | 26924214 | 11784917 | 442671 | 1.29 | 1.20 |
| Exp2 N1 Flavo | 19609969 | 18922807 | 619027 | 0.92 | 0.77 |
| Exp2 NB Flavo | 18044319 | 20555653 | 551831 | 1.03 | 1.10 |
| Exp2 N2 Flavo | 19736272 | 18748520 | 667010 | 0.85 | 1.08 |
| **Exp3 D-NT2 96 hpi datasets** | **Total deduplicated reads** | **Total read correction factor** | **Human mapped reads** | **HCMV mapped reads** | **Spike-in mapped reads** |
| Exp3 IE2F No Flavo | 59220261 | 0.946335622 | 29478650 | 28200439 | 1541172 |
| Exp3 IE2F No Flavo dTAG | 52864224 | 1.060116621 | 25778740 | 25900395 | 1185089 |
| Exp3 IE2F Flavo | 48042496 | 0.939085919 | 20856719 | 26092426 | 1093351 |
| Exp3 IE2F Flavo dTAG | 42189567 | 1.069364649 | 16755516 | 24067345 | 1366706 |
| Exp3 UL87^H^F No Flavo | 47972560 | 1.196102063 | 25723039 | 21985258 | 264263 |
| Exp3 UL87^H^F No Flavo dTAG | 66787596 | 0.859142737 | 35902204 | 30546481 | 338911 |
| Exp3 UL87^H^F Flavo | 35244066 | 1.217557177 | 18338700 | 16543094 | 362272 |
| Exp3 UL87^H^F Flavo  dTAG | 50579265 | 0.848404292 | 26073618 | 23935292 | 570355 |
| **Exp3 D-NT2 96 hpi datasets** | **Corrected human reads** | **Corrected HCMV reads** | **Corrected Spike-in reads** | **Spike-in correction factor** | **Final correction factor** |
| Exp3 IE2F No Flavo | 27896697 | 26687080 | 1458466 | 0.93 | 0.88 |
| Exp3 IE2F No Flavo dTAG | 27328471 | 27457439 | 1256333 | 1.08 | 1.15 |
| Exp3 IE2F Flavo | 19586251 | 24503030 | 1026751 | 1.21 | 1.14 |
| Exp3 IE2F Flavo dTAG | 17917756 | 25736768 | 1461507 | 0.85 | 0.91 |
| Exp3 UL87^H^F No Flavo | 30767380 | 26296612 | 316086 | 0.96 | 1.15 |
| Exp3 UL87^H^F No Flavo dTAG | 30845118 | 26243787 | 291173 | 1.04 | 0.90 |
| Exp3 UL87^H^F Flavo | 22328416 | 20142163 | 441087 | 1.05 | 1.28 |
| Exp3 UL87^H^F Flavo  dTAG | 22120969 | 20306804 | 483892 | 0.96 | 0.81 |
| **Exp4 D-NT2 96 hpi datasets** | **Total deduplicated reads** | **Total read correction factor** | **Human mapped reads** | **HCMV mapped reads** | **Spike-in mapped reads** |
| Exp4 WT No Flavo | 69201299 | 0.840193347 | 35443992 | 30647076 | 3110231 |
| Exp4 WT No Flavo  96 h PFA | 70970044 | 0.819253698 | 61454593 | 6050347 | 3465104 |
| Exp4 CK No Flavo | 43281428 | 1.343358426 | 21833868 | 19510245 | 1937315 |
| Exp4 WT Flavo | 57228521 | 1.042882285 | 27605480 | 25311101 | 4311940 |
| Exp4 WT Flavo 96 h PFA | 59359313 | 1.005446454 | 50652346 | 2394584 | 6312383 |
| Exp4 CK Flavo | 67940746 | 0.878450919 | 30689304 | 32675056 | 4576386 |
| Exp4 IE2F No Flavo | 55945308 | 0.948034775 | 27214406 | 25959387 | 2771515 |
| Exp4 IE2F No Flavo dTAG | 50130887 | 1.057992401 | 25893735 | 22450260 | 1786892 |
| Exp4 IE2F Flavo | 28583807 | 1.433129324 | 14856901 | 11875520 | 1851386 |
| Exp4 IE2F Flavo dTAG | 53344777 | 0.767915704 | 24672518 | 25130620 | 3541639 |
| **Exp4 D-NT2 96 hpi datasets** | **Corrected human reads** | **Corrected HCMV reads** | **Corrected Spike-in reads** | **Spike-in correction factor** | **Final correction factor** |
| Exp4 WT No Flavo | 29779806 | 25749469 | 2613195 | 0.95 | 0.80 |
| Exp4 WT No Flavo  96 h PFA | 50346903 | 4956769 | 2838799 | 0.87 | 0.71 |
| Exp4 CK No Flavo | 29330711 | 26209252 | 2602508 | 0.95 | 1.28 |
| Exp4 WT Flavo | 28789266 | 26396499 | 4496846 | 1.05 | 1.09 |
| Exp4 WT Flavo 96 h PFA | 50928222 | 2407626 | 6346763 | 0.74 | 0.75 |
| Exp4 CK Flavo | 26959047 | 28703433 | 4020130 | 1.17 | 1.03 |
| Exp4 IE2F No Flavo | 25800203 | 24610402 | 2627493 | 0.86 | 0.82 |
| Exp4 IE2F No Flavo dTAG | 27395375 | 23752204 | 1890518 | 1.19 | 1.26 |
| Exp4 IE2F Flavo | 21291860 | 17019156 | 2653276 | 1.01 | 1.45 |
| Exp4 IE2F Flavo dTAG | 18946414 | 19298198 | 2719680 | 0.99 | 0.76 |
| **Exp5 96 hpi datasets** | **Total deduplicated reads** | **Total read correction factor** | **Human mapped reads** | **HCMV mapped reads** | **Spike-in mapped reads** |
| Exp5 HFF WT Flavo | 66984819 | 1.072488708 | 26660302 | 39133820 | 1190697 |
| Exp5 HFF NB1 Flavo | 84328269 | 0.851914344 | 33688481 | 49296480 | 1343308 |
| Exp5 HFF NB2 Flavo | 61979301 | 1.159104102 | 20214085 | 40693200 | 1072016 |
| Exp5 D-NT2 WT Flavo | 70382630 | 1.063607569 | 42001281 | 26869507 | 1511842 |
| Exp5 D-NT2 NB1 Flavo | 85088924 | 0.879779582 | 46441589 | 36767310 | 1880025 |
| Exp5 D-NT2 NB2 Flavo | 62880274 | 1.190508457 | 30024885 | 31135414 | 1719975 |
| **Exp5 96 hpi datasets** | **Corrected human reads** | **Corrected HCMV reads** | **Corrected Spike-in reads** | **Spike-in correction factor** | **Final correction factor** |
| Exp5 HFF WT Flavo | 28592873 | 41970580 | 1277009 | 1.04 | 1.11 |
| Exp5 HFF NB1 Flavo | 28699700 | 41996378 | 1144383 | 1.16 | 0.99 |
| Exp5 HFF NB2 Flavo | 23430229 | 47167655 | 1242578 | 1.07 | 1.24 |
| Exp5 D-NT2 WT Flavo | 44672880 | 28578611 | 1608007 | 1.04 | 1.11 |
| Exp5 D-NT2 NB1 Flavo | 40858362 | 32347129 | 1654008 | 1.01 | 0.89 |
| Exp5 D-NT2 NB2 Flavo | 35744880 | 37066974 | 2047645 | 0.82 | 0.97 |
| **Exp6 D-NT2 96 hpi datasets** | **Total deduplicated reads** | **Total read correction factor** | **Human mapped reads** | **HCMV mapped reads** | **Spike-in mapped reads** |
| Exp6 UL87^H^F No Flavo | 44903162 | 1.057280922 | 23311553 | 21115707 | 475902 |
| Exp6 UL87^H^F No Flavo dTAG | 50047351 | 0.94860678 | 26577382 | 22906701 | 563268 |
| Exp6 UL87^H^F Flavo | 51390319 | 0.897431314 | 24802632 | 25894365 | 693322 |
| Exp6 UL87^H^F Flavo  dTAG | 40848244 | 1.129039513 | 21439375 | 18840223 | 568646 |
| **Exp6 D-NT2 96 hpi datasets** | **Corrected human reads** | **Corrected CMV reads** | **Corrected Spike-in reads** | **Spike-in correction factor** | **Final correction factor** |
| Exp6 UL87^H^F No Flavo | 24646860 | 22325234 | 503162 | 1.03 | 1.09 |
| Exp6 UL87^H^F No Flavo dTAG | 25211485 | 21729452 | 534320 | 0.97 | 0.92 |
| Exp6 UL87^H^F Flavo | 22258659 | 23238414 | 622209 | 1.02 | 0.91 |
| Exp6 UL87^H^F Flavo  dTAG | 24205902 | 21271356 | 642024 | 0.98 | 1.11 |

***** No Flavo and Flavo datasets were normalized separately in the experiments that have both No Flavo and Flavo datasets
