# Supplementary material for: Cell type differences in human cytomegalovirus transcription and epigenetic regulation with insights into major immediate-early enhancer-promoter control
Source: PLoS Pathog. 2025 Aug 4;21(8):e1013374. doi: 10.1371/journal.ppat.1013374 (PMC12333995; doi:10.1371/journal.ppat.1013374)
Supplement: S2 Table — (DOCX) [file ppat.1013374.s014.docx]

**S2 TABLE. DFF-Seq Datasets***

| **D-NT2 DFF-Seq Exp1 datasets** | **Total deduplicated reads** | **Human mapped reads** | **HCMV reads** |
| --- | --- | --- | --- |
| Pol II Rep1 | 37933334 | 22301055 | 15632279 |
| Pol II Rep2 | 39917888 | 24504991 | 15412897 |
| H3K4me3 Rep1 | 46333102 | 23597045 | 22736057 |
| H3K4me3 Rep2 | 50936152 | 25203325 | 25732827 |
| **D-NT2 DFF-Seq Exp2 datasets** | **Total deduplicated reads** | **Human mapped reads** | **CMV reads** |
| Pol II Rep1 | 45142585 | 28175949 | 16966636 |
| Pol II Rep2 | 51091425 | 33225843 | 17865582 |
| TBP Rep1 | 59920903 | 36652931 | 23267972 |
| TBP Rep2 | 51857533 | 31326252 | 20531281 |
| **D-NT2 DFF-Seq Exp3 datasets** | **Total deduplicated reads** | **Human mapped reads** | **HCMV reads** |
| Pol II Rep1 | 73420305 | 41172709 | 32247596 |
| Pol II Rep2 | 69082620 | 37593465 | 31489155 |
| TBP Rep1 | 74461931 | 39116959 | 35344972 |
| TBP Rep2 | 76229565 | 37182979 | 39046586 |
| H3K4me3 Rep1 | 69900883 | 25288157 | 44612726 |
| H3K4me3 Rep2 | 67914261 | 25550535 | 42363726 |
| H3K27Ac | 67639910 | 55248728 | 12391182 |
| IE2 Rep1 | 108606520 | 19753426 | 88853094 |
| IE2 Rep2 | 66091535 | 41516051 | 24575484 |
| **DFF-Seq Exp4 datasets** | **Total deduplicated reads** | **Human mapped reads** | **HCMV reads** |
| D-NT2 Pol II | 52239989 | 22812450 | 29427539 |
| D-NT2 TBP | 58176369 | 31546019 | 26630350 |
| D-NT2 H3K4me3 | 41017162 | 9974626 | 31042536 |
| D-NT2 Cross-linking  Pol II Rep1 | 54832160 | 41864461 | 12967699 |
| D-NT2 Cross-linking  Pol II Rep2 | 59835274 | 46283203 | 13552071 |
| D-NT2 Cross-linking TBP Rep1 | 47458770 | 34210067 | 13248703 |
| D-NT2 Cross-linking TBP Rep2 | 49054708 | 35363676 | 13691032 |
| D-NT2 Cross-linking H3K4me3 Rep1 | 45985304 | 20933287 | 25052017 |
| D-NT2 Cross-linking H3K4me3 Rep2 | 37337170 | 16596799 | 20740371 |
| HFF Pol II Rep1 | 53900115 | 41132678 | 12767437 |
| HFF Pol II Rep2 | 62529310 | 45457271 | 17072039 |
| HFF TBP Rep1 | 53161870 | 39878908 | 13282962 |
| HFF TBP Rep2 | 54140521 | 39359599 | 14780922 |
| HFF H3K4me3 Rep1 | 48259216 | 43750974 | 4508242 |
| HFF H3K4me3 Rep2 | 43656893 | 39581650 | 4075243 |
| **DFF-Seq Exp5 datasets** | **Total deduplicated reads** | **Human mapped reads** | **HCMV reads** |
| D-NT2 TBP | 88011558 | 70848526 | 17163032 |
| D-NT2 H3.3 | 60568626 | 48147791 | 12420835 |
| D-NT2 H3K4me3 | 75518299 | 33355821 | 42162478 |
| HFF TBP | 93784962 | 77520131 | 16264831 |
| HFF H3.3 | 52972237 | 48785965 | 4186272 |
| HFF H3K4me3 | 56561680 | 52650516 | 3911164 |

*All infections carried out for 96 h
