# Supplementary material for: Cell type differences in human cytomegalovirus transcription and epigenetic regulation with insights into major immediate-early enhancer-promoter control
Source: PLoS Pathog. 2025 Aug 4;21(8):e1013374. doi: 10.1371/journal.ppat.1013374 (PMC12333995; doi:10.1371/journal.ppat.1013374)
Supplement: S3 Table — (DOCX) [file ppat.1013374.s015.docx]

**S3 TABLE. RNA-Seq Datasets***

| **RNA-Seq datasets** | **Total deduplicated reads** | **Human mapped reads** | **HCMV reads** |
| --- | --- | --- | --- |
| Mock Rep1 | 157300260 | 157283594 | 16666 |
| Mock Rep2 | 147379086 | 147361531 | 17555 |
| WT Rep1 | 156876928 | 137586486 | 19290442 |
| WT Rep2 | 174499190 | 154176204 | 20322986 |
| WT PFA Rep1 | 146229712 | 135810874 | 10418838 |
| WT PFA Rep2 | 160865936 | 149351959 | 11513977 |
| N1 Rep1 | 170594666 | 136275864 | 34318802 |
| N1 Rep2 | 182758328 | 144994907 | 37763421 |
| NB Rep1 | 176382292 | 145995517 | 30386775 |
| NB Rep2 | 128854842 | 107530610 | 21324232 |
| CK Rep1 | 168332248 | 151812190 | 16520058 |
| CK Rep2 | 135368476 | 122260255 | 13108221 |

*****D-NT2 infections carried out for 96 h
