# Supplementary material for: Cell type differences in human cytomegalovirus transcription and epigenetic regulation with insights into major immediate-early enhancer-promoter control
Source: PLoS Pathog. 2025 Aug 4;21(8):e1013374. doi: 10.1371/journal.ppat.1013374 (PMC12333995; doi:10.1371/journal.ppat.1013374)
Supplement: S4 Table — (DOCX) [file ppat.1013374.s016.docx]

| **S4 Table. Nucleic Acid Reagents** | | |  |
| --- | --- | --- | --- |
| **A.** **Oligonucleotides and Gene Blocks** | | |  |
| Oligo Name | Direction | Sequence (5’-to-3’) | |
| **BAC Construction Oligos** | | |  |
| Towne MIE Enhancer-**galK** | Forward | GCGATCTGACGGTTCACTAAACGAGCTCTGCTTATATAGACCT  CCCACCGTAC**CCTGTTGACAATTAATCATCGGCA** | |
| Towne MIE Enhancer-**galK** | Reverse | TGACCGCCATGTTGACATTGATTATTGACTAGTTATTAATAGTA  ATCAATTACGGGGTCATTAGTT**TCAGCACTGTCCTGCTCCTT** | |
| **Gene Blocks (dsDNA)** | | |  |

| Towne N1 1 | GGTCAAAACAGCGTGGATGGCGTCTCCAGGCGATCTGACGGTTCACTAAACGAGCTCTGCTTATATAGACCTCCCACCGTACACGCCTAC**a**G**aaa**ATTTGCGTCAACGGGGCGGGGTTATTACGACATTTTGGAAAGTCCCGTTGATTTTGGTGCCAAAACAAACTCCCATTGACGTCAATGGGGTGGAGACTTGGAAATCCCCGTGAGTCAAACCGCTATCCACGCCCATTGGTGTACTGCCAAAACCGCATCACCATGGTAATAGCGATGACTAATACGTAGATGTACTGCCAAGTAGGAAAGTCCCGTAAGGTCATGTACTGGGCATAATGCCAGGCGGGCCATTTACCGTCATTGACGTC**AATAGGGGGCGGACTTGGCATATGATACACTTGATGTACTGCCAAGTGGGCAGTTTACC** | |  |
| --- | --- | --- | --- |
| Towne N2 1 | GGTCAAAACAGCGTGGATGGCGTCTCCAGGCGATCTGACGGTTCACTAAACGAGCTCTGCTTATATAGACCTCCCACCGTACACGCCTACCGCCCATTTGCGTCAACG**ttt**C**t**GGGTTATTACGACATTTTGGAAAGTCCCGTTGATTTTGGTGCCAAAACAAACTCCCATTGACGTCAATGGGGTGGAGACTTGGAAATCCCCGTGAGTCAAACCGCTATCCACGCCCATTGGTGTACTGCCAAAACCGCATCACCATGGTAATAGCGATGACTAATACGTAGATGTACTGCCAAGTAGGAAAGTCCCGTAAGGTCATGTACTGGGCATAATGCCAGGCGGGCCATTTACCGTCATTGACGTC**AATAGGGGGCGGACTTGGCATATGATACACTTGATGTACTGCCAAGTGGGCAGTTTACC** | |  |
| Towne NB 1 | GGTCAAAACAGCGTGGATGGCGTCTCCAGGCGATCTGACGGTTCACTAAACGAGCTCTGCTTATATAGACCTCCCACCGTACACGCCTAC**a**G**aaa**ATTTGCGTCAACG**ttt**C**t**GGGTTATTACGACATTTTGGAAAGTCCCGTTGATTTTGGTGCCAAAACAAACTCCCATTGACGTCAATGGGGTGGAGACTTGGAAATCCCCGTGAGTCAAACCGCTATCCACGCCCATTGGTGTACTGCCAAAACCGCATCACCATGGTAATAGCGATGACTAATACGTAGATGTACTGCCAAGTAGGAAAGTCCCGTAAGGTCATGTACTGGGCATAATGCCAGGCGGGCCATTTACCGTCATTGACGTC**AATAGGGGGCGGACTTGGCATATGATACACTTGATGTACTGCCAAGTGGGCAGTTTACC** | |  |
| Towne N1 N2 NB 2 | **AATAGGGGGCGGACTTGGCATATGATACACTTGATGTACTGCCAAGTGGGCAGTTTACC**GTAAATACTCCACCCATTGACGTCAATGGAAAGTCCCTATTGGCGTTACTATGGGAACATACGTCATTATTGACGTCAATGGGCGGGGGTCGTTGGGCGGTCAGCCAGGCGGGCCATTTACCGTAAGTTATGTAACGCGGAACTCCATATATGGGCTATGAACTAATGACCCCGTAATTGATTACTATTAATAACTAGTCAATAATCAATGTCAACATGGCGGTCATATTGGACATGAGCCAATATAAATGTACAT | |  |
| Towne CK 1 | GGTCAAAACAGCGTGGATGGCGTCTCCAGGCGATCTGACGGTTCACTAAACGAGCTCTGCTTATATAGACCTCCCACCGTACACGCCTACCGCCCATTTG**gc**TCAACGGGGCGGGGTTATTACGACATTTT**cc**AAAG**g**CC**t**GTTGATTTTGGTGCCAAAACAAACTCCCATTGA**gc**TCAATGGGGTGGAGACTTG**c**AA**ggta**CCGTGAGTCAAACCGCTATCCACGCCCATTGGTGTACTGCCAAAACCGCATCACCATGGTAATAGCGATGACTAATACGTAGATGTACTGCCAAGTAG**cc**A**ga**TC**t**CGTAAGGTCATGTACTGGGCATAATGCCAGGCGGGCCATTTACCGTCATTGA**gc**TC**AATAGGGGGCGGACTTGGCATATGATACACTTGATGTACTGCCAAGTGGGCAGTTTACC** | |  |
| Towne CK 2 | AATAGGGGGCGGACTTGGCATATGATACACTTGATGTACTGCCAAGTGGGCAGTTTACCGTAAATACTCCACCCATTGAgcTCAATGccAgaTCtCTATTGGCGTTACTATGGGAACATACGTCATTATTGAgcTCAATGGGCGGGGGTCGTTGGGCGGTCAGCCAGGCGGGCCATTTACCGTAAGTTATGTAACGCGGAACTCCATATATGGGCTATGAACTAATGACCCCGTAATTGATTACTATTAATAACTAGTCAATAATCAATGTCAACATGGCGGTCATATTGGACATGAGCCAATATAAATGTACAT | |  |
| **qPCR** | | |  |
| MIE | Forward | GCATTGGAACGCGGATTC |  |
|  | Reverse | CAGGATTATCAGGGTCCATCTTTC |  |
| IE1 Exon 4 | Forward | AAGGTCTTTGCCCAGTACATTC |  |
|  | Reverse | GCCCGTAGGTCATCCACAC |  |
| UL99 | Forward | GGTGAGCCCCTGAAAGATG |  |
|  | Reverse | GGAAGTCGGAGGGATGTTG |  |
| GAPDH | | Forward | CTGTTGCTGTAGCCAAATTCGT |
|  | | Reverse | ACCCACTCCTCCACCTTTGAC |
| PDIA2 | | Forward | CTGGCCAAGGTGGATGG |
|  | | Reverse | GGAAGAACTTGAGCGTAGGG |
| CCDC88B | | Forward | CAGTTGCTGGGAGGAGAGA |
|  | | Reverse | GGGCCCTCCTGTCTCAA |
| KLHL17 | | Forward | CACGTGGATGCCGAGAG |
|  | | Reverse | GCAGGTGGAACTTCAGG |
| RGS11 | | Forward | GGCGTCATCCAGGACATAG |
|  | | Reverse | ACTGGGTCAACATCGACAG |
| TTYH1 | | Forward | CGAGCTCTGGCCAACAT |
|  | | Reverse | CTGCGCTGAAGGGAACT |
| PTN | | Forward | GAGTTGGAAACGTCCTCTCTG |
|  | | Reverse | TACCTGGACTCAGCGGTAG |
| RFX4 | | Forward | CCGGATGGAACCTTGTTTGA |
|  | | Reverse | GTGTAGCACGTGTTGGCT |
| CRABP1 | | Forward | CTTGCGAGCTCAGAGTGTG |
|  | | Reverse | CTTGAGCAGCTCGTCGAAAT |
| LRAT | | Forward | CATGCTGGAGGTGGTGTC |
|  | | Reverse | GGCGCCCGAACTAAAGAG |
| BTBD17 | | Forward | TCCGGATAAGGGTGTGGTAT |
|  | | Reverse | AACTCCGAGTACCTGGTTGA |
